# Photocatalytic Synthesis of 3,4-Dihydroquinolone from Tetrahydroquinolines by a High-Throughput Microfluidic System and Insights into the Role of Organic Bases

**DOI:** 10.3390/molecules31010026

**Published:** 2025-12-22

**Authors:** Shuyuan Ding, Tian-Yu Sun, Heming Jiang, Yun-Dong Wu, Xinhao Zhang

**Affiliations:** 1 Key Laboratory of Computational Chemistry and Drug Design, State Key Laboratory of Chemical Oncogenomics, Shenzhen Key Laboratory of Chemical Genomics, School of Chemical Biology and Biotechnology, Peking University Shenzhen Graduate School, Shenzhen 518055, China; 2 Institute of Chemical Biology, Shenzhen Bay Laboratory, Shenzhen 518132, China; 3 College of Chemistry and Molecular Engineering, Peking University, Beijing 100871, China

**Keywords:** 3,4-dihydroquinolone, photocatalysis, high-throughput, microfluidics, DFT

## Abstract

3,4-dihydroquinolone and its derivatives are structural motifs found in diverse pharmacologically active compounds. Direct oxidation of tetrahydroquinolines represents the most efficient synthetic route to 3,4-dihydroquinolone. However, the reaction conditions reported in previous studies were either relatively harsh or complex. We also attempted previously reported photocatalytic oxidation methods for the α-carbonylation of amines, but these approaches failed to efficiently produce 3,4-dihydroquinolone. Herein, we present an efficient photocatalytic oxidation methodology facilitated by our in-house high-throughput microfluidic system, which can be carried out under mild conditions with a short reaction time. Moreover, a new reaction mechanism, in which the organic base DBU serves a dual role as both an electron donor and a hydrogen atom transfer (HAT) mediator, is proposed and supported by DFT calculations.

## 1. Introduction

**Figure 1:**
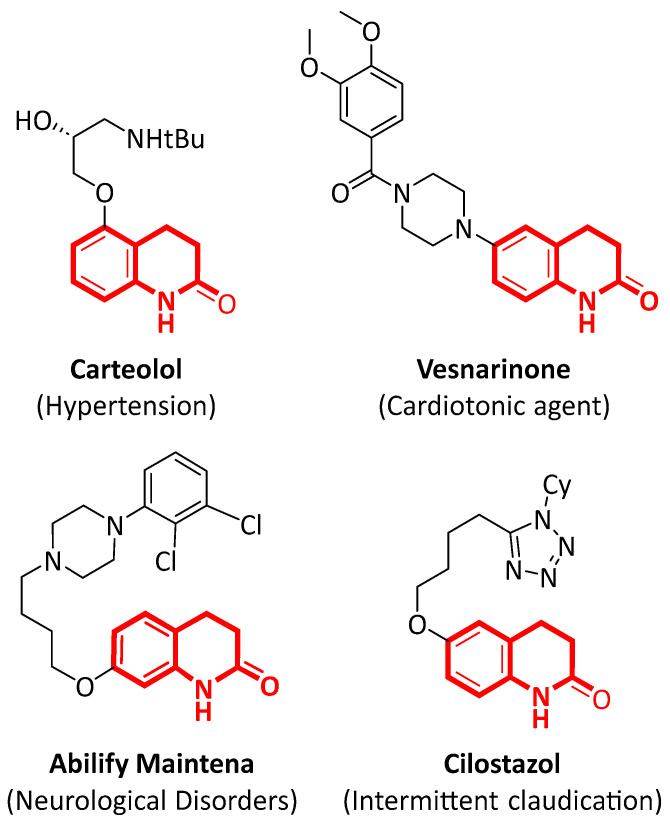
Pharmaceuticals containing 3,4-dihydroquinolone moieties.

3,4-dihydroquinolone and its derivatives represent a class of nitrogen-containing heterocycles that serve as key structural motifs in a wide range of biologically active molecules [1,2,3,4,5]. Owing to their pronounced pharmacological relevance, these compounds have attracted considerable attention in the fields of pharmaceutical research (Figure 1) and natural product chemistry in recent years [6,7,8,9,10,11,12,13].

**Figure 2:**
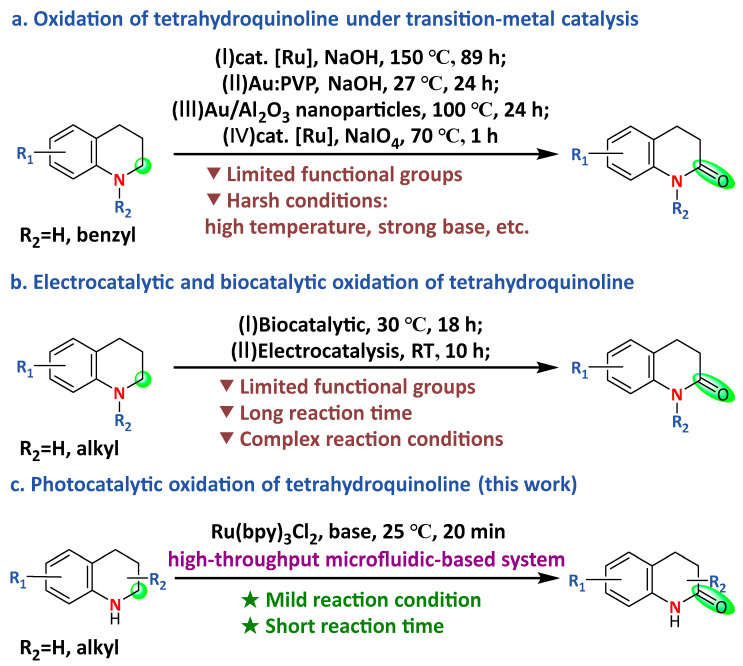
Overview of previous studies and the present work. Oxidation of tetrahydroquinoline under transition-metal catalysis (**a**), electrocatalytic and biocatalytic oxidation of tetrahydroquinoline (**b**), and photocatalytic oxidation of tetrahydroquinoline (this work) (**c**).

Although a variety of multi-step strategies have been established for the synthesis of 3,4-dihydroquinolones, these methods often suffer from a reliance on pre-functionalized substrates, sophisticated multi-component/multi-catalyst reaction systems, or harsh reaction conditions, which limits their practicality [14,15,16,17,18]. In contrast, the direct oxidation of readily available tetrahydroquinolines via transition-metal catalysis represents the most straightforward and atom-economical route to access these valuable scaffolds (Figure 2a) [19,20,21,22]. While these methods successfully convert tetrahydroquinoline to 3,4-dihydroquinolone, they often require harsh reaction conditions, such as high temperatures, long reaction time and strong bases, which limit their practical applications. Given the structural and functional importance of 3,4-dihydroquinolone, developing milder and more efficient synthetic methods remains critically important.

In recent years, biocatalysis, electrocatalysis, and visible-light photocatalysis (400–800 nm) have attracted growing attention due to their environmental friendliness, high selectivity, and mild reaction conditions [23,24,25]. For instance, Chen’s group demonstrated the biocatalytic oxidation of tetrahydroquinoline to 3,4-dihydroquinolone using whole cells in a phosphate buffer (KH_2_PO_4_-Na_2_HPO_4_) at 30 °C for 18 h (Figure 2b). Despite its success, this method suffers from a limited substrate scope and long reaction time [23]. He’s group recently employed electrocatalytic conditions, where tetrahydroquinoline reacted with TEMPO in a DMF solution containing K_2_CO_3_ and NaI over 10 h to generate 3,4-dihydroquinolone (Figure 2b) [24]. In photocatalytic oxidation, the α-carbonylation of amines has been successfully achieved by Das’s and Lee’s group [26,27]. However, these approaches failed to achieve an efficient transformation of tetrahydroquinoline into 3,4-dihydroquinolone.

In this study, we identified a mild photocatalytic condition for the efficient synthesis of 3,4-dihydroquinolone from tetrahydroquinoline in the presence of an organic base (Figure 2c). Organic bases were employed for two key reasons. First, organic bases generally exhibit superior solubility in common organic solvents (e.g., MeCN and DMF) compared to many inorganic bases, ensuring homogeneous reaction conditions which are often critical for reproducibility and efficiency in photocatalysis. Second, certain organic bases, particularly tertiary amines, can act as electron donors to facilitate the photocatalytic cycle in many photoredox systems [28,29,30,31].

Guided by these considerations, the reaction conditions were optimized using our in-house high-throughput microfluidic system, and the reaction mechanism along with the role of organic bases were investigated using density functional theory (DFT) studies [32]. This photocatalytic methodology features mild reaction conditions and short reaction time, making it highly promising for the efficient synthesis of 3,4-dihydroquinolone.

## 2. Results and Discussion

### 2.1. The Optimization of Reaction Conditions

**Figure 3:**
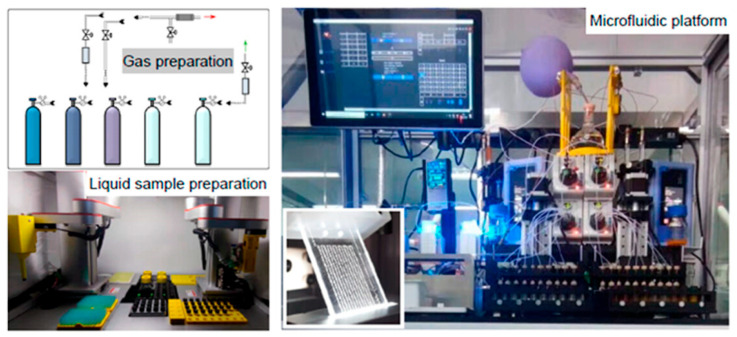
Main components of the microfluidic high-throughput screening chemical robot, including sample preparation module (gas preparation and liquid sample preparation) and reaction operation module (microfluidic platform).

Previously, we developed a high-throughput microfluidic system. This system was successfully applied to amidation reactions (Figure 3) [32,33]. Based on our previous work, we employed a two-step optimization strategy: discrete variables were optimized first, then continuous variables.

**Figure 4:**
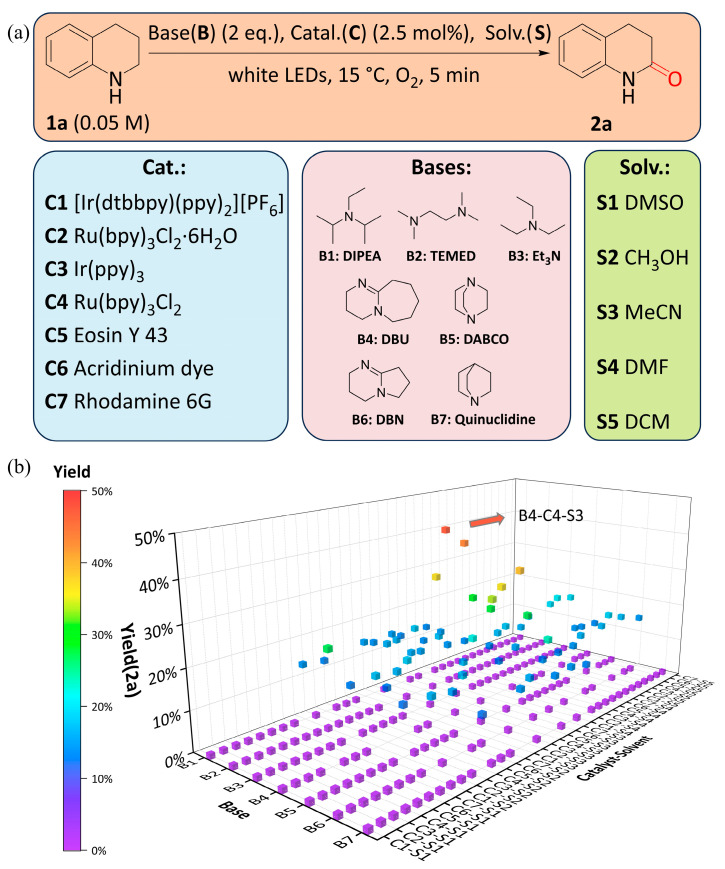
(**a**) The photocatalytic reaction investigated in this study; (**b**) the heatmap of yields. The reaction parameters were set to a liquid flow rate of *v_l_* = 20 μL/min and a gas flow rate of *v_g_* = 0.04 mL/min.

Tetrahydroquinoline (**1a**) was chosen as the model substrate, and reactions were performed using a range of catalysts (2.5 mol%), different bases (2 eq.), and various solvents at a fixed concentration of **1a** (0.05 M) (Figure 4a). To minimize reagent consumption, each reaction was performed with only 100 μL of solution.

#### 2.1.1. The Optimization of Discrete Variables

For the optimization of discrete variables in the first step, as shown in Figure 4a, we selected seven widely used organic bases, seven frequently employed photocatalysts, and five commonly used solvents, resulting in a total of 7 × 7 × 5 = 245 reaction conditions. Reactions were carried out under irradiation with white LEDs for 5 min at 15 °C in an O_2_ atmosphere. The corresponding reaction yields are visualized as a heatmap in Figure 4b. Among the tested conditions, 1,8-diazabicyclo[5.4.0]undec-7-ene (DBU, **B4**) as the base, Ru(bpy)_3_Cl_2_ (**C4**) as the photocatalyst, and acetonitrile (MeCN, **S3**) as the solvent were identified as the optimal reaction conditions, with a yield of 42.7%.

#### 2.1.2. The Optimization of Continuous Variables

Following the optimization of discrete variables (**B4**-**C4**-**S3**), further optimization was carried out on continuous variables, including reagent loading, reaction time, concentration, and temperature. A Gaussian process regression (GPR) model—a machine learning (ML) approach previously utilized in our work [34,35,36,37]—was employed to predict the optimal values of continuous variables.

**Figure 5:**
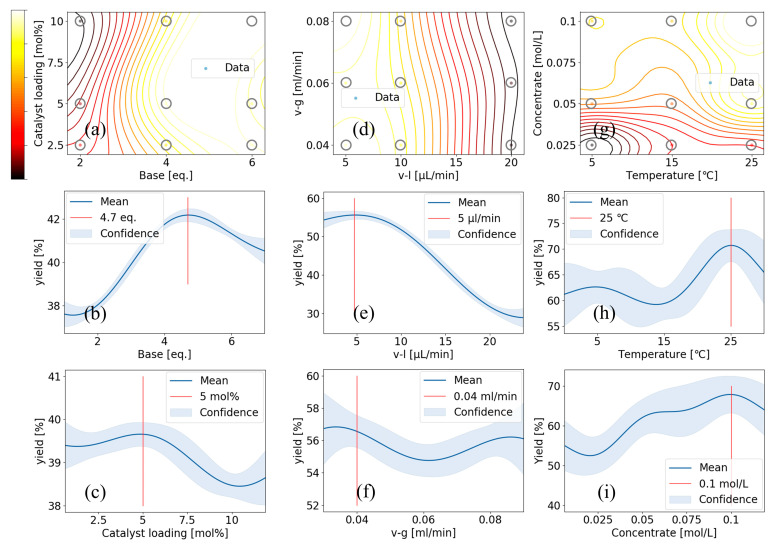
Screening of continuous variables including base and catalyst loading, reaction time, concentration and temperature. (v-l: liquid flow rate, v-g: gas flow rate) Estimated yield from Table S1 (entries 1–9) (**a**), predicted yield for base loading (**b**) shown in the yellow region in (**a**), predicted yield for catalyst loading (**c**) shown in the yellow ring in (**a**), estimated yield from Table S2 (entries 1–9) (**d**), predicted yield for liquid flow rate (**e**) shown in the yellow region in (**d**), predicted yield for gas flow rate (**f**) shown in the yellow ring in (**d**), estimated yield from Table S3 (entries 1–9) (**g**), predicted yield for temperatures (**h**) shown in the yellow region in (**g**), predicted yield for concentrate of **1a** (**i**) shown in the yellow ring in (**g**).

As shown in Figure 5a, we first optimized the loading of the organic base **B4** and catalyst **C4**. We selected the conditions as a combination of base loading (2, 4, and 6 eq.) and catalyst loading (2.5, 5, and 10 mol%). The 3 × 3 experimental design generated nine experimental points: (2 eq., 2.5 mol%), (2 eq., 5 mol%), (2 eq., 10 mol%), (4 eq., 2.5 mol%), (4 eq., 5 mol%), (4 eq., 10 mol%), (6 eq., 2.5 mol%), (6 eq., 5 mol%), and (6 eq., 10 mol%), which were subsequently fitted using GPR to generate the curves in Figure 5a (different colors represent varying yields, with the color bar on the left ranging from black through red and yellow to white, corresponding to increasing yields from low to high). The predicted results indicated that 4.7 equivalents of base and 5 mol% catalyst were identified as the optimal reaction conditions (as highlighted by the red line in Figure 5b,c).

After determining the appropriate base equivalents and catalyst loading, we further optimized the reaction time with the same procedure. In our microfluidic system, parameters such as gas and liquid flow rates are tunable and have a direct impact on the reaction time. We selected the conditions as a combination of liquid flow rates (5, 10, and 20 μL/min) and gas flow rates (0.04, 0.06, and 0.08 mL/min). As shown in Figure 5d–f, the optimal flow rates were determined to be 0.04 mL/min for the liquid and 5 μL/min for the O_2_.

Finally, with the base and catalyst loadings as well as the gas and liquid flow rates fixed, we proceeded to optimize the concentration and temperature. We selected the conditions as a combination of temperature (5, 15, and 25 °C) and substrate concentration (0.025, 0.05, and 0.1 M). As shown in Figure 5g–i, the optimal reaction conditions were determined to be a temperature of 25 °C and a substrate concentration of 0.1 M.

By applying the optimized reaction conditions described above, the final yield was improved from 42.7% to 76.1%, demonstrating the effectiveness of the GPR model.

### 2.2. The Substrate Scope of the Reaction

**Figure 6:**
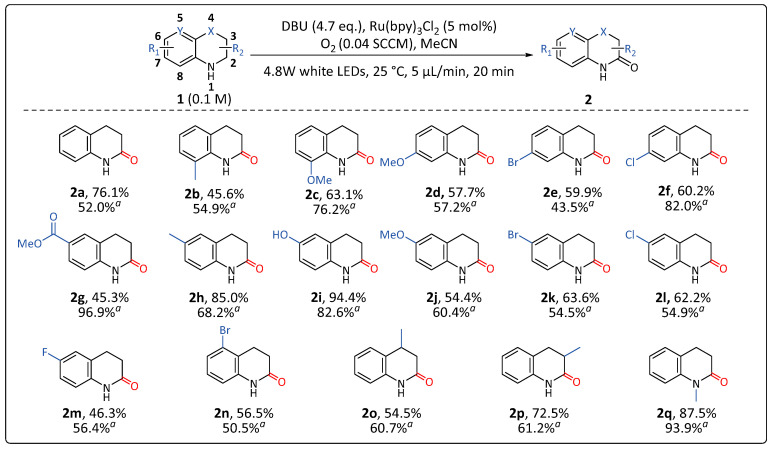
Substrate scope. The microfluidic system parameters were set to a liquid flow rate of *v_l_* = 5 μL/min and a gas flow rate of *v_g_* = 0.04 mL/min (standard cubic centimeter per minute, SCCM). *^a^* Flask reaction conditions: experiment consisted of a 4 mL clear glass vial, a magnetic stirrer, and an oxygen balloon with irradiation under 20 W blue LED at 25 °C for 20 h.

With the optimized reaction conditions in hand, we next investigated the substrate scope of this photocatalytic reaction (Figure 6). A total of 27 commercially available substrates were evaluated, involving tetrahydroquinoline derivatives (**1a-y**) and several heterocyclic substrates (**1aa-ac**) (see Supporting Information for more details). For tetrahydroquinoline derivatives (**1a-y**), most substrates yielded the corresponding products in yields exceeding 45%, except for a few cases (**1r-y**), with the substituent electronic effects and their positions significantly influencing the yields.

At the **C8** position, methyl (**2b**) and methoxy (**2c**) substituents were well tolerated, yielding the corresponding products in 50–63% yields; at the **C7** position, methoxy (**2d**), bromo (**2e**), and chloro (**2f**) groups resulted in yields of 57–60%.

At the **C6** position, a variety of substituents—including carboxylic acid methyl ester (**2g**), methyl (**2h**), hydroxy (**2i**), methoxy (**2j**), and halogens (**2k-m**; Br, Cl, and F)—were well tolerated, providing the corresponding products in 46–94% yields. Additionally, the **C5**-bromo substituent (**2n**) yielded the desired product at 57% yield.

Alkyl substituents such as 4-methyl (**2o**) and 3-methyl (**2p**) resulted in 55% and 73% yield, respectively. The N-methyl tetrahydroquinoline (**1q**) was well tolerated (up to 88% yield).

Among methoxy-substituted substrates, reactivity followed the order 8-OMe > 7-OMe > 6-OMe, yielding the corresponding dihydroquinolones (**2c**, **2d**, and **2j**) in 55–65% yield. Notably, the successful transformation of the 6-hydroxy-substituted substrate (**1i**) provides a potential synthetic route to the cardiovascular agent—cilostazol (see Figure 1) [38]. Moreover, it was noteworthy that halogen substituents (Cl and Br), despite their generally poor compatibility with many transition metal-catalyzed conditions [19,20,21], were well tolerated under the present photocatalytic conditions. The halogen-substituted substrates can undergo subsequent dehalogenative coupling to afford various derivatives. The successful transformation of the 6-bromo-substituted substrate (**1k**) provides a potential synthetic route to the drug—vesnarinone (see Figure 1) [7]. In contrast, electron-withdrawing groups are unfavorable to the reaction (see Supporting Information for full details).

To evaluate the practical applicability of the microfluidic system, the substrates shown in Figure 6 were subjected to a conventional batch setup (flask reaction) under the optimized reaction conditions. The yield variations between batch and flow systems may stem from several factors, including differences in local concentration gradients, mixing efficiency, and possible oxygen limitation due to gas–liquid dissolution. However, we prioritized the rapid screening capability of the system, emphasizing throughput and efficiency. Overall, good performance was achieved, as indicated by the yield data labeled “*^a^*” in the upper right corner in Figure 6, which closely matched the yields obtained using our in-house microfluidic system. This consistency validates the feasibility of our strategy.

### 2.3. The Mechanistic Study of the Reaction

**Figure 7:**
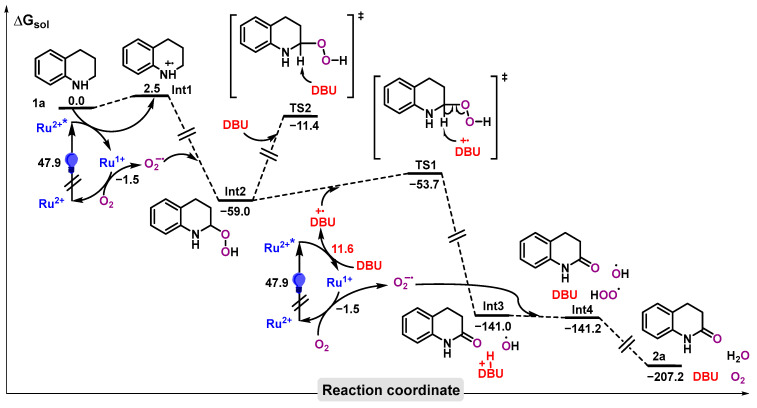
The PES of the photocatalytic synthesis of 3,4-dihydroquinolone from tetrahydroquinoline (kcal/mol). The symbol ‡ represents activated complex.

To elucidate the underlying mechanism of this photocatalytic reaction, we performed density functional theory (DFT) calculations (see SI for more computational details). The potential energy surface (PES) is shown in Figure 7. The photocatalyst [Ru(bpy)_3_]^2+^ is initially excited to its excited state [Ru(bpy)_3_]^2+*^ under irradiation, which subsequently undergoes a single-electron transfer (SET) with substrate **1a** to generate radical cation intermediate **Int1** and [Ru(bpy)_3_]^+^.

[Ru(bpy)_3_]^+^ can then react with O_2_ to form superoxide radical anion (O_2_^•−^) and regenerate the photocatalyst [Ru(bpy)_3_]^2+^ [25]. The radical anion O_2_^•−^ can subsequently react with the radical cation **Int1** to yield **Int2**. This type of reaction process has been proposed in previous studies [39]. DFT calculations suggest that DBU can also undergo a SET with the excited state [Ru(bpy)_3_]^2+*^ to generate the radical cation DBU^•+^ [40], with this step being endergonic by 11.6 kcal/mol. DBU^•+^ is capable of abstracting the hydrogen atom adjacent to the nitrogen atom in **Int2** via a hydrogen atom transfer (HAT) transition state **TS1**, which proceeds with a low energy barrier and leads to the formation of 3,4-dihydroquinolone, DBU^+^-H, and HO^•^.

The overall activation barrier for this HAT step is calculated to be 16.9 kcal/mol, as determined by the following equation: ΔG^‡^(overall) = ΔG^‡^(TS1) − ΔG(Int2) + 11.6 kcal/mol = −53.7 kcal/mol − (−59.0 kcal/mol) + 11.6 kcal/mol. Finally, in the presence of an additional molecule of O_2_^•−^, DBU^+^-H and HO^•^ can undergo a reaction to regenerate DBU, along with the formation of O_2_ and H_2_O. The activation barrier for direct deprotonation of the same C-H bond in **Int2** by neutral DBU via **TS2** (47.6 kcal/mol) is significantly higher in energy than that of the HAT pathway involving DBU^•+^, and therefore this pathway can be ruled out. The HAT step by DBU^•+^ thus constitutes the rate-determining step (RDS) on the PES.

### 2.4. The Role of Organic Bases

To validate the proposed role of the base as depicted in Figure 7, we carried out further DFT calculations on the seven organic bases used in the experimental screening (Figure 4a). As mentioned above, for DBU, the activation barrier of the RDS (ΔG^‡^_RDS_) comprises two contributions: the free energy required for the excited-state photocatalyst [Ru(bpy)_3_]^2+*^ to oxidize the organic base to its radical cation (ΔG_ox._ = 11.6 kcal/mol), and the activation barrier for the HAT step by the resulting base^•+^ (ΔG^‡^_HAT_ = 5.3 kcal/mol). ΔG_ox._ and ΔG^‡^_HAT_ were also calculated for the other six organic bases, respectively. As shown in Table 1, a general trend observed for **B1**–**B7** is that higher ΔG_ox._ values correlate with lower corresponding ΔG^‡^_HAT_ values. This is because a higher ΔG_ox._ value indicates a greater resistance to oxidation, leading to the formation of a less stable base^•+^. As a result, the less stable base^•+^ is more prone to undergoing the HAT process.

When ΔG_ox._ < 0 kcal/mol, the concentration of the base^•+^ in solution is considered sufficiently high; in such cases, ΔG^‡^_RDS_ was assumed to be equal to ΔG^‡^_HAT_, as observed for DIPEA (**B1**), TEMED (**B2**), and DABCO (**B5**). In contrast, when ΔG_ox._ > 0 kcal/mol, the concentration of base^•+^ is considered low, and ΔG^‡^_RDS_ was calculated as the sum of ΔG_ox._ and ΔG^‡^_HAT_. Based on this treatment, we obtained the ΔG^‡^_RDS_ for **B1**–**B7**. Since ΔG_ox._ and ΔG^‡^_HAT_ are negatively correlated, the differences in the calculated ΔG^‡^_RDS_ (16.9~21.1 kcal/mol) for these seven bases are smaller than those in the ΔG_ox._ (−3.0~11.6 kcal/mol) and ΔG^‡^_HAT_ (4.0~21.1 kcal/mol). Notably, DBU showed the lowest ΔG^‡^_RDS_ (16.9 kcal/mol) among the seven bases calculated, aligning well with its identification as the optimal base under the experimentally optimized conditions.

**Figure 8:**
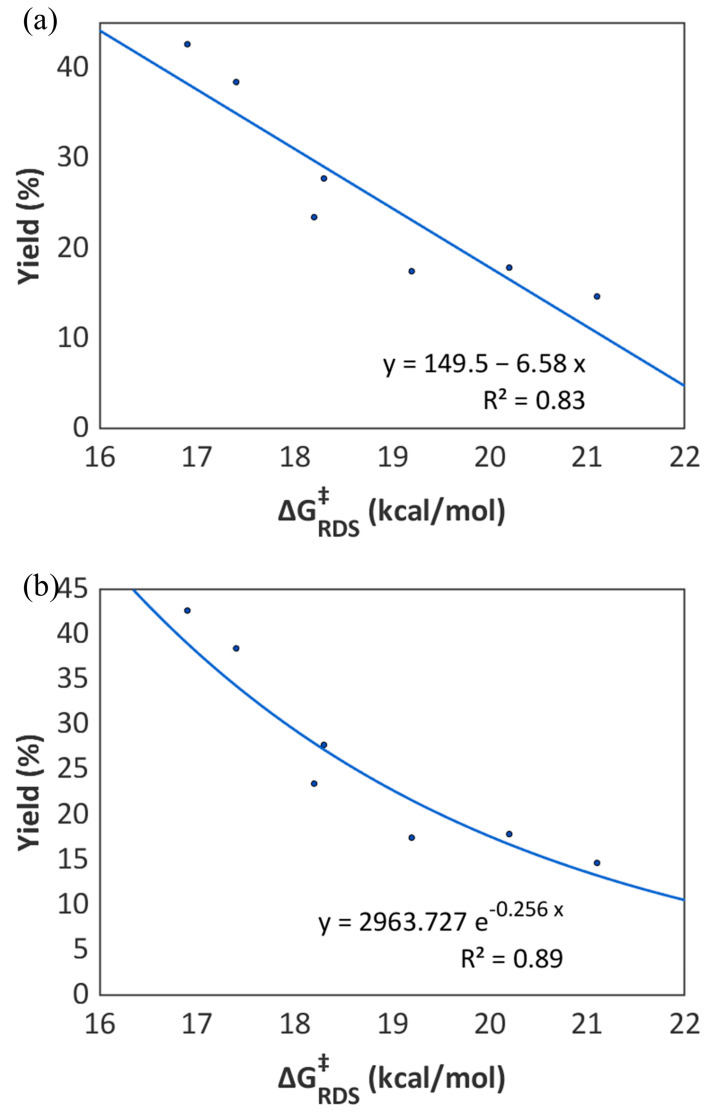
Regression analysis of the relationship between calculated ΔG^‡^_RDS_ and experimental yield of **2a** with the 7 organic bases. (**a**) Linear regression; (**b**) exponential regression.

To further validate the reliability of our treatment for calculating ΔG^‡^_RDS_, we established correlations between the calculated ΔG^‡^_RDS_ and the experimental yields. As shown in Figure 8, both linear and exponential fittings exhibit strong correlations (R^2^ = 0.83 for linear regression; R^2^ = 0.89 for exponential regression). The nearly linear decrease in yield with increasing ΔG^‡^_RDS_ highlights the expected kinetic control of the reaction, as higher activation barriers naturally lead to lower product yields under identical reaction conditions. The better fit of the exponential regression is particularly noteworthy, as it is consistent with the Arrhenius-type dependence of reaction rates on activation free energy. In mathematics, the first-order term of the Taylor expansion of an exponential function provides its linear approximation; accordingly, the linear regression between ΔG^‡^_RDS_ and the product yields also exhibits a strong correlation. The strong correlations not only confirm the effectiveness of our treatment method but also support the reliability of our proposed role of organic bases.

## 3. Materials and Methods

### 3.1. General Methods

Unless otherwise noted, all reagents were purchased from commercial suppliers and used without further purification. Reactions were monitored by thin-layer chromatography (TLC) with Yantai GF 254 silica gel plates (Yantai dexin biotechnology Co., Ltd., Yantai, China) using UV light and vanillic aldehyde or phosphomolybdic acid as visualizing agents.

Gas chromatography/mass spectrometry (GC-MS) was performed on an Agilent 5870 GC (Santa Clara, CA, USA, HP-5 column) with a flame ionization detector.

Proton nuclear magnetic resonance (^1^H NMR) spectra and carbon nuclear magnetic resonance (^13^C NMR) spectra were obtained on a Bruker 500 MHz and 400 MHz NMR instrument (Fällanden, Zurich, Switzerland, 400 and 101 MHz, respectively).

Flash column chromatography was performed using 200–300 mesh silica gel (Shanghai Titan Scientific Co., Ltd, Shanghai, China) at increased pressure. ^1^H NMR spectra, ^13^C NMR spectra, and ^19^F NMR spectra were, respectively, recorded on 500 MHz, 400 MHz (101 MHz), and 400 MHz (376 MHz) NMR spectrometers. Chemical shifts (δ) were expressed in ppm with TMS as the internal standard, and coupling constants (J) were reported in Hz.

### 3.2. Experimental Procedures

In the flow system, substrate **1** (0.4 mmol, 1 eq.), mesitylene (0.4 mmol, 1 eq.), and CH_3_CN (2 mL) were added to a 4 mL clear glass vial, while the organic base DBU (**B4**) (1.88 mmol, 4.7 eq.), photocatalyst Ru(bpy)_3_Cl_2_ (**C4**) (0.02 mmol, 5 mol%), and CH_3_CN (**S3**) (2 mL) were added to another 4 mL clear glass vial. Mesitylene (0.4 mmol, 1 eq.) was introduced as an internal standard. The reaction was conducted using an automated laboratory robotic system [32], with the microfluidic chip placed in a photoreactor. Samples were transferred to the sample pool of the chemical robot, and the reaction screening process started with sample injection and irradiation under white LED (4.8 W) modules (25 °C). The reaction parameters were set to a liquid flow rate of *v_l_* = 5 μL/min and a gas flow rate of *v_g_* = 0.04 mL/min. Upon completion, product yields were analyzed by GC-MS.

In the flask reaction, substrate **1** (0.2 mmol, 1 eq.), the organic base DBU (**B4**) (0.94 mmol, 4.7 eq.), photocatalyst Ru(bpy)_3_Cl_2_ (**C4**) (0.02 mmol, 5 mol%), and CH_3_CN (**S3**) (2 mL) were added to a 4 mL clear glass vial (4 mL) equipped with a magnetic stirring bar. The reaction mixture was stirred at 25 °C under an O_2_ atmosphere and blue LED (4.8 W) modules, with the reaction progress monitored by TLC. Upon completion, the mixture was filtered and concentrated under reduced pressure to yield the crude product. The crude product was then purified by flash column chromatography using a petroleum ether/ethyl acetate mixture as the eluent, yielding the target product **2** (15.3 mg, 52% yield).

### 3.3. Reaction Conditions Screening

#### 3.3.1. System

The system design, configuration, and application method of the high-throughput microfluidic robotic system are described in previous work [32].

#### 3.3.2. Machine Learning

Gaussian process regression was conducted with the GPy package on the AI studio of the Fei Jiang platform (https://www.paddlepaddle.org.cn/ (accessed on 18 March 2025)).

#### 3.3.3. Screening of Discrete Variables

A standardized screening protocol for photocatalytic redox reactions was established, incorporating three key parameters: five solvents (**S1**–**S5**), seven photocatalysts (**C1**–**C7**), and seven bases (**B1**–**B7**). The selected reagents are commonly used and readily accessible in photocatalytic redox studies.

In the flow system, substrate **1a** (0.2 mmol, 1 eq.), mesitylene (0.4 mmol, 1 eq.), and different solvents (2 mL) were added to a 4 mL clear glass vial, while various organic bases (0.4 mmol, 2 eq.), different photocatalysts (0.005 mmol, 2.5 mol%), and solvents (2 mL) were added to another 4 mL clear glass vial. Samples were transferred to the sample pool of the chemical robot, and the reaction screening process started with sample injection and irradiation under white LED modules (15 °C). The reaction parameters were set to a liquid flow rate of *v_l_* = 20 μL/min and a gas flow rate of *v_g_* = 0.04 mL/min. Upon completion, product yields were analyzed by GC-MS, and heat maps were generated for comparison to determine the initial optimization conditions.

#### 3.3.4. Screening of Continuous Variables

Procedure: Substrate **1a** (0.2 mmol, 1 eq.), mesitylene (0.2 mmol, 1 eq.), and CH_3_CN (2 mL) were added to a 4 mL clear glass vial, while DBU (2, 4, or 6 eq.), Ru(bpy)_3_Cl_2_ (2.5, 5 or 10 mol%), and CH_3_CN (2 mL) were added to another 4 mL clear glass vial. Nine different samples were prepared in this process. Then, these 9 samples were transferred to the sample pool of the chemical robot, and the reaction screening process started with sample injection and irradiation under white LED (4.8 W) modules (15 °C). The reaction parameters were set to a liquid flow rate of *v_l_* = 20 μL/min and a gas flow rate of *v_g_* = 0.04 mL/min. In this experiment, 9 reactions with 2 variables were conducted, and the results are shown in Table S1.

Procedure: Substrate **1a** (0.2 mmol, 1 eq.), mesitylene (0.2 mmol, 1 eq.), and CH_3_CN (2 mL) were added to a 4 mL clear glass vial, while DBU (4 eq.), Ru(bpy)_3_Cl_2_ (5 mol%), and CH_3_CN (2 mL) were added to another 4 mL clear glass vial. Two different samples were prepared in this process. Then, these 2 samples were transferred to the sample pool of the chemical robot, and the reaction screening process started with sample injection and irradiation under white LED (4.8 W) modules (15 °C). The reaction parameters were set to a liquid flow rate of *v_l_* = 5, 10, or 20 μL/min and a gas flow rate of *v_g_* = 0.04, 0.06, or 0.08 mL/min. In this experiment, 9 reactions with 2 variables were conducted, and the results are shown in Table S2.

Procedure: Substrate **1a** (0.1, 0.2, or 0.4 mmol, 1 eq.), mesitylene (0.1, 0.2, or 0.4 mmol, 1 eq.), and CH_3_CN (2 mL) were added to a 4 mL clear glass vial, while DBU (4.7 eq.), Ru(bpy)_3_Cl_2_ (5 mol%), and CH_3_CN (2 mL) were added to another 4 mL clear glass vial. Six different samples were prepared in this process. Then, these 6 samples were transferred to the sample pool of the chemical robot, and the reaction screening process started with sample injection and irradiation under white LED (4.8 W) modules (5, 15, or 25 °C). The reaction parameters were set to a liquid flow rate of *v_l_* = 5 μL/min and a gas flow rate of *v_g_* = 0.04 mL/min. In this experiment, 9 reactions with 2 variables were conducted, and the results are shown in Table S3.

### 3.4. Computational Details

All density functional theory (DFT) calculations were performed using the Gaussian 16 program package [41]. Geometry optimization was performed with M06-2X [42]-D3 [43] and def2-SVP [44] basis set for all atoms. Frequency analysis was conducted at the same level of theory to verify the stationary points to be energy minimum to obtain the thermal energy corrections. Single point energies were calculated with M06-2X-D3 and def2-TZVP [45] for all atoms. Solvent effect was calculated by using SMD solvation model (acetonitrile) [46]. The relative energies with ZPE corrections and free energies are in kcal/mol.

## 4. Conclusions

With the aid of our in-house high-throughput microfluidic system to optimize the reaction conditions, we successfully achieved the photocatalytic synthesis of 3,4-dihydroquinolone from tetrahydroquinoline. This photocatalytic protocol features mild conditions and short reaction time and demonstrates good tolerance toward diverse functional groups, including halogens, alkyl, methoxy, and hydroxy substituents, enabling efficient access to a wide range of 3,4-dihydroquinolone derivatives from readily available tetrahydroquinoline precursors. DFT calculations further elucidated the role of the organic base, which is first oxidized to a base^•+^ that subsequently undergoes a HAT process to yield the final product. This work demonstrates the capability of our system in optimizing photochemical reactions and suggests its potential for broader applications in photocatalysis.

## Figures and Tables

**Table 1:** Calculated free energies (kcal/mol) and experimental yields of product **2a** using 7 different organic bases.

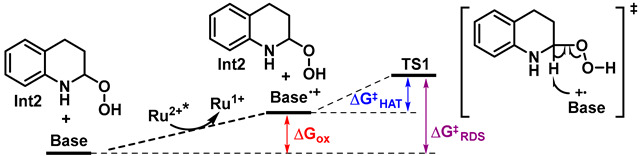				
Bases	ΔG_ox._	ΔG^0^_HAT_	ΔG^‡^_RDS_	Yield (2a) ^1^
B1	−1.5	21.1	21.1	14.7%
B2	−2.0	20.2	20.2	17.9%
B3	4.2	15.0	19.2	17.5%
B4	11.6	5.3	16.9	42.7%
B5	−3.0	18.3	18.3	27.8%
B6	13.4	4.0	17.4	38.5%
B7	7.2	11.0	18.2	23.5%

^1^ Reaction conditions: **1a** (0.10 mmol), Ru(bpy)_3_Cl_2_ (2.5 mol%), DBU (2 eq.) in MeCN (2.0 mL) at 15 °C for 5 min.

## Data Availability

The original contributions presented in this study are included in the article/Supplementary Materials. Further inquiries can be directed to the corresponding authors.

## Supplementary Materials

The following supporting information can be downloaded at: https://www.mdpi.com/article/10.3390/molecules31010026/s1, Figure S1: Batch reaction setup; Figure S2: The major structure of the high-throughput microfluidic system; Figure S3: Correlation between the relative concentration and the relative signal intensity detected by GC-MS; Table S1: Screening of the amount of catalyst and base; Table S2: Screening of carrier gas and carrier liquid flow rate; Table S3: Screening of the temperature and concentration; ^1^H, ^13^C NMR spectra data of compounds **2a**-**2q**. References [47,48,49,50,51,52] are cited in the Supplementary Materials.
