# Supplementary material for: Photocatalytic Synthesis of 3,4-Dihydroquinolone from Tetrahydroquinolines by a High-Throughput Microfluidic System and Insights into the Role of Organic Bases
Source: Molecules. 2025 Dec 22;31(1):26. doi: 10.3390/molecules31010026 (PMC12786552; doi:10.3390/molecules31010026)
Supplement: Supplementary file 1 [file molecules-31-00026-s001.zip › Manuscript-202511-SI-Eng-ver 1.5.0.pdf]

Supporting Information

Photocatalytic Synthesis of 3,4-Dihydroquinolone from Tetrahydroquinolines by a High-Throughput Microfluidic System and Insights into the Role of Organic Base

Shuyuan Ding,[a] **Tian-Yu Sun**,[a] **Heming Jiang**,[a,b] **Yun-Dong Wu**,*[a,b,c] **Xinhao Zhang***[a,b]

[a] Key Laboratory of Computational Chemistry and Drug Design, State Key Laboratory of Chemical Oncogenomics, Shenzhen Key Laboratory of Chemical Genomics, School of Chemical Biology and Biotechnology, Peking University Shenzhen Graduate School, Shenzhen 518055, China

[b] Institute of Chemical Biology, Shenzhen Bay Laboratory, Shenzhen, 518132, China

[c] College of Chemistry and Molecular Engineering, Peking University, Beijing 100871, China

* Correspondence: author. Email: wuyd@pkusz.edu.cn; zhangxh@pkusz.edu.cn

Table of Contents

1. General methods S1
2. Experimental procedures S2
3. Reaction conditions screening S4
    3.1 System S4
    3.2 Machine learning S5
    3.3 Screening of discrete variables S5
    3.4 Screening of continuous variables S6
4. Characterization data of the products S10
5. Computational details S14
6. References S15
7. NMR of Products S17
8. DFT calculated cartesian coordinates S35

1. General methods

Unless otherwise noted, all reagents were purchased from commercial suppliers and used without further purification. Reactions were monitored by thin-layer chromatography (TLC) with Yantai GF 254 silica gel plates (Yantai dexin biotechnology Co Ltd, Yantai, China) using UV light and vanillic aldehyde or phosphomolybdic acid as visualizing agents.

Gas chromatography/mass spectrometry (GC-MS) was performed on an Agilent 5870 GC (Santa Clara, CA, USA, HP-5 column) with a flame ionization detector.

Proton nuclear magnetic resonance (^{1}H NMR) spectra and carbon nuclear magnetic resonance (^{13}C NMR) spectra were obtained on a Bruker 500 MHz and 400 MHz NMR instrument (Fällanden, Zurich, CH, 400 and 101 MHz, respectively).

Flash column chromatography was performed using 200-300 mesh silica gel (Shanghai Titan Scientific Co., Ltd, Shanghai, China) at increased pressure. ^{1}H NMR spectra, ^{13}C NMR spectra and ^{19}F NMR spectra were respectively recorded on 500 MHz, 400 MHz (101 MHz) and 400 MHz (376 MHz) NMR spectrometers. Chemical shifts (δ) were expressed in ppm with TMS as the internal standard, and coupling constants (J) were reported in Hz.

2. Experimental procedures

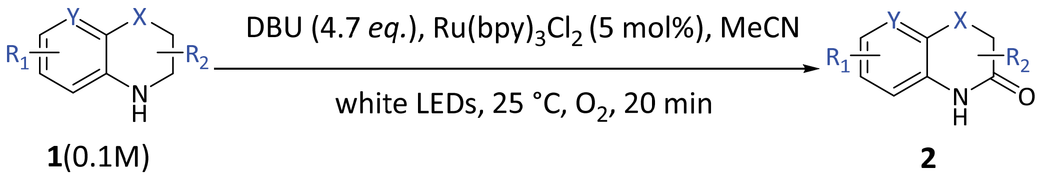

In the flow system, substrate **1** (0.4 mmol, 1 eq.), mesitylene (0.4 mmol, 1 eq.) and CH_3CN (2 mL) were added to a 4 mL clear glass vial, while organic base DBU (**B4**) (1.88 mmol, 4.7 eq.), photocatalyst Ru(bpy)$_3$Cl$_2$ (**C4**) (0.02 mmol, 5 mol%), and CH_3CN (**S3**) (2 mL) were added to another 4 mL clear glass vial. Mesitylene (0.4 mmol, 1 eq.) was introduced as an internal standard. The reaction was conducted using an automated laboratory robotic system [1], with the microfluidic chip placed in a photoreactor. Samples are transferred to the sample pool of the chemical robot, and reaction screening process started with sample injection and irradiation under white LED (4.8 W) modules (25 °C). The reaction parameters were set to a liquid flow rate of v_l= 5 μL/min and a gas flow rate of v_g= 0.04 mL/min. Upon completion, product yields were analyzed by GC-MS.

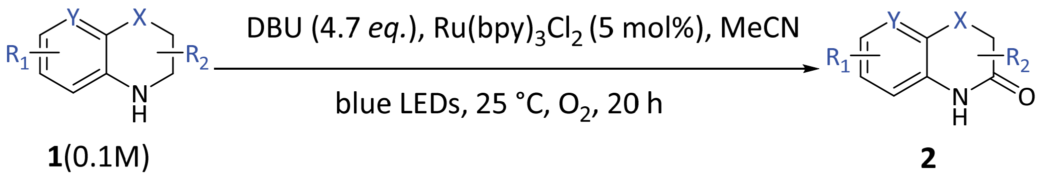

In the flask reaction, substrate **1** (0.2 mmol, 1 eq.), organic base DBU (**B4**) (0.94 mmol, 4.7 eq.), photocatalyst Ru(bpy)$_3$Cl$_2$ (**C4**) (0.02 mmol, 5 mol%), and CH_3CN (**S3**) (2 mL) were added to a 4 mL clear glass vial (4 mL) equipped with a magnetic stirring bar. The reaction mixture was stirred at 25°C under an O_2 atmosphere and blue LED (4.8 W) modules, with the reaction progress monitored by TLC. Upon completion, the mixture was filtered and concentrated under reduced pressure to yield the crude product. The crude product was then purified by flash column chromatography using a petroleum ether/ethyl acetate mixture as the eluent, affording the target product **2** (15.3 mg, 52% yield).

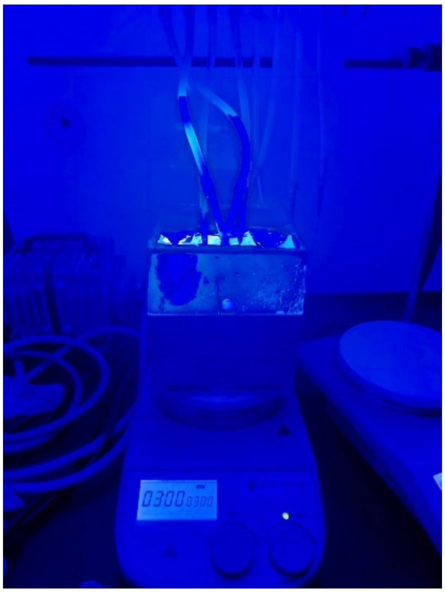

Figure S1. Batch reaction setup

3. Reaction conditions screening

3.1 System

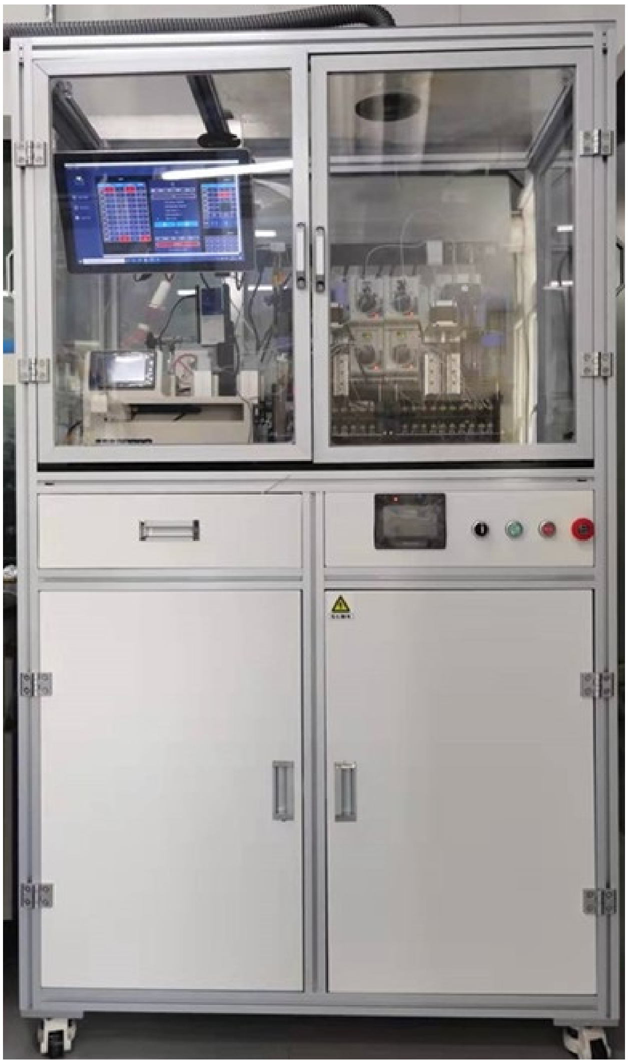

Figure S2. The major structure of the high-throughput microfluidic system.

The system design, configuration and application method of high-throughput microfluidic robotic system are shown in previous work. [1]

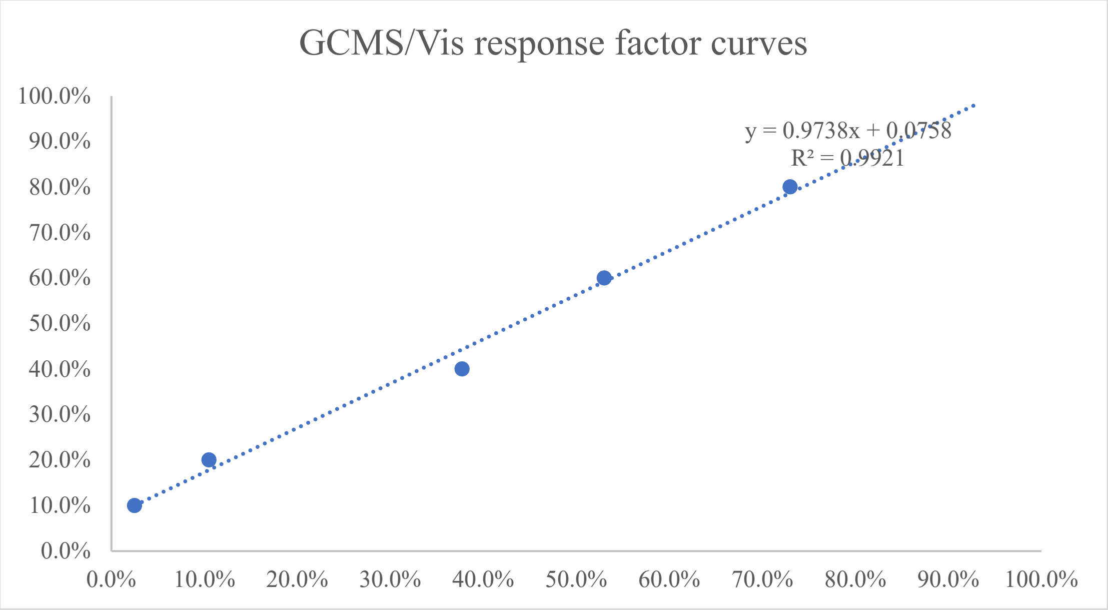

Figure S3. Correlation between the relative concentration (yield (**2a**)/IS) of reaction mixture and the relative signal intensity detected by GC-MS.

3.2 Machine learning

Gaussian process regression was conducted with the GPy package on the AI studio of the Fei Jiang platform (https:// www.paddlepaddle.org.cn/).

3.3 Screening of discrete variables

A standardized screening protocol for photocatalytic redox reactions was established, incorporating three key parameters: five solvents (**S1–S5**), seven photocatalysts (**C1–C7**), and seven bases (**B1–B7**). The selected reagents are commonly used and readily accessible in photocatalytic redox studies.

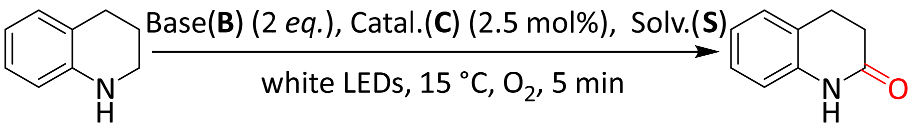

In the flow system, substrate **1a** (0.2 mmol, 1 eq.), mesitylene (0.4 mmol, 1 eq.) and different solvents (2 mL) were added to a 4 mL clear glass vial, while various organic base (0.4 mmol, 2 eq.), different photocatalyst (0.005 mmol, 2.5 mol%), and solvent (2 mL) were added to another 4 mL clear glass vial. Samples are transferred to the sample pool of the chemical robot, and reaction screening process started with sample injection and irradiation under white LED modules (15 °C). The reaction parameters were set to a liquid flow rate of v_l= 20 µL/min and a gas flow rate of v_g= 0.04 mL/min. Upon completion, product yields were analyzed by GC-MS, and heat maps were generated for comparison to determine the initial optimization conditions.

3.4 Screening of continuous variables

Table S1. Screening of the amount of catalyst and base

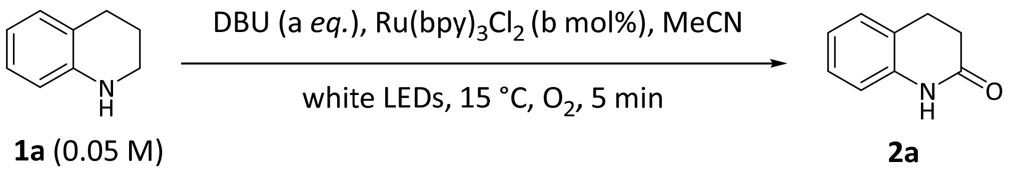

Entry	Cat. mol%	Base *eq.*	v_l μL·min^{-1}	v_g SCCM	T °C	c mol·L^{-1}	Yield %
1	2.5	2	20	0.04	15	0.05	38.8
2	2.5	4	20	0.04	15	0.05	40.7
3	2.5	6	20	0.04	15	0.05	41.7
4	5	2	20	0.04	15	0.05	38.2
5	5	4	20	0.04	15	0.05	41.7
6	5	6	20	0.04	15	0.05	41.3
7	10	2	20	0.04	15	0.05	37.3
8	10	4	20	0.04	15	0.05	40.7
9	10	6	20	0.04	15	0.05	42.1

Procedure: substrate **1a** (0.2 mmol, 1 eq.), mesitylene (0.2 mmol, 1 eq.) and CH_3CN (2 mL) were added to a 4 mL clear glass vial, while DBU (2, 4 or 6 eq.), $Ru(bpy)_3Cl_2$ (2.5, 5 or 10 mol%), and CH_3CN (2 mL) were added to another 4 mL clear glass vial. 9 different samples were prepared in this process. Then these 9 samples are transferred to the sample pool of the chemical robot, and reaction screening process started with sample injection and irradiation under white LED (4.8 W) modules (15 °C). The reaction parameters were set to a liquid flow rate of v_l= 20 μL/min and a gas flow rate of v_g= 0.04 mL/min. In this experiments, 9 reactions with 2 variables are conducted, and the results are shown in **Table S1**.

Table S2. Screening of carrier gas and carrier liquid flow rate

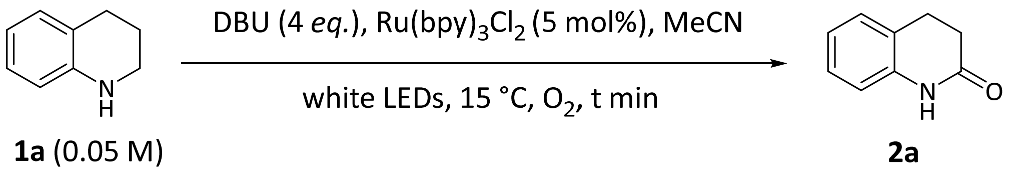

Entry	Cat. mol%	Base *eq.*	v_l μL·min^{-1}	v_g SCCM	T °C	c mol·L^{-1}	Yield %
1	5	4	5	0.04	15	0.05	57.4

2	5	4	5	0.06	15	0.05	54.9
3	5	4	5	0.08	15	0.05	60.0
4	5	4	10	0.04	15	0.05	53.6
5	5	4	10	0.06	15	0.05	52.3
6	5	4	10	0.08	15	0.05	49.9
7	5	4	20	0.04	15	0.05	30.7
8	5	4	20	0.06	15	0.05	31.9
9	5	4	20	0.08	15	0.05	30.1

Procedure: substrate **1a** (0.2 mmol, 1 eq.), mesitylene (0.2 mmol, 1 eq.) and CH_3CN (2 mL) were added to a 4 mL clear glass vial, while DBU (4 eq.), $Ru(bpy)_3Cl_2$ (5 mol%), and CH_3CN (2 mL) were added to another 4 mL clear glass vial. 2 different samples were prepared in this process. Then these 2 samples are transferred to the sample pool of the chemical robot, and reaction screening process started with sample injection and irradiation under white LED (4.8 W) modules(15 °C). The reaction parameters were set to a liquid flow rate of v_l= 5, 10 or 20 μL/min and a gas flow rate of v_g= 0.04, 0.06, 0.08 mL/min. In this experiments, 9 reactions with 2 variables are conducted, and the results are shown in **Table S2**.

Table S3. Screening of the temperature and concentration

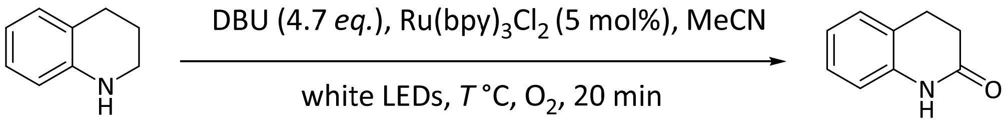

1a (c M) **2a**

Entry	Cat. mol%	Base *eq.*	v_l μL·min^{-1}	v_g SCCM	T °C	c mol·L^{-1}	Yield %
1	5	4.7	5	0.04	5	0.025	39.8
2	5	4.7	5	0.04	5	0.05	51.1
3	5	4.7	5	0.04	5	0.1	53.1
4	5	4.7	5	0.04	15	0.025	59.1
5	5	4.7	5	0.04	15	0.05	57.7
6	5	4.7	5	0.04	15	0.1	69.7
7	5	4.7	5	0.04	25	0.025	64.6
8	5	4.7	5	0.04	25	0.05	63.7
9	5	4.7	5	0.04	25	0.1	76.1

Procedure: substrate **1a** (0.1, 0.2 or 0.4 mmol, 1 eq.), mesitylene (0.1, 0.2 or 0.4 mmol, 1 eq.) and CH_3CN (2 mL) were added to a 4 mL clear glass vial, while DBU (4.7 eq.), $Ru(bpy)_3Cl_2$ (5 mol%), and CH_3CN (2 mL) were added to another 4 mL clear glass vial. 6 different samples were prepared in this process. Then these 6 samples are transferred to the sample pool of the chemical robot, and reaction screening process started with sample injection and irradiation under white LED (4.8 W) modules(5, 15 or 25 °C). The reaction parameters were set to a liquid flow rate of v_l= 5 μL/min and a gas flow rate of v_g= 0.04 mL/min. In this experiments, 9 reactions with 2 variables are conducted, and the results are shown in **Table S3**.

3.5 Substrate Scope

Good product performance has been mentioned in the article. Here is the performance of electron-withdrawing substituent. At the **C7** position, 7-amine (**2r**) group was not reactive under the reaction conditions, likely due to oxidation of the amino group to a nitro group, and the presence of a strong electron-withdrawing 7-nitro substituent (**2s**) fully suppressed the reaction. We are also continuing to investigate reaction conditions of 7-nitro substituent (**2s**). At the **C6** position, cyano (**2t**) and nitro (**2u**) led to reduced reactivity, affording the products in 34% and 5% yields, respectively. This also indicates that electron-withdrawing groups are unfavorable to the reaction. 2,3-dihydroquinolin-4(1H)-one (**1v**) was completely inactive, further highlighting the negatively effect of electron-withdrawing group. The N-methyl tetrahydroquinoline (**1q**) was well tolerated (up to 88% yield), whereas N-Boc tetrahydroquinoline (**1w**) was completely unreactive, likely due to the electron-withdrawing Boc group reducing the reactivity. Furthermore, several heterocyclic substrates such as 3,4-dihydro-2H-benzo[b][1,4]oxa-zine (**1aa**), 1,2,3,4-tetrahydro-1,5-naphthyridine (**1ab**) and 2,3,4,5-tetrahydro-1H-benzo[b]azepine (**1ac**) were also evaluated, affording yield of 33%, 18% and 0%, respectively. Overall, this comprehensive substrate evaluation demonstrates the robustness of the photocatalytic method.

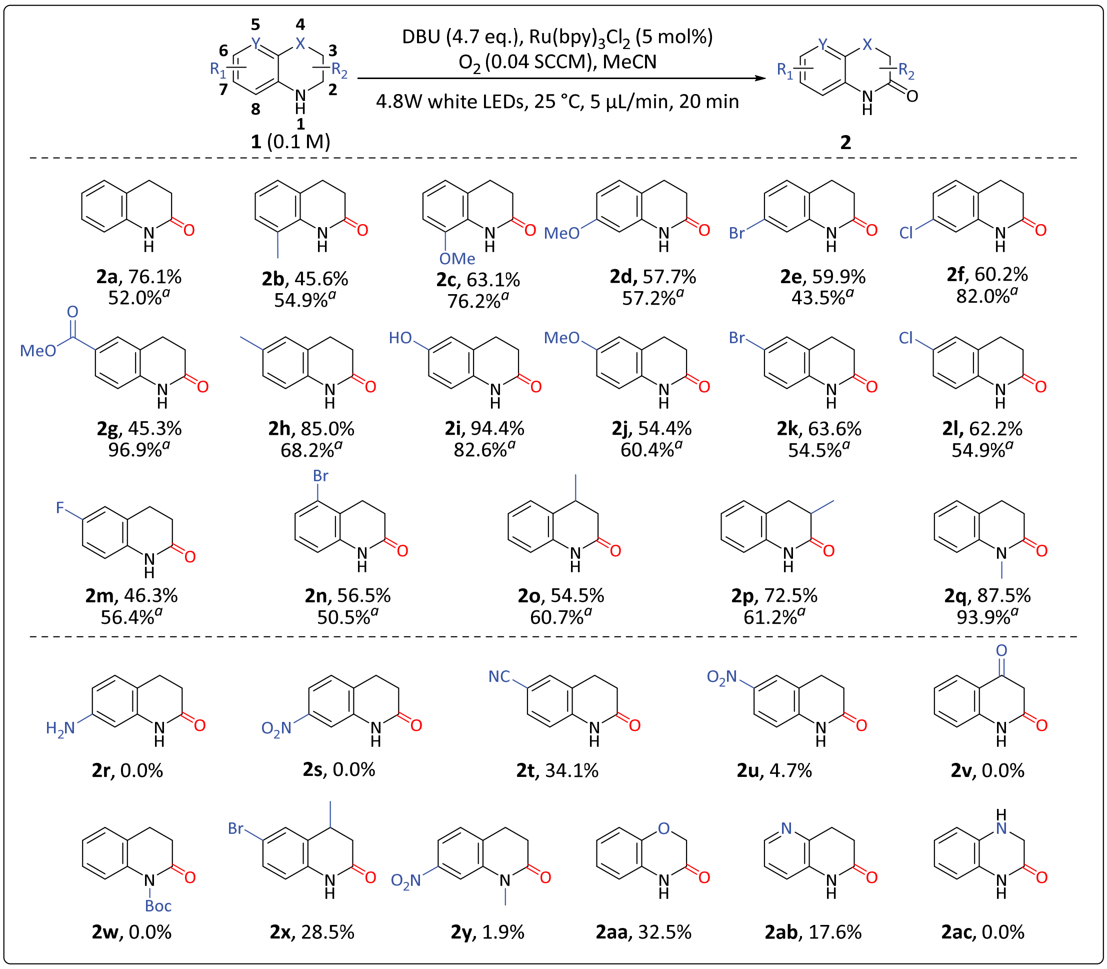
DBU (4.7 eq.), Ru(bpy)3Cl2 (5 mol%)
O2 (0.04 SCCM), MeCN
4.8W white LEDs, 25 °C, 5 µL/min, 20 min
1 (0.1 M)
2
2a, 76.1%
52.0%a
2b, 45.6%
54.9%a
2c, 63.1%
76.2%a
2d, 57.7%
57.2%a
2e, 59.9%
43.5%a
2f, 60.2%
82.0%a
2g, 45.3%
96.9%a
2h, 85.0%
68.2%a
2i, 94.4%
82.6%a
2j, 54.4%
60.4%a
2k, 63.6%
54.5%a
2l, 62.2%
54.9%a
2m, 46.3%
56.4%a
2n, 56.5%
50.5%a
2o, 54.5%
60.7%a
2p, 72.5%
61.2%a
2q, 87.5%
93.9%a
2r, 0.0%
2s, 0.0%
2t, 34.1%
2u, 4.7%
2v, 0.0%
2w, 0.0%
2x, 28.5%
2y, 1.9%
2aa, 32.5%
2ab, 17.6%
2ac, 0.0%

4. Characterization data of the products

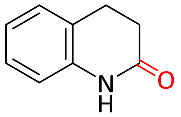

3,4-dihydro-2-quinolone (2a) [2]: R_f = 0.25 (Petroleum ether/EtOAc, 2:1). 15.3 mg, 52% yield. White solid. ^{1}H NMR (500 MHz, Chloroform-*d*) δ 9.06 (s, 1H), 7.19 – 7.11 (m, 2H), 7.01 – 6.96 (t, *J* = 7.5 Hz, 1H), 6.84 (d, *J* = 6.7 Hz, 1H), 2.99 – 2.94 (m, 2H), 2.67 – 2.62 (m, 2H). ^{13}C NMR (101 MHz, Chloroform-*d*) δ 172.1, 137.3, 127.9, 127.5, 123.6, 123.0, 115.5, 30.7, 25.3.

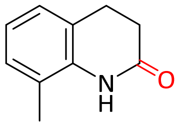

8-methyl-3,4-dihydro-2-quinolone (2b) [3]: R_f = 0.25 (Petroleum ether/EtOAc, 2:1). 17.7 mg, 55% yield. Light yellow solid. ^{1}H NMR (500 MHz, Chloroform-*d*) δ 7.98 (s, 1H), 7.02 (t, *J* = 7.3 Hz, 2H), 6.90 (t, *J* = 7.5 Hz, 1H), 3.00 – 2.88 (m, 2H), 2.66 – 2.56 (m, 2H), 2.26 (s, 3H). ^{13}C NMR (101 MHz, Chloroform-*d*) δ 171.7, 135.7, 129.1, 125.8, 123.9, 123.0, 122.7, 30.9, 25.8, 16.9.

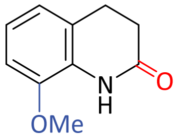

8-methoxy-3,4-dihydro-2-quinolone (2c) [4]: R_f= 0.25 (Petroleum ether/EtOAc, 2:1). 27.0 mg, 76% yield. Light yellow solid. ^{1}H NMR (500 MHz, Chloroform-*d*) δ 7.83 (s, 1H), 6.96 (t, *J* = 7.9 Hz, 1H), 6.78 - 6.80 (m, 2H), 3.89 (s, 3H), 2.98 (t, *J* = 7.5 Hz, 2H), 2.68 – 2.61 (m, 2H). ^{13}C NMR (101 MHz, Chloroform-*d*) δ 170.3, 145.8, 126.5, 124.0, 122.7, 119.9, 109.0, 55.7, 30.6, 25.3.

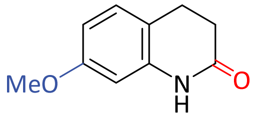

7-methoxy-3,4-dihydro-2-quinolone (2d) [4]: R_f = 0.25 (Petroleum ether/EtOAc, 2:1). 20.3 mg, 57% yield. Light yellow solid. ^{1}H NMR (500 MHz, Chloroform-*d*) δ 8.79 (s, 1H), 7.05 (d, *J* = 8.3 Hz, 1H), 6.53 (dd, *J* = 8.3, 2.5 Hz, 1H), 6.39 (d, *J* = 2.5 Hz, 1H), 3.78 (s, 3H), 2.90 (t, *J* = 7.6 Hz, 2H), 2.62 (t, *J* = 8.0 Hz, 2H). ^{13}C NMR (101 MHz, Chloroform-*d*) δ 172.4, 159.2, 138.3, 128.5, 115.7, 108.3, 101.7, 55.4, 31.0, 24.5.

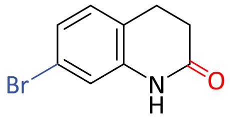

7-bromo-3,4-dihydro-2-quinolone (2e) [5]: R_f = 0.25 (Petroleum ether/EtOAc, 2:1). 19.7mg, 44% yield. Light yellow solid. ^{1}H NMR (500 MHz, Chloroform-*d*) δ 9.14 (s, 1H), 7.10 (dd, *J* = 8.0, 1.9 Hz, 1H), 7.04 – 6.97 (m, 2H), 2.92 (t, *J* = 7.6 Hz, 2H), 2.62 – 2.66 (m, 2H). ^{13}C NMR (101 MHz, Chloroform-*d*) δ 171.7, 138.6, 129.3, 125.9, 122.5, 120.7, 118.3, 30.4, 24.9.

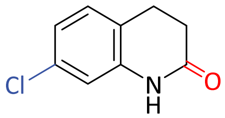

7-chloro-3,4-dihydro-2-quinolone (2f) [5]: $R_f = 0.25$ (Petroleum ether/EtOAc, 2:1). 29.8 mg, 82% yield. White solid. ^{1}H NMR (500 MHz, Chloroform-*d*) δ 9.26 (s, 1H), 7.10 (d, *J* = 8.1 Hz, 1H), 6.97 (dd, *J* = 8.1, 2.0 Hz, 1H), 6.88 (d, *J* = 2.0 Hz, 1H), 2.96 (t, *J* = 7.5 Hz, 2H), 2.69 – 2.65 (m, 2H). ^{13}C NMR (101 MHz, Chloroform-*d*) δ 172.0, 138.4, 133.0, 128.9, 122.9, 122.0, 115.5, 30.5, 24.8.

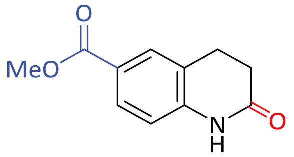

6-methylformate-3,4-dihydro-2-quinolone (2g) [6]: $R_f = 0.25$ (Petroleum ether/EtOAc, 2:1). 39.8 mg, 97% yield. White solid. ^{1}H NMR (500 MHz, Chloroform-*d*) δ 8.89 (s, 1H), 7.89 – 7.86 (m, 2H), 6.85 (d, *J* = 8.7 Hz, 1H), 3.90 (s, 3H), 3.03 (t, *J* = 7.6 Hz, 2H), 2.70 – 2.66 (m, 2H). ^{13}C NMR (101 MHz, Chloroform-*d*) δ 171.8, 166.5, 141.3, 129.6, 129.5, 124.9, 123.3, 115.1, 52.0, 30.4, 25.1.

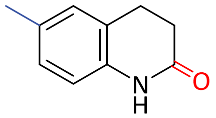

6-methyl-3,4-dihydro-2-quinolone (2h) [3]: $R_f = 0.25$ (Petroleum ether/EtOAc, 2:1). 22.0 mg, 68% yield. Light yellow solid. ^{1}H NMR (500 MHz, Chloroform-*d*) δ 8.59 (s, 1H), 6.97 (d, *J* = 4.2 Hz, 2H), 6.70 (d, *J* = 8.4 Hz, 1H), 2.94 (t, *J* = 7.4 Hz, 2H), 2.61 (t, *J* = 7.5 Hz, 2H), 2.29 (s, 3H). ^{13}C NMR (101 MHz, Chloroform-*d*) δ 171.8, 134.9, 132.6, 128.6, 127.9, 123.6, 115.3, 30.8, 25.4, 20.7.

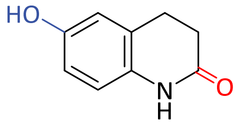

6-hydroxy-3,4-dihydroquinolin-2(1H)-one (2i) [7]: $R_f = 0.25$ (CH_2Cl_2/MeOH = 30:1). 27.0 mg, 83%; white solid. ^{1}H NMR (500 MHz, DMSO-*d*$_6$) δ 9.79 (s, 1H), 9.02 (s, 1H), 6.67 (d, *J* = 8.4 Hz, 1H), 6.62 – 6.51 (m, 2H), 2.77 (t, *J* = 7.5 Hz, 2H), 2.38 (dd, *J* = 8.5, 6.6 Hz, 2H). ^{13}C NMR (101 MHz, DMSO-*d*$_6$) δ 170.2, 152.8, 130.7, 125.2, 116.3, 115.0, 113.9, 30.9, 25.5.

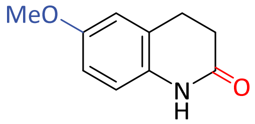

6-methoxy-3,4-dihydroquinolin-2(1H)-one (2j) [3]: $R_f = 0.25$ (Petroleum ether/EtOAc, 2:1). 21.4 mg, 60% yield. White solid. ^{1}H NMR (500 MHz, Chloroform-*d*) δ 9.37 (s, 1H), 6.80 – 6.69 (m, 3H), 3.77 (s, 3H), 2.93 (dd, *J* = 8.5, 6.7 Hz, 2H), 2.60 (dd, *J* = 8.5, 6.6 Hz, 2H). ^{13}C NMR (101 MHz, Chloroform-*d*) δ 171.9, 155.6, 130.9, 124.9, 116.4, 113.8, 112.4, 55.6, 30.6, 25.7.

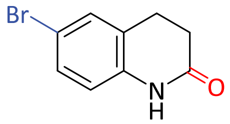

6-bromo-3,4-dihydro-2-quinolone (2k) [3]: $R_f = 0.25$ (Petroleum ether/EtOAc, 2:1). 24.7 mg, 55% yield. White solid. ^{1}H NMR (500 MHz, Chloroform-*d*) δ 9.27 (s, 1H), 7.32 – 7.28 (m, 2H),

6.75 (d, J = 8.2 Hz, 1H), 2.97 (t, J = 7.6 Hz, 2H), 2.67 – 2.63 (m, 2H). ^{13}C NMR (101 MHz, Chloroform-*d*) δ 171.8, 136.4, 130.8, 130.4, 125.6, 117.0, 115.4, 30.3, 25.1.

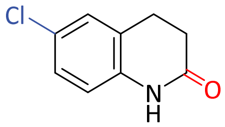

6-chloro-3,4-dihydro-2-quinolone (2l) [3]: R_f = 0.25 (Petroleum ether/EtOAc, 2:1). 20.0 mg, 55% yield. White solid. ^{1}H NMR (500 MHz, Chloroform-*d*) δ 9.31 (s, 1H), 7.13 (d, J = 7.4 Hz, 2H), 6.78 (d, J = 8.9 Hz, 1H), 2.95 (t, J = 7.6 Hz, 2H), 2.65 – 2.61 (m, 2H). ^{13}C NMR (101 MHz, Chloroform-*d*) δ 171.9, 135.9, 128.0, 127.9, 127.4, 125.2, 116.6, 30.3, 25.2.

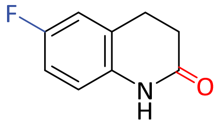

6-fluoro-3,4-dihydro-2-quinolone (2m) [3]: R_f = 0.25 (Petroleum ether/EtOAc, 2:1). 18.6 mg, 56% yield. White solid. ^{1}H NMR (500 MHz, Chloroform-*d*) δ 9.59 (s, 1H), 6.89 – 6.84 (m, 2H), 6.82 (dd, J = 8.5, 4.9 Hz, 1H), 2.95 (t, J = 7.6 Hz, 2H), 2.63 (t, J = 7.6 Hz, 2H). ^{13}C NMR (101 MHz, Chloroform-*d*) δ 172.1, 159.9, 157.5, 133.5, 133.5, 125.4, 125.3, 116.6, 116.5, 114.9, 114.7, 114.1, 113.9, 30.2, 25.4. ^{19}F NMR (376 MHz, Chloroform-*d*) δ -120.3.

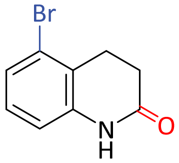

5-bromo-3,4-dihydro-2-quinolone (2n) [8]: R_f = 0.25 (Petroleum ether/EtOAc, 2:1). 22.8 mg, 51% yield. White solid. ^{1}H NMR (500 MHz, Chloroform-*d*) δ 9.01 (s, 1H), 7.23 (d, J = 8.1 Hz, 1H), 7.03 (t, J = 8.0 Hz, 1H), 6.78 (d, J = 8.0 Hz, 1H), 3.08 (t, J = 7.7 Hz, 2H), 2.68 – 2.64 (m, 2H). ^{13}C NMR (101 MHz, Chloroform-*d*) δ 171.6, 138.5, 128.6, 127.1, 123.8, 123.5, 114.7, 30.1, 25.5.

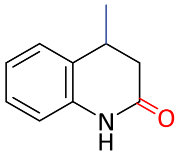

4-methylquinolin-2(1H)-one (2o) [9]: R_f = 0.25 (Petroleum ether/EtOAc, 2:1). 19.6 mg, 61% yield. White solid. ^{1}H NMR (400 MHz, Chloroform-*d*) δ 9.53 (s, 1H), 7.20 – 7.14 (m, 2H), 7.02 (t, J = 7.5 Hz, 1H), 6.88 (d, J = 7.8 Hz, 1H), 3.10 – 3.16 (m, 1H), 2.74 (dd, J = 16.1, 5.9 Hz, 1H), 2.44 (dd, J = 16.1, 7.2 Hz, 1H), 1.31 (d, J = 7.0 Hz, 3H). ^{13}C NMR (101 MHz, Chloroform-*d*) δ 171.9, 136.5, 128.7, 127.5, 126.4, 123.3, 115.8, 38.4, 30.7, 19.8.

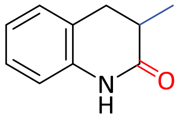

3-methyl-3,4-dihydro-2-quinolone (2p) [4]: R_f = 0.25 (Petroleum ether/EtOAc, 2:1). 19.7 mg, 61% yield. Light yellow solid. ^{1}H NMR (500 MHz, Chloroform-*d*) δ 8.54 (s, 1H), 7.16 (dd, J = 14.5, 7.0 Hz, 2H), 6.98 (td, J = 7.5, 1.2 Hz, 1H), 6.80 (d, J = 7.9 Hz, 1H), 3.00 (dd, J = 15.2, 5.6

Hz, 1H), 2.79 – 2.61 (m, 2H), 1.29 (d, J = 6.8 Hz, 3H). ^{13}C NMR (101 MHz, Chloroform-d) δ 174.5, 137.2, 128.0, 127.4, 123.6, 122.9, 115.1, 34.9, 33.4, 15.3.

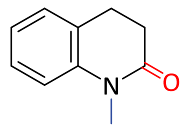

1-methyl-3,4-dihydro-2-quinolone (2q) [10]: R_f = 0.25 (Petroleum ether/EtOAc, 15:1). 30.3 mg, 94% yield. Light yellow liquid. ^{1}H NMR (500 MHz, Chloroform-d) δ 7.27 – 7.20 (m, 1H), 7.14 (d, J = 7.5 Hz, 1H), 7.02 – 6.94 (m, 2H), 3.33 (s, 3H), 2.91 – 2.84 (m, 2H), 2.65 – 2.59 (m, 2H). ^{13}C NMR (101 MHz, Chloroform-d) δ 170.3, 140.6, 127.6, 127.4, 126.1, 122.7, 114.6, 31.7, 29.4, 25.3.

5. Computational details

All density functional theory (DFT) calculations were performed using the Gaussian 16 program package. [11] Geometry optimization was performed with M06-2X [12]-D3 [13] and def2-SVP [14] basis set for all atoms. Frequency analysis was conducted at the same level of theory to verify the stationary points to be energy minimum to obtain the thermal energy corrections. Single point energies were calculated with M06-2X-D3 and def2-TZVP [15] for all atoms. Solvent effect was calculated by using SMD solvation model (acetonitrile). [16] The relative energies with ZPE corrections and free energies are in kcal/mol. Geometry optimizations were performed using the def2-SVP basis set to efficiently explore the reaction landscape, while single-point calculations were carried out with the def2-TZVP basis set.

6. References

1. Jiang, H.; Chen, Y.; Huang, M.; Liu, T.; Wu, Y.-D.; Zhang, X. Exploring New Reactions with an Accessible High-Throughput Screening (Open-HTS) Chemical Robotic System. *Org. Process Res. Dev.* **2025**, *29*, 1423–1431.
2. Wu, L.; Hao, Y.; Liu, Y.; Wang, Q. NIS-Mediated Oxidative Arene C(Sp^2)–H Amidation toward 3,4-Dihydro-2(1 *H*)-Quinolinone, Phenanthridone, and *N* -Fused Spirolactam Derivatives. *Org. Biomol. Chem.* **2019**, *17*, 6762–6770.
3. Sun, W.; Ling, C.-H.; Au, C.-M.; Yu, W.-Y. Ruthenium-Catalyzed Intramolecular Arene C(Sp^2)–H Amidation for Synthesis of 3,4-Dihydroquinolin-2(1 *H*)-Ones. *Org. Lett.* **2021**, *23*, 3310–3314.
4. Yang, L.; Shi, L.; Xing, Q.; Huang, K.-W.; Xia, C.; Li, F. Enabling CO Insertion into *o* -Nitrostyrenes beyond Reduction for Selective Access to Indolin-2-One and Dihydroquinolin-2-One Derivatives. *ACS Catal.* **2018**, *8*, 10340–10348.
5. Tan, Y.-F.; Long, C.-J.; Guan, Z.; He, Y.-H. Selective Electrochemical Oxidation of Tetrahydroquinolines to 3,4-Dihydroquinolones. *Green Chem.* **2022**, *24*, 4581–4587.
6. Fujita, K.; Takahashi, Y.; Owaki, M.; Yamamoto, K.; Yamaguchi, R. Synthesis of Five-, Six-, and Seven-Membered Ring Lactams by cp*Rh Complex-Catalyzed Oxidative N-Heterocyclization of Amino Alcohols. *Org. Lett.* **2004**, *6*, 2785–2788.
7. Xie, D.; Zhang, S. Selective Reduction of Quinolinones Promoted by a SmI_2 /H_2 O/MeOH System. *J. Org. Chem.* **2022**, *87*, 8757–8763.
8. Tian, X.; Li, X.; Duan, S.; Du, Y.; Liu, T.; Fang, Y.; Chen, W.; Zhang, H.; Li, M.; Yang, X. Room Temperature Benzofused Lactam Synthesis Enabled by Cobalt(III)-Catalyzed C(Sp^2)-H Amidation. *Adv. Synth. Catal.* **2021**, *363*, 1050–1058.
9. Linsenmeier, A.M.; Braje, W.M. Efficient One-Pot Synthesis of Dihydroquinolinones in Water at Room Temperature. *Tetrahedron* **2015**, *71*, 6913–6919.
10. Yang, X.; Wang, L.; Hu, F.; Xu, L.; Li, S.; Li, S.-S. Redox-Triggered Switchable Synthesis of 3,4-Dihydroquinolin-2(1 *H*)-One Derivatives via Hydride Transfer/ *N* -Dealkylation/ *N* -Acylation. *Org. Lett.* **2021**, *23*, 358–364.
11. Frisch, M.J.; Trucks, G.W.; Schlegel, H.B.; Scuseria, G.E.; Robb, M.A.; Cheeseman, J.R.; Scalmani, G.; Barone, V.; Petersson, G.A.; Nakatsuji, H.; et al. Gaussian 16 Rev. C.01 2016.
12. Zhao, Y.; Truhlar, D.G. The M06 Suite of Density Functionals for Main Group Thermochemistry, Thermochemical Kinetics, Noncovalent Interactions, Excited States, and Transition Elements: Two New Functionals and Systematic Testing of Four M06-Class Functionals and 12 Other Functionals. *Theor Chem Account* **2008**, *120*, 215–241.
13. Grimme, S.; Ehrlich, S.; Goerigk, L. Effect of the Damping Function in Dispersion Corrected Density Functional Theory. *J Comput Chem* **2011**, *32*, 1456–1465.
14. Weigend, F.; Ahlrichs, R. Balanced Basis Sets of Split Valence, Triple Zeta Valence and Quadruple Zeta Valence Quality for H to Rn: Design and Assessment of Accuracy. *Phys. Chem. Chem. Phys.* **2005**, *7*, 3297-3305.
15. Weigend, F. Accurate Coulomb-Fitting Basis Sets for H to Rn. *Phys. Chem. Chem. Phys.* **2006**, *8*, 1057-1065.
16. Marenich, A.V.; Cramer, C.J.; Truhlar, D.G. Universal Solvation Model Based on Solute Electron Density and on a Continuum Model of the Solvent Defined by the Bulk Dielectric

Constant and Atomic Surface Tensions. *J. Phys. Chem. B* **2009**, *113*, 6378–6396.

7. NMR of Products

^{1}H-NMR Spectrum ($CDCl_3$) of 2a

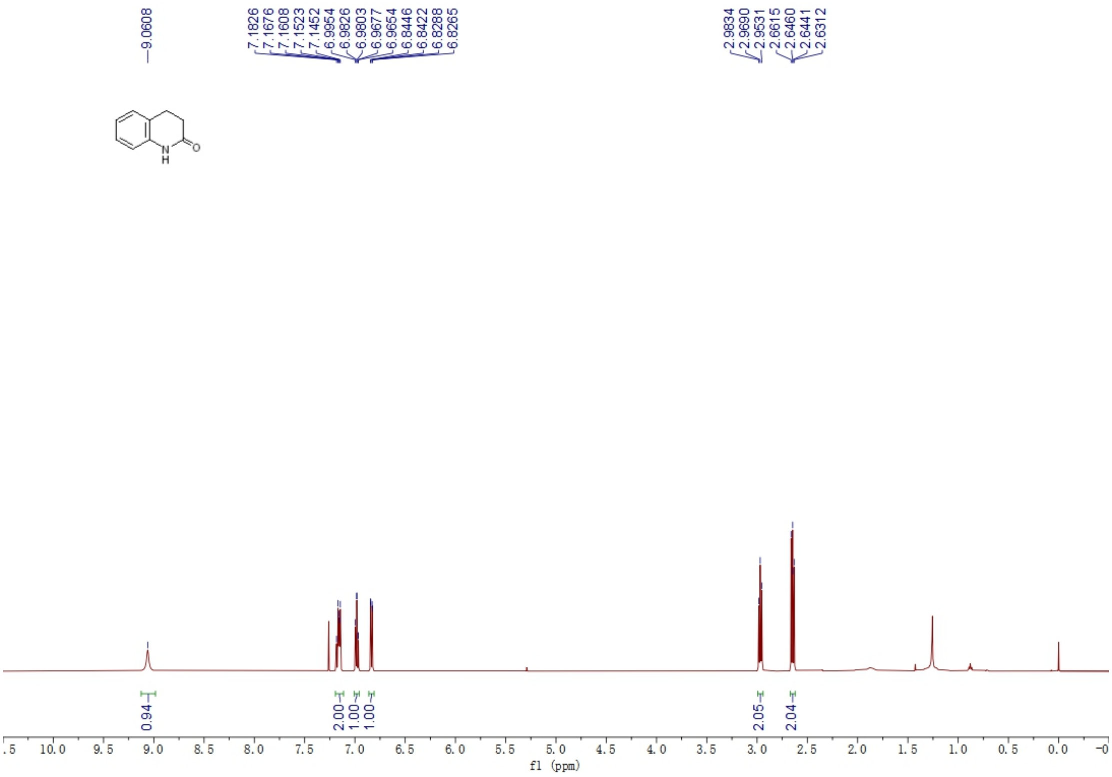

^{13}C-NMR Spectrum ($CDCl_3$) of 2a

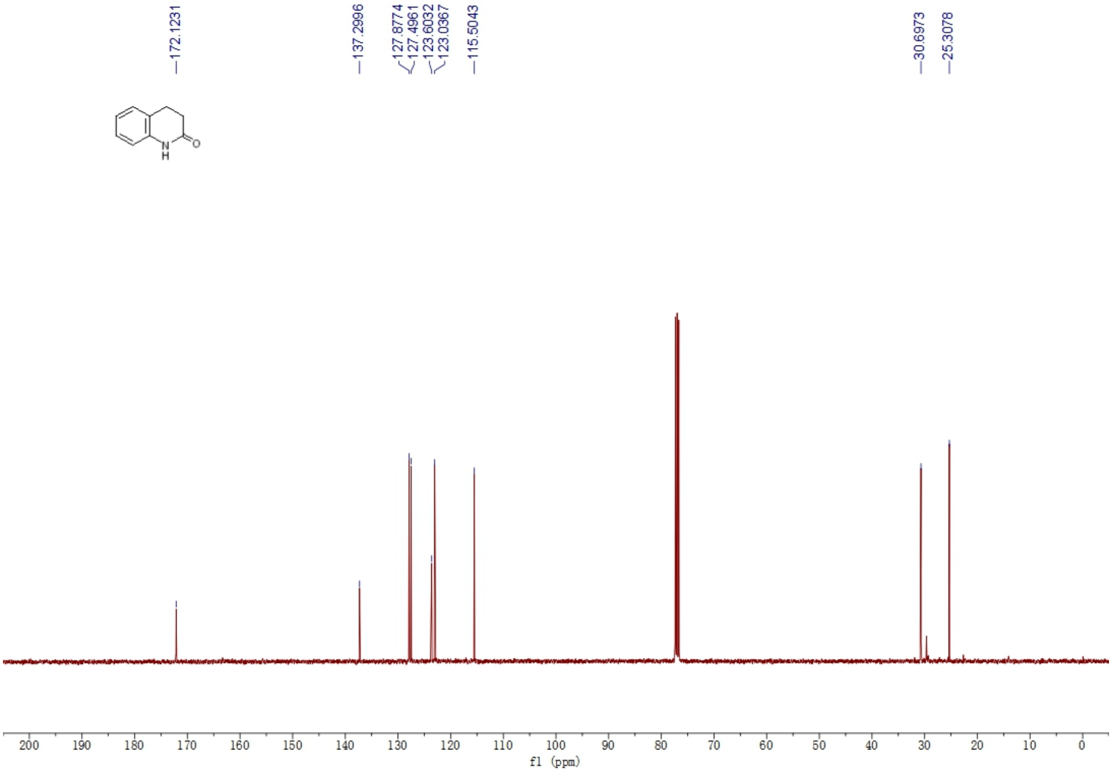

^{1}H-NMR Spectrum ($CDCl_3$) of 2b

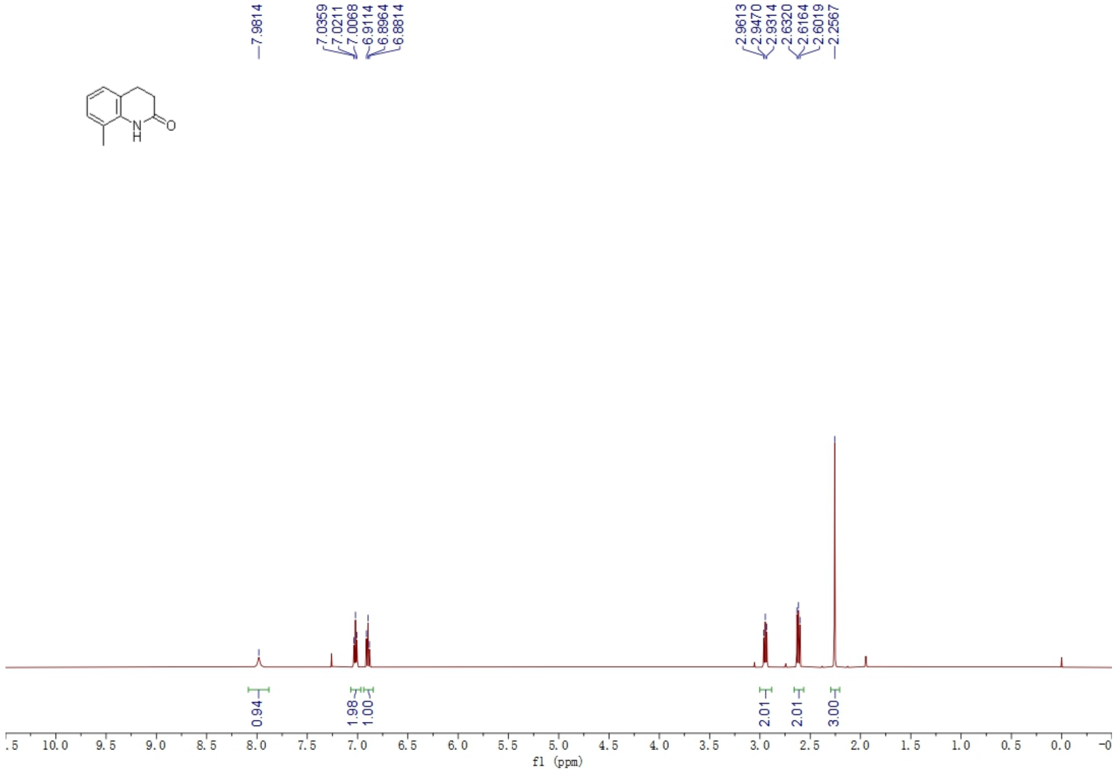

^{13}C-NMR Spectrum ($CDCl_3$) of 2b

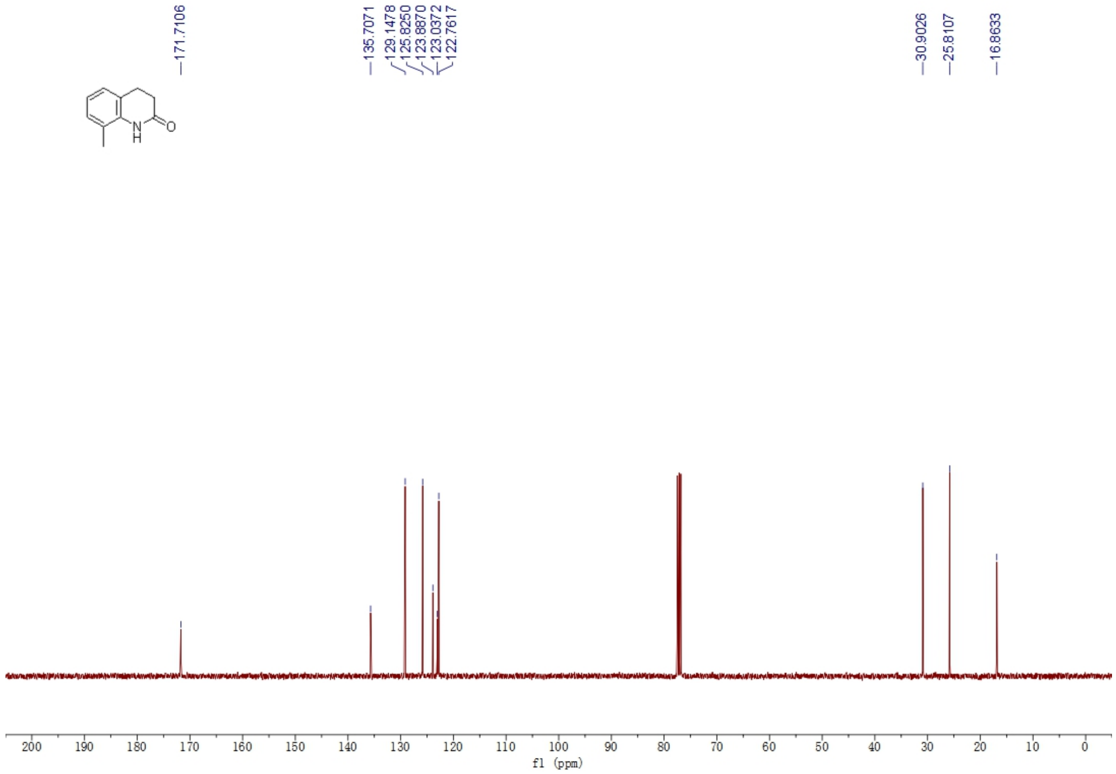

^{1}H-NMR Spectrum ($CDCl_3$) of 2c

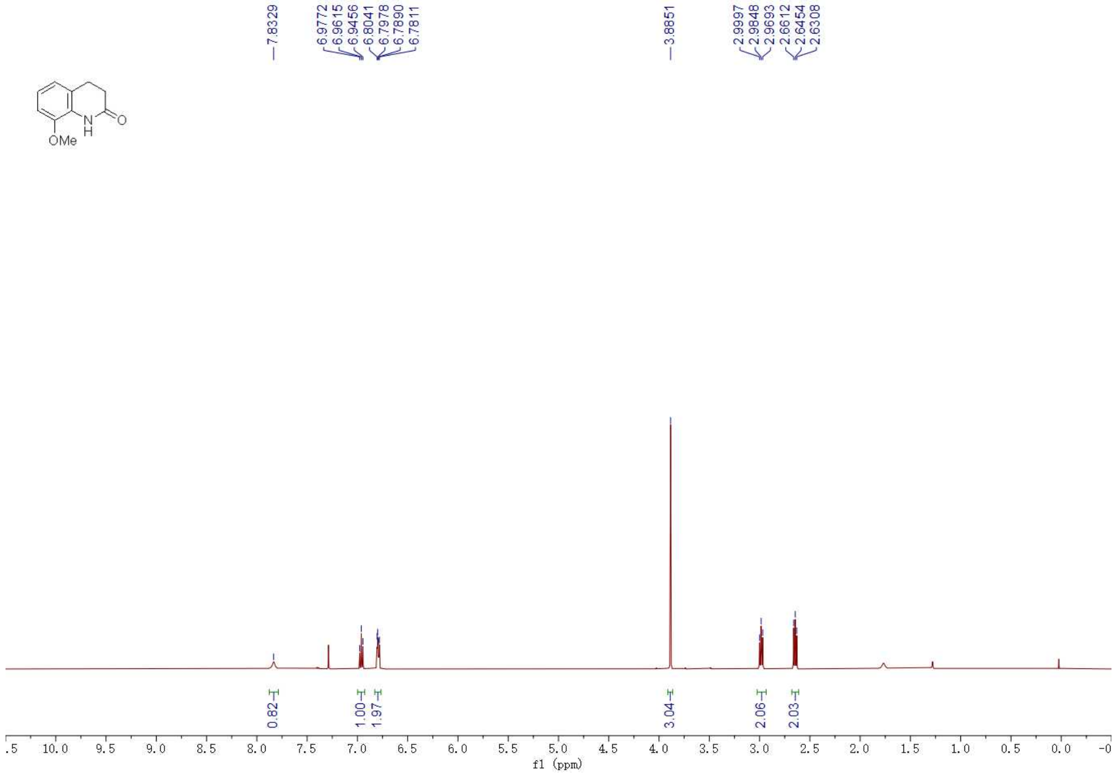

^{13}C-NMR Spectrum ($CDCl_3$) of 2c

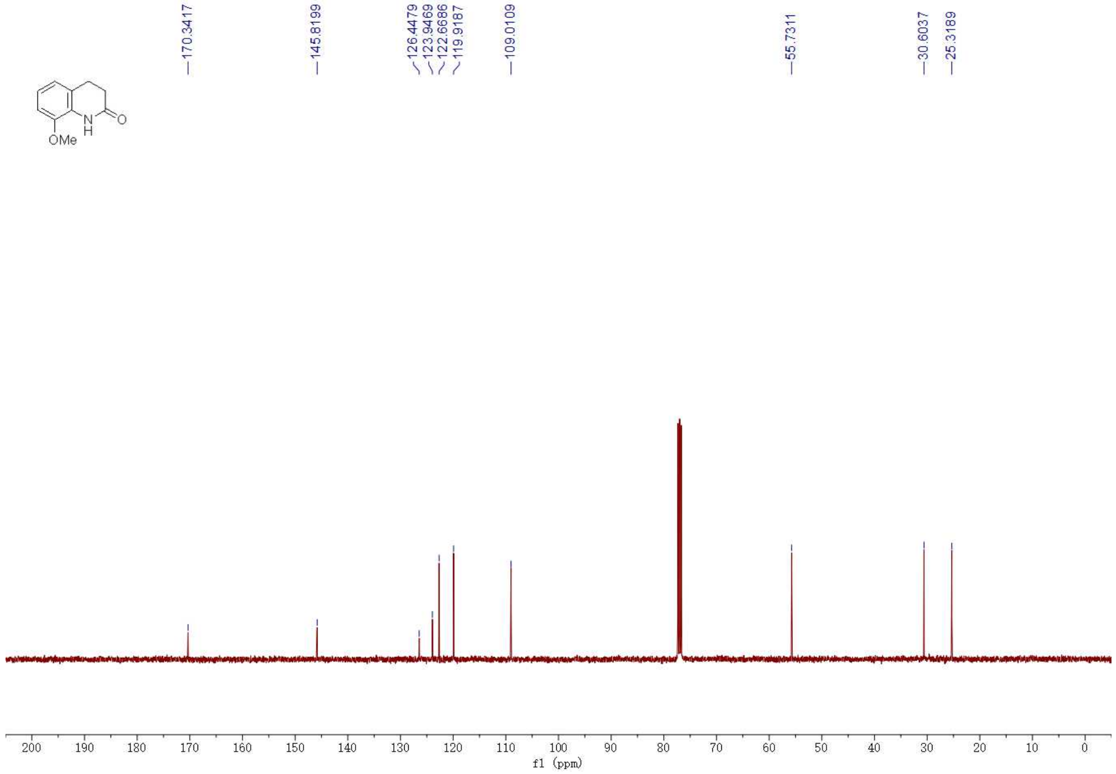

^{1}H-NMR Spectrum ($CDCl_3$) of 2d

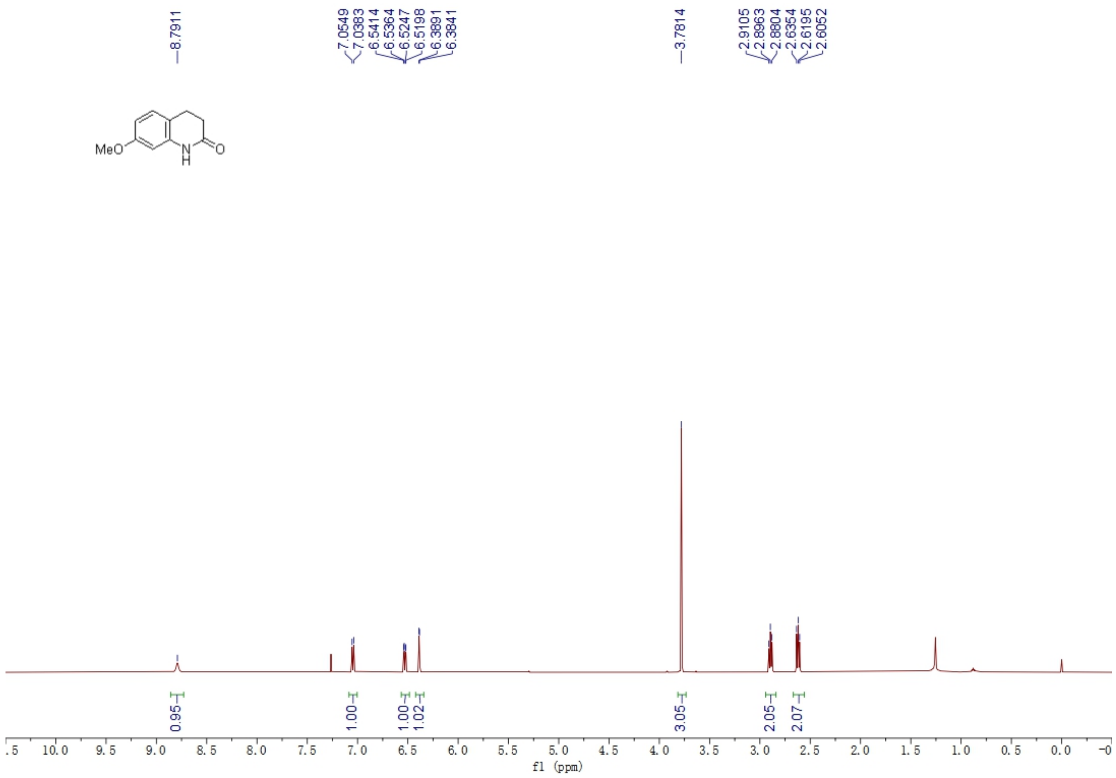

^{13}C-NMR Spectrum ($CDCl_3$) of 2d

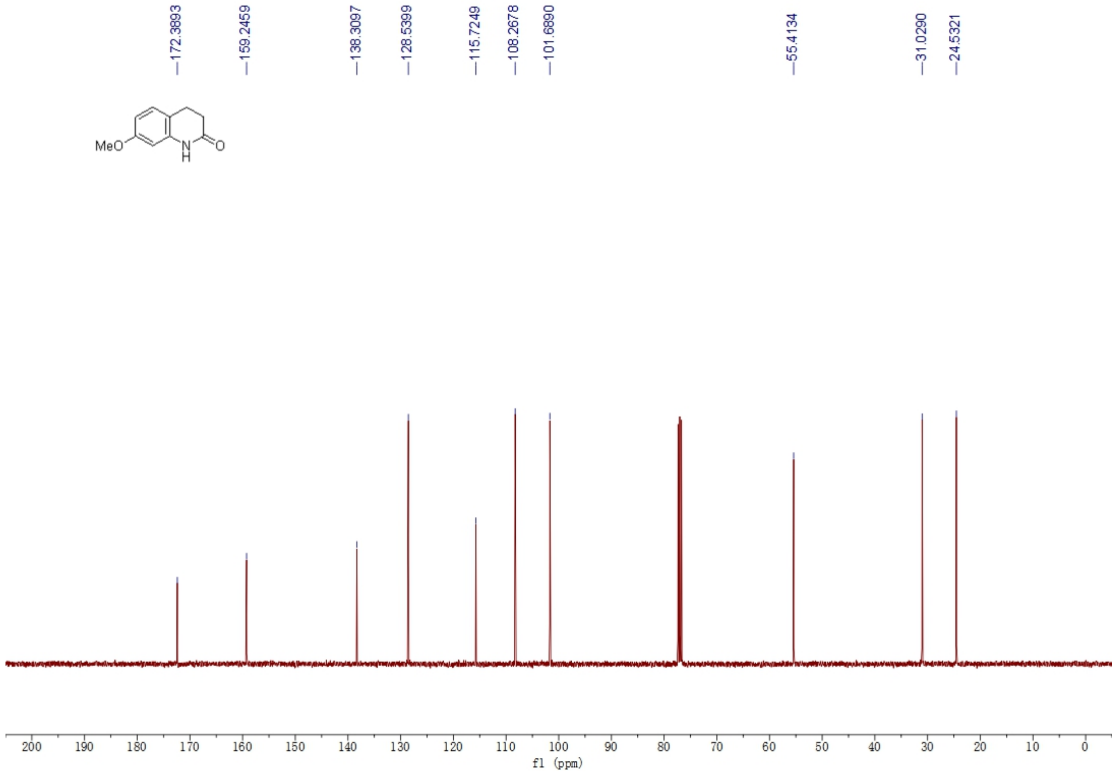

^{1}H-NMR Spectrum ($CDCl_3$) of 2e

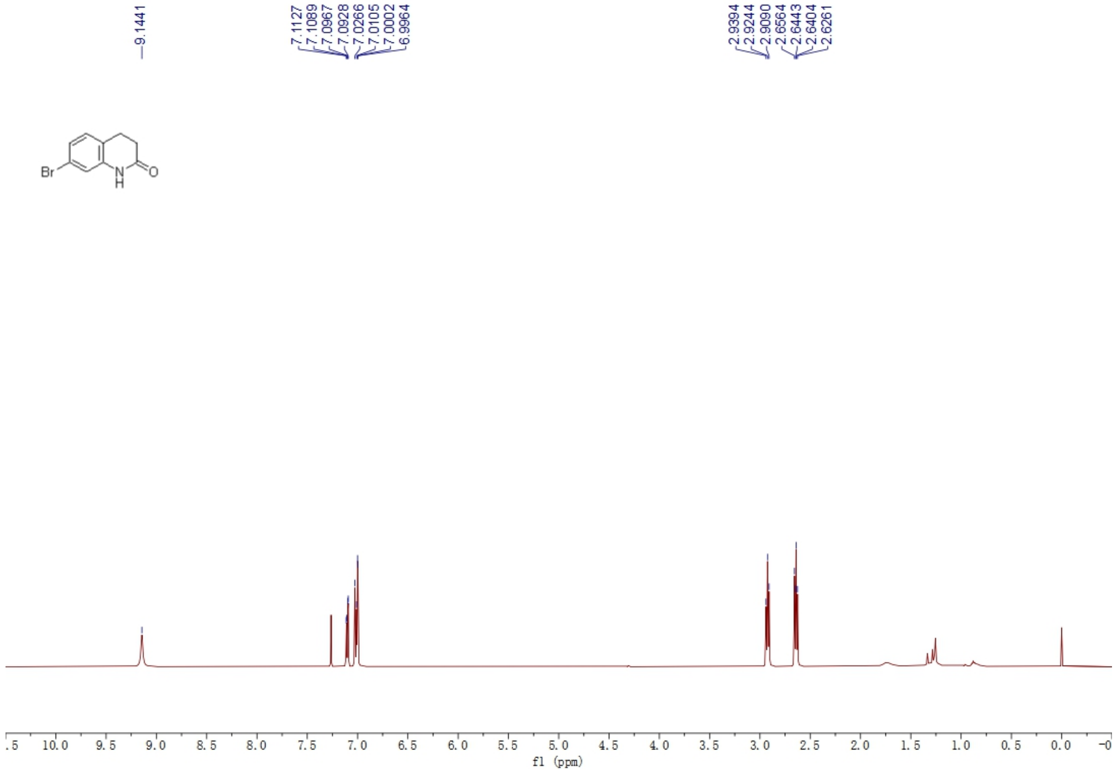

^{13}C-NMR Spectrum ($CDCl_3$) of 2e

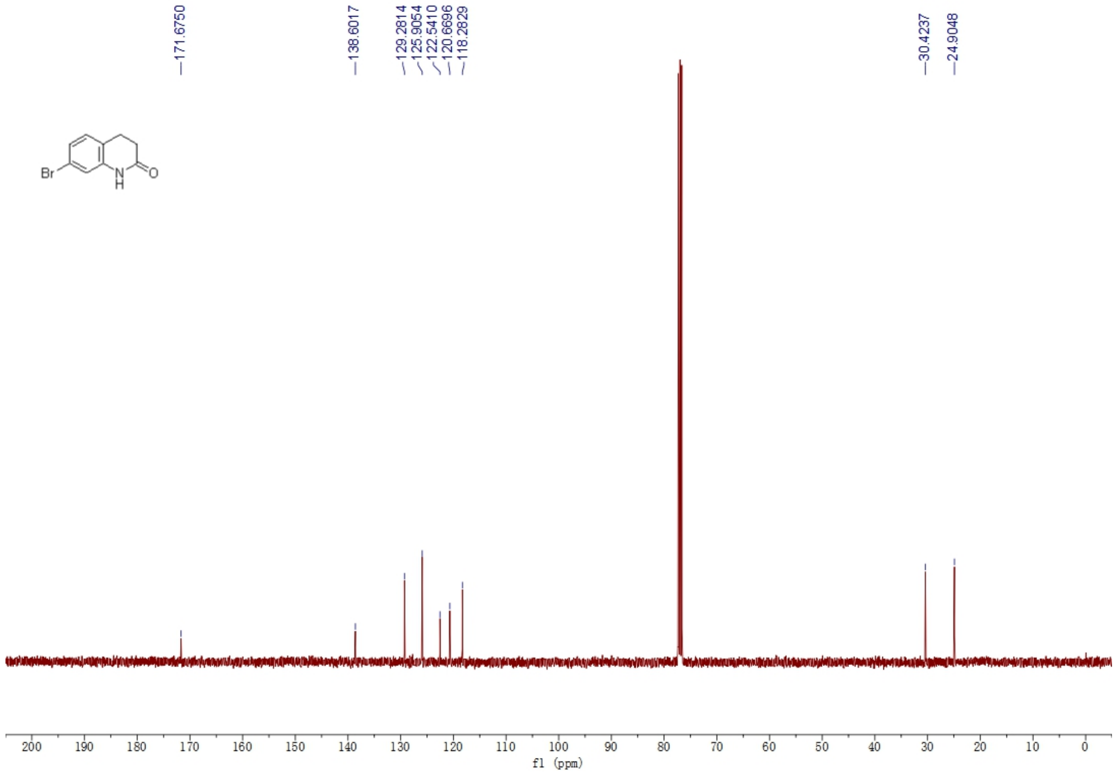

^{1}H-NMR Spectrum ($CDCl_3$) of 2f

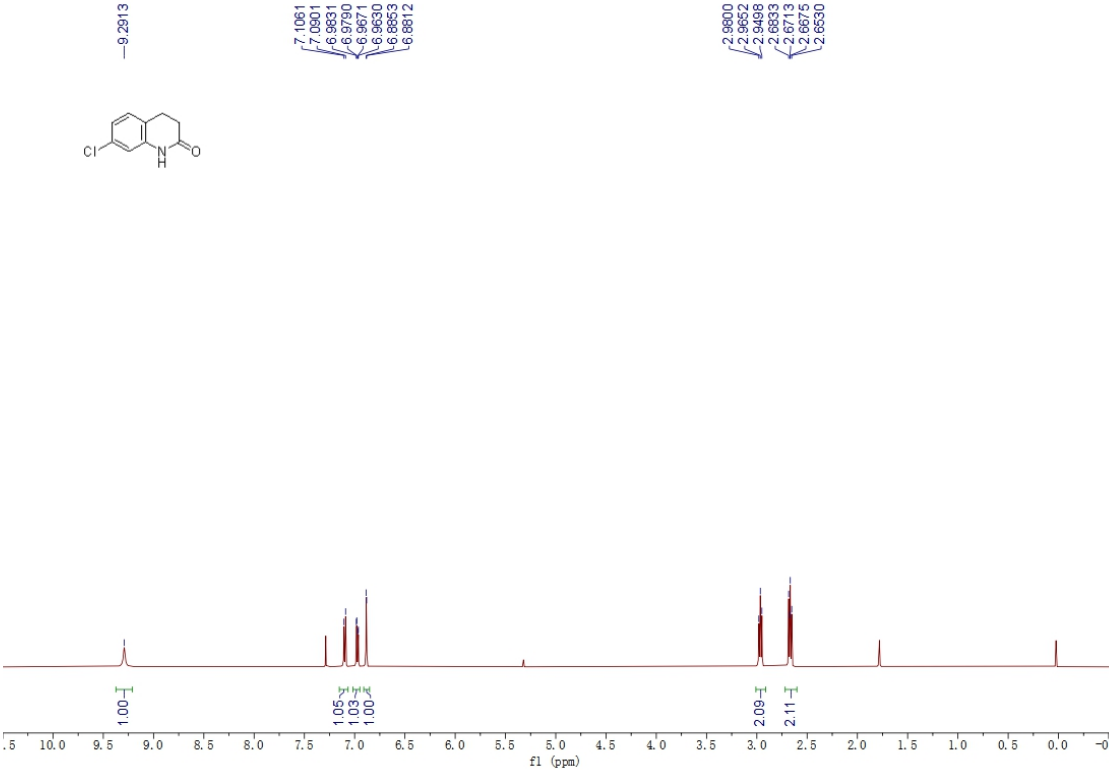

^{13}C-NMR Spectrum ($CDCl_3$) of 2f

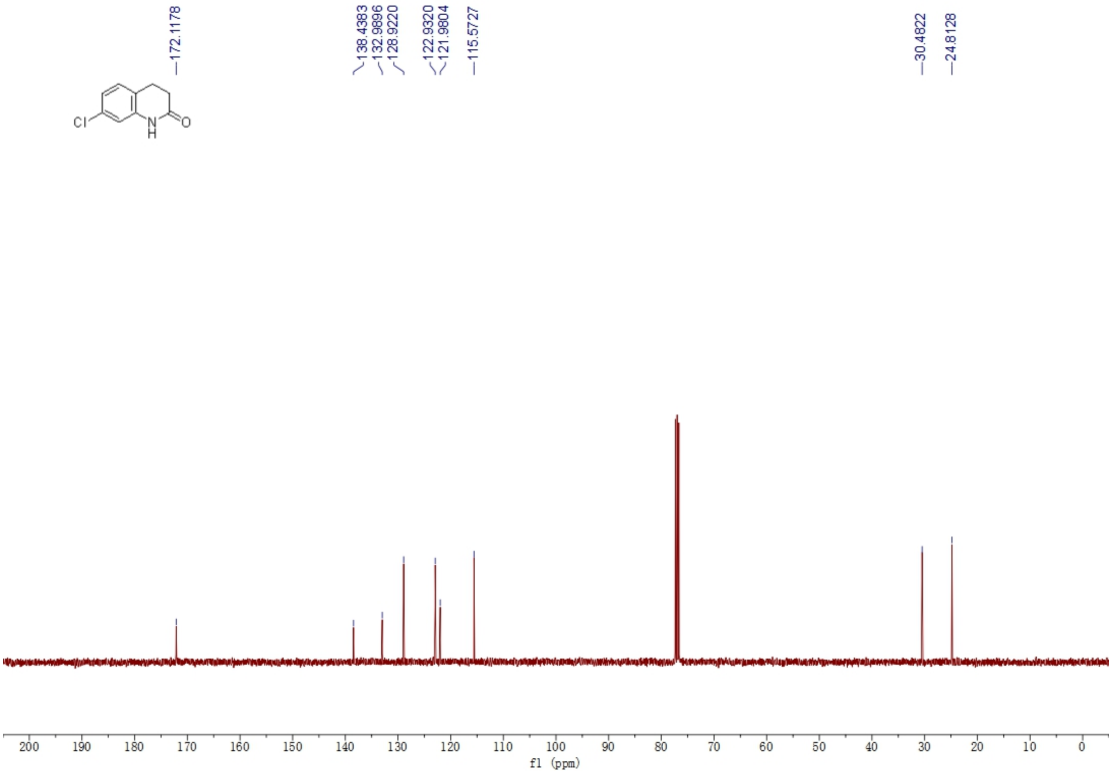

^{1}H-NMR Spectrum ($CDCl_3$) of 2g

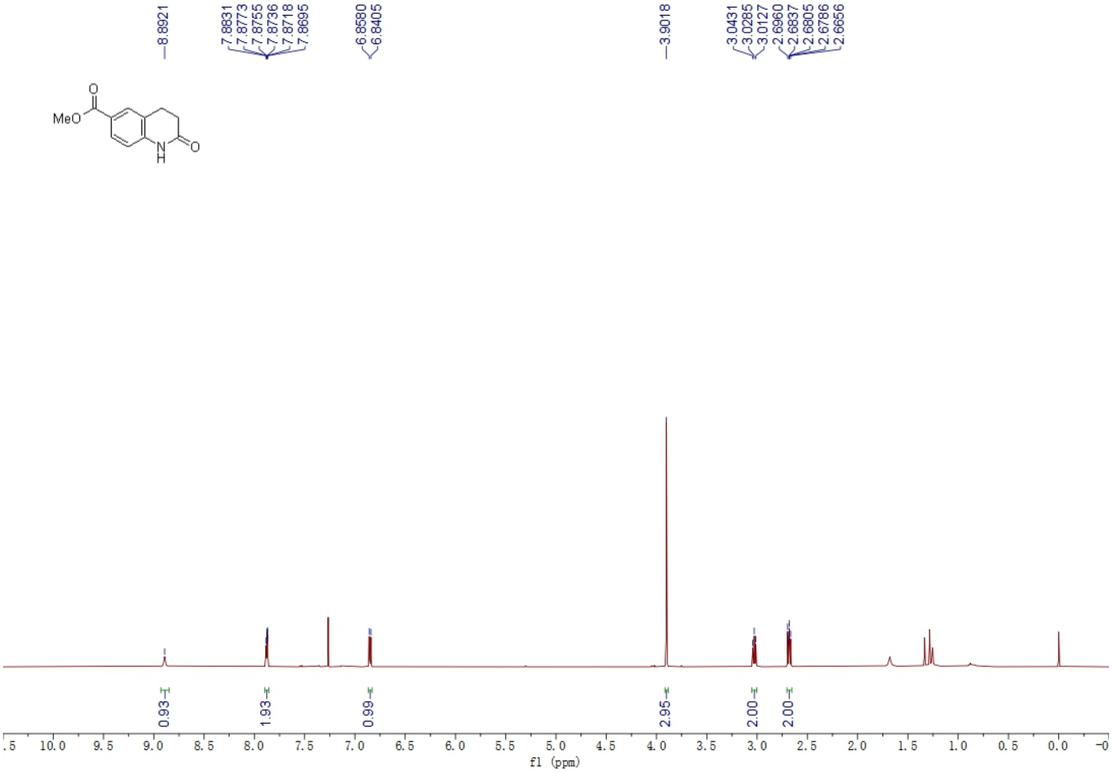

^{13}C-NMR Spectrum ($CDCl_3$) of 2g

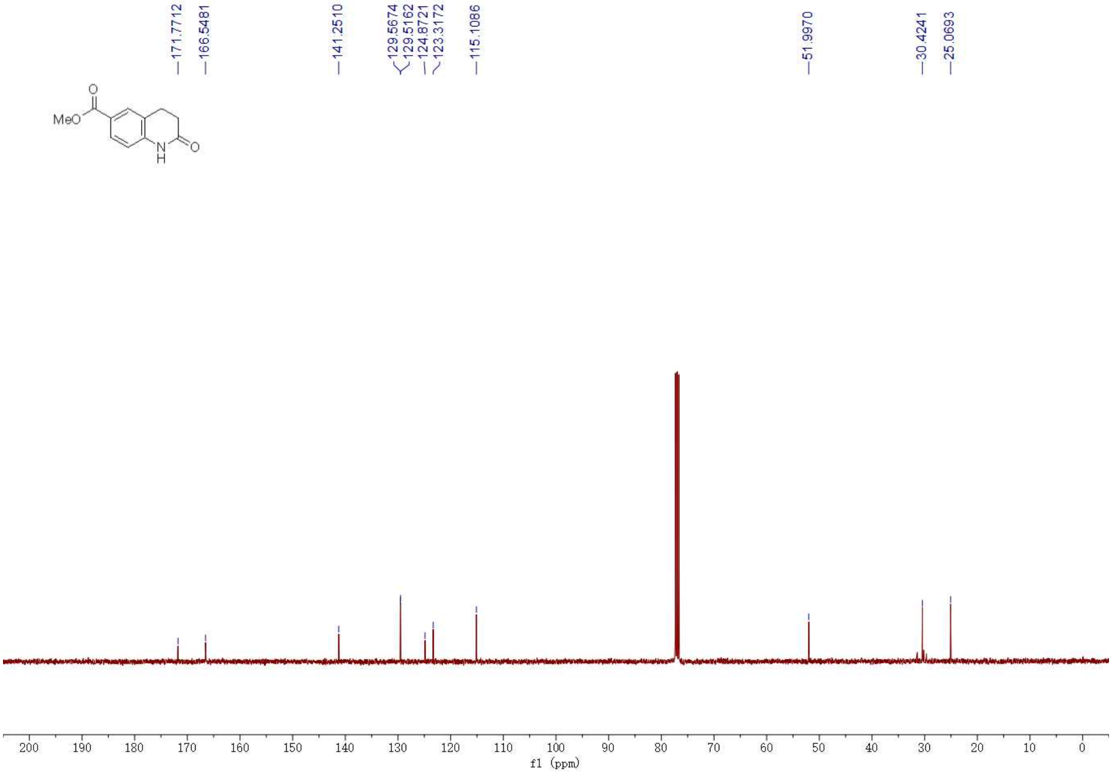

^{1}H-NMR Spectrum ($CDCl_3$) of 2h

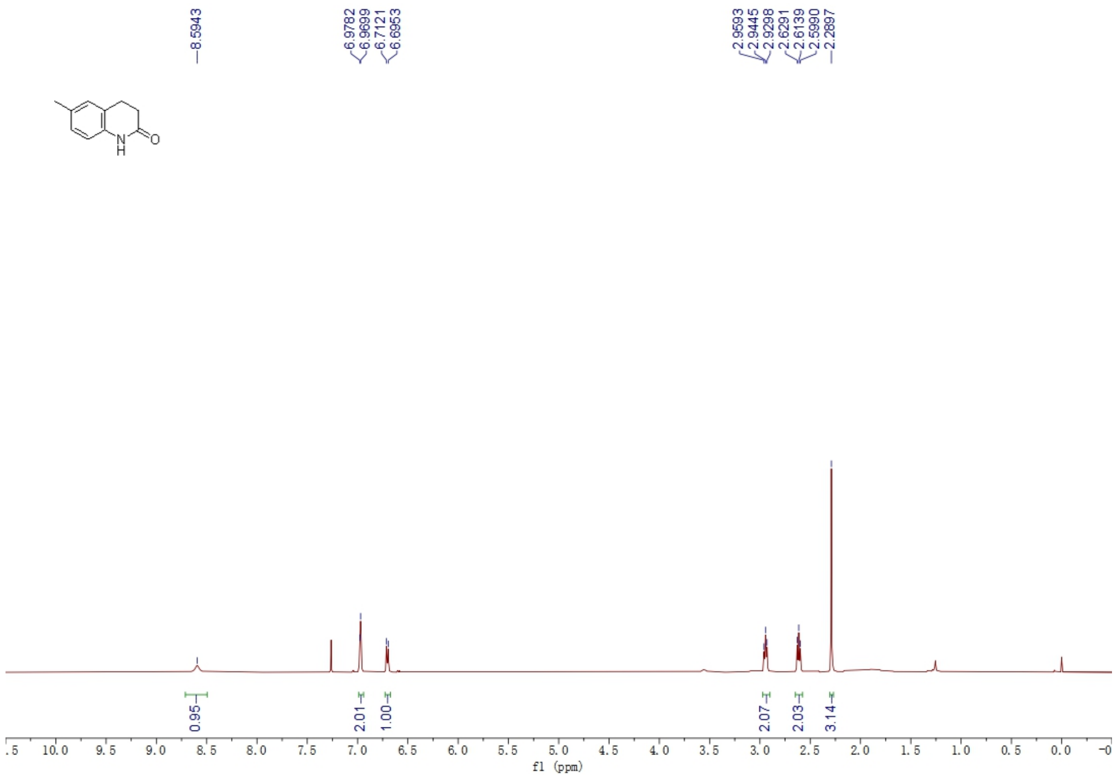

^{13}C-NMR Spectrum ($CDCl_3$) of 2h

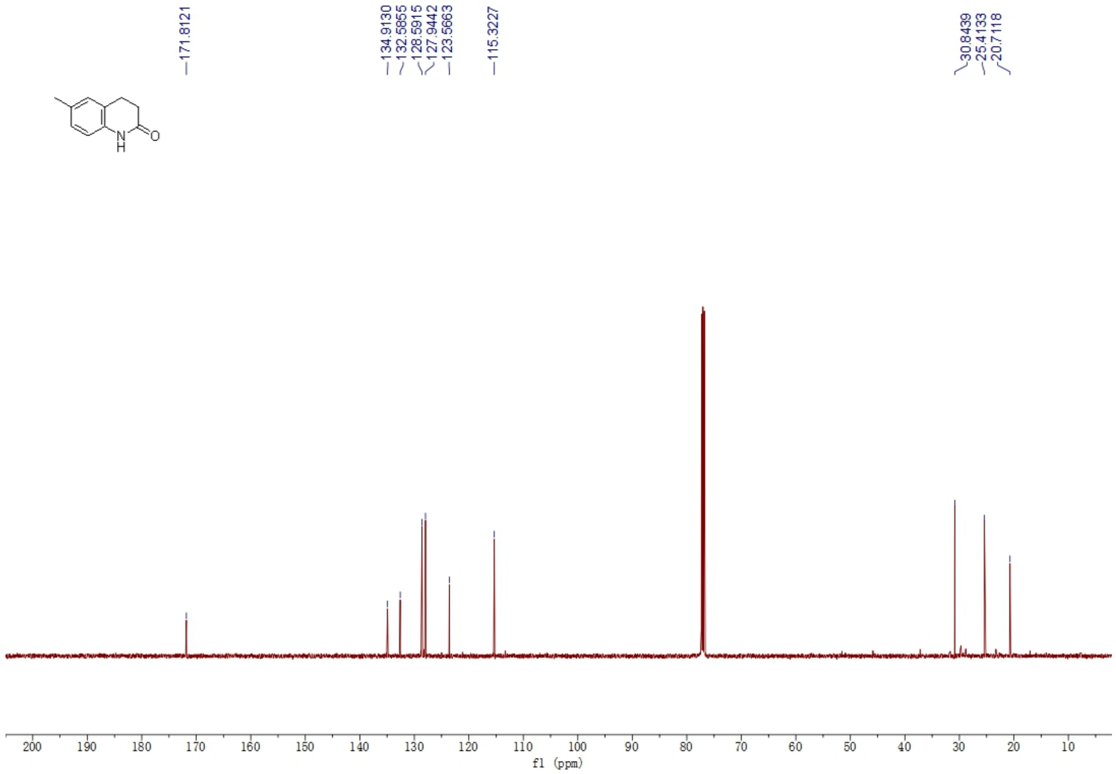

^{1}H-NMR Spectrum (DMSO) of 2i

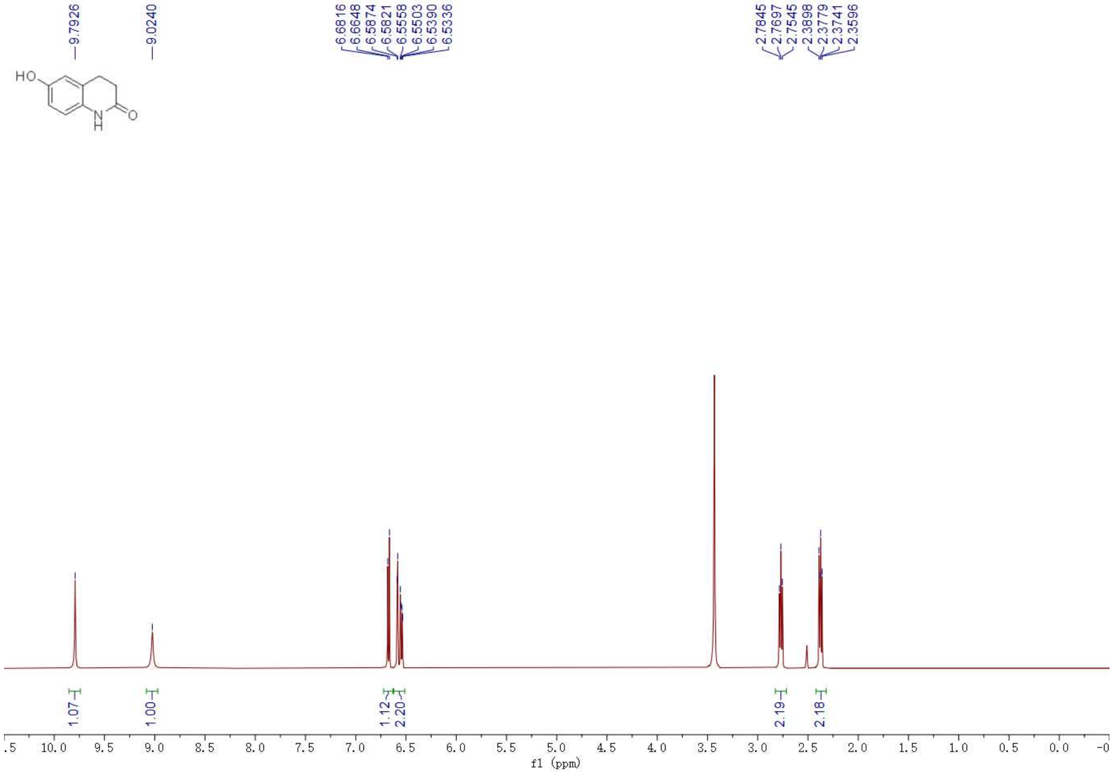

^{13}C-NMR Spectrum (DMSO) of 2i

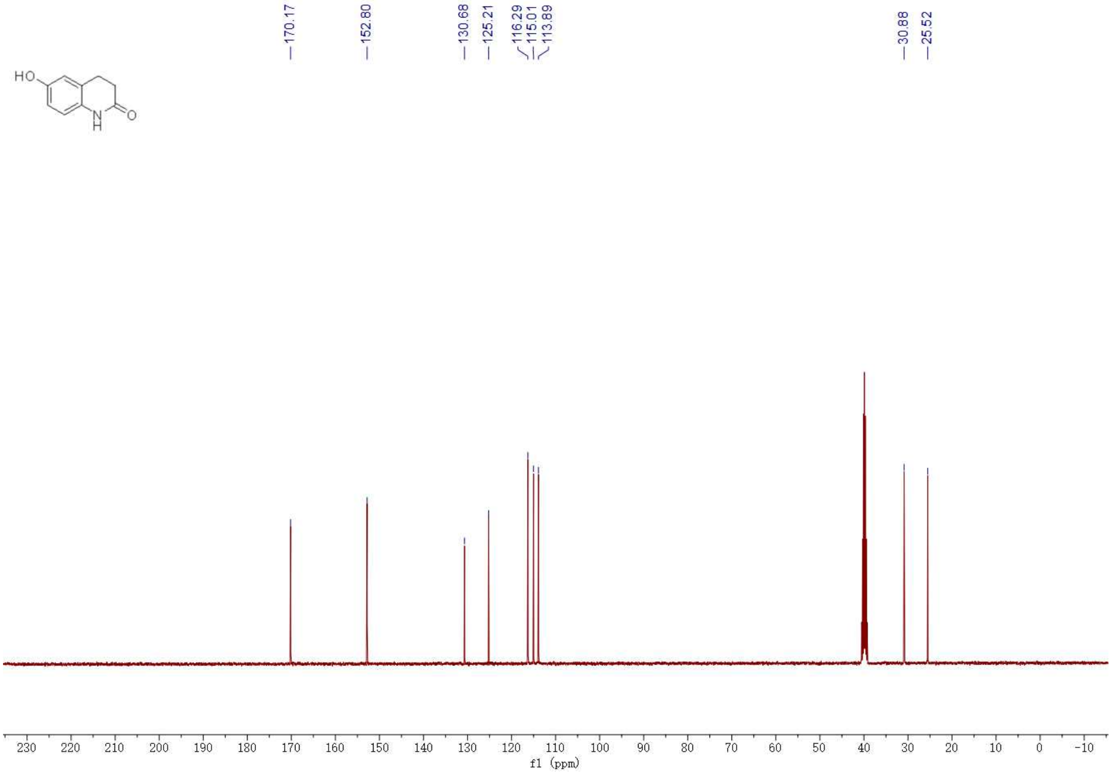

^{1}H-NMR Spectrum ($CDCl_3$) of 2j

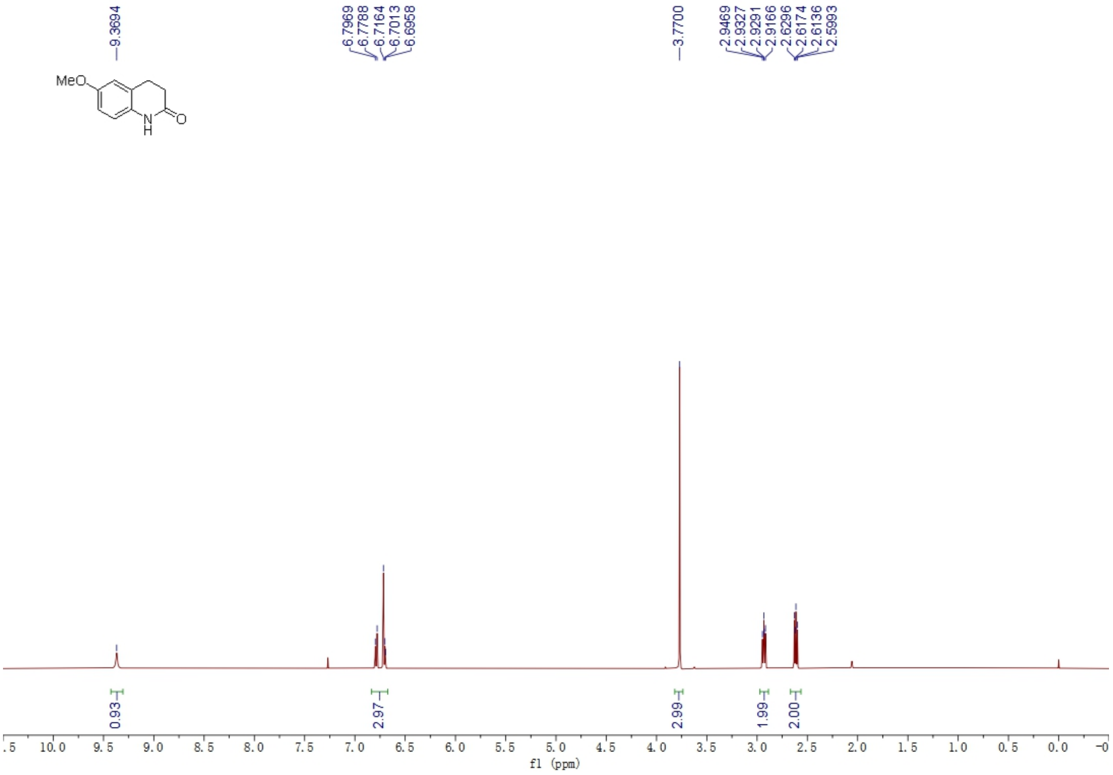

^{13}C-NMR Spectrum ($CDCl_3$) of 2j

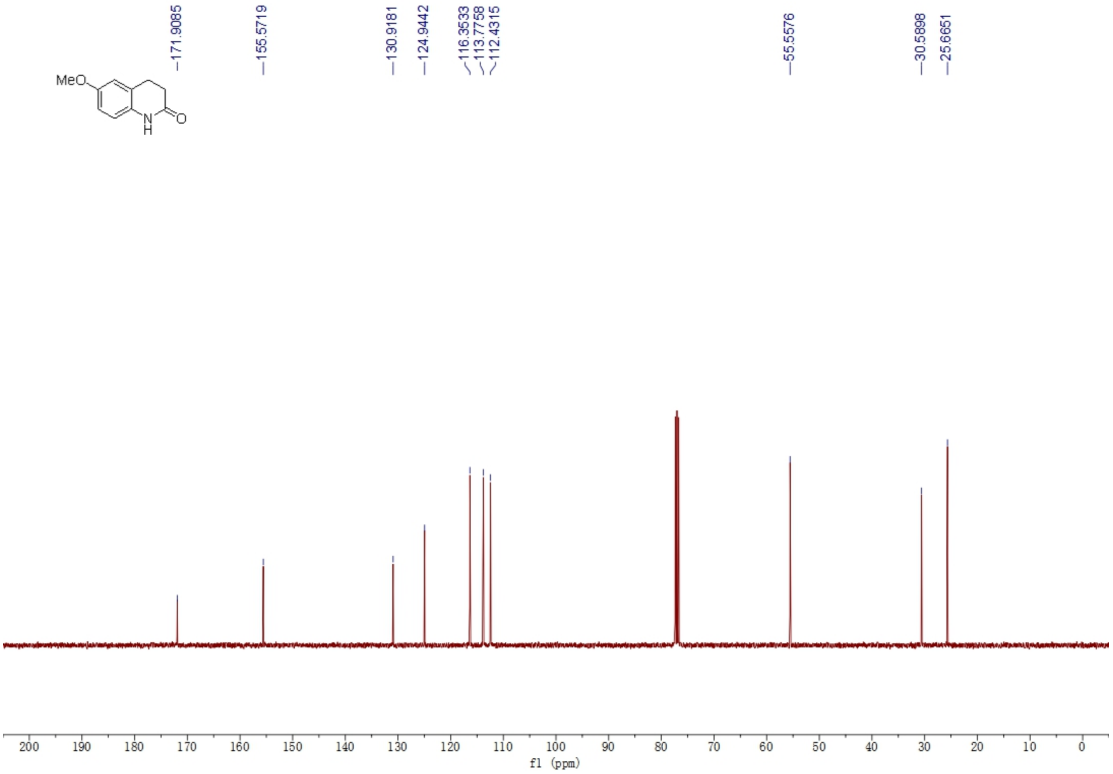

^{1}H-NMR Spectrum ($CDCl_3$) of 2k

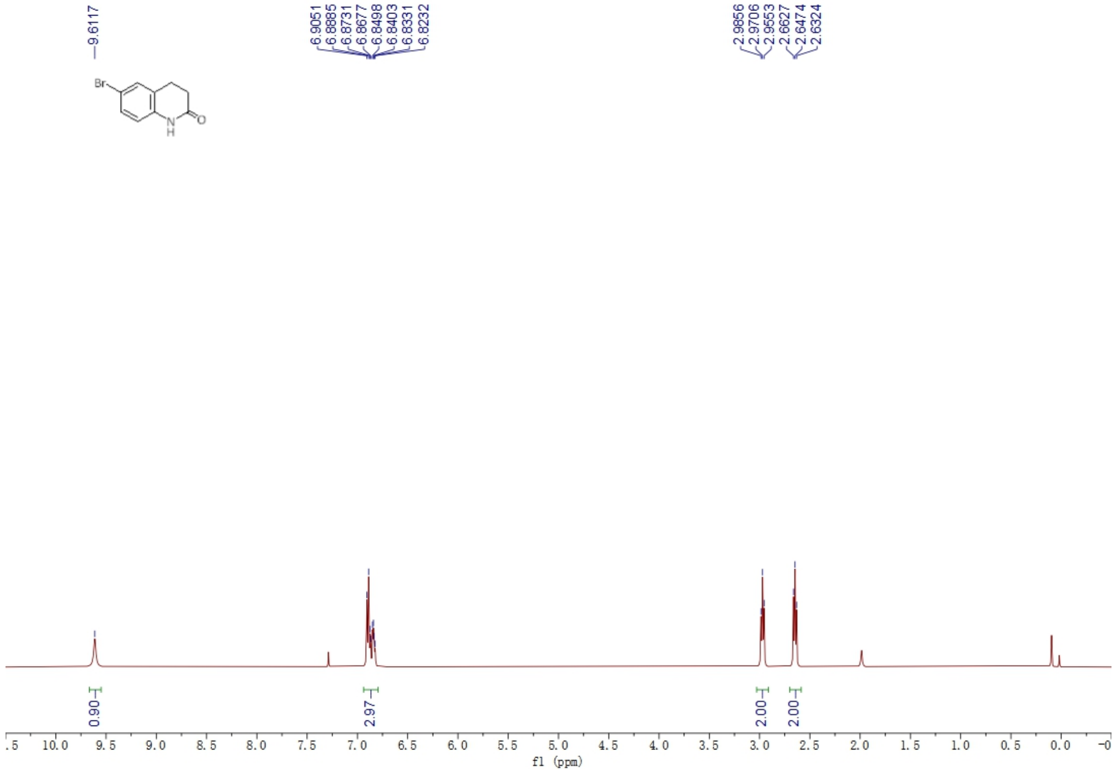

^{13}C-NMR Spectrum ($CDCl_3$) of 2k

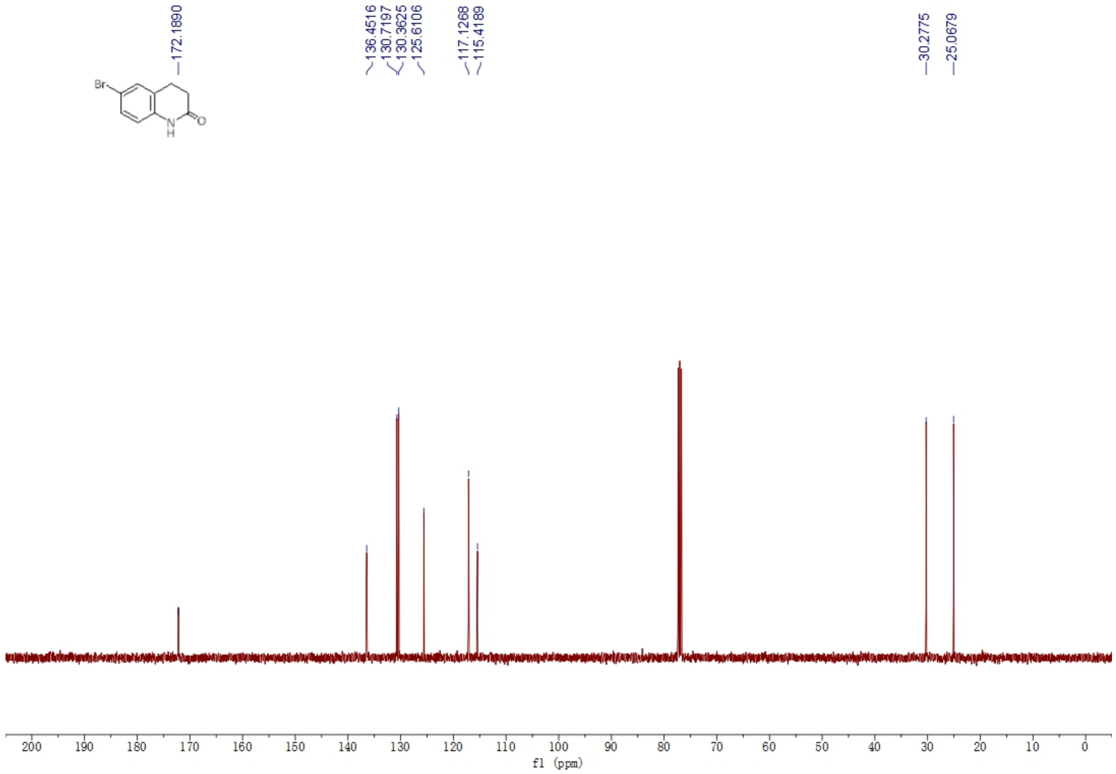

^{1}H-NMR Spectrum ($CDCl_3$) of 2l

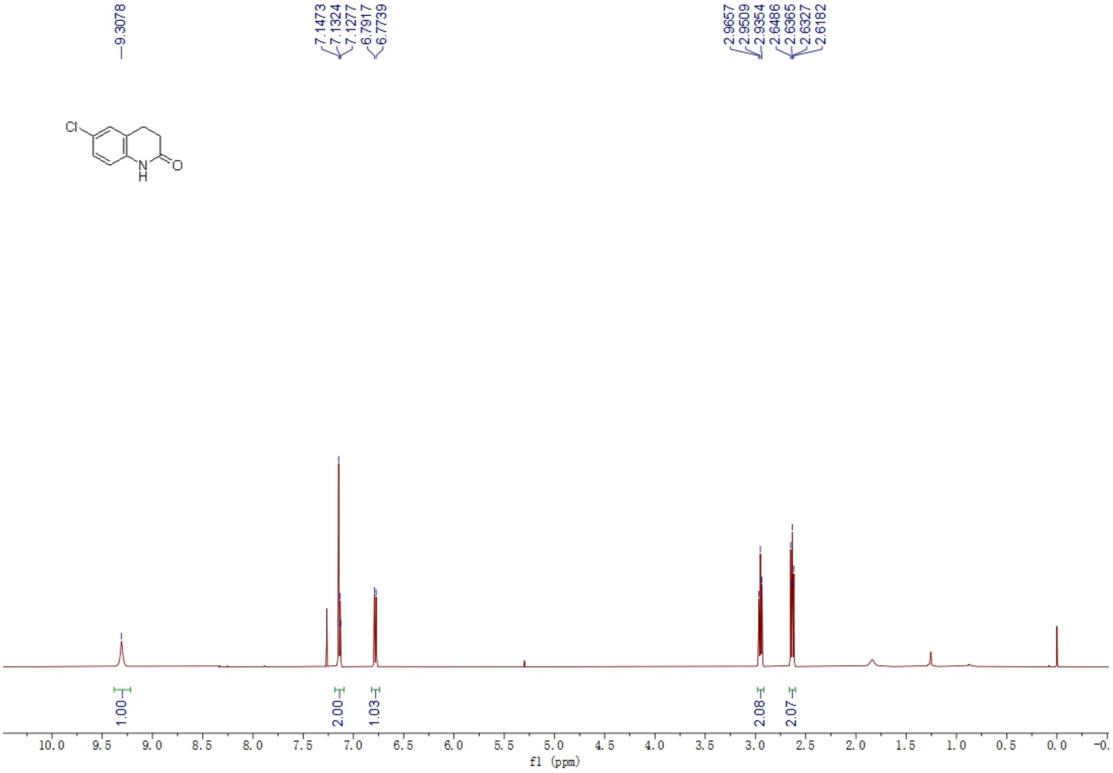

^{13}C-NMR Spectrum ($CDCl_3$) of 2l

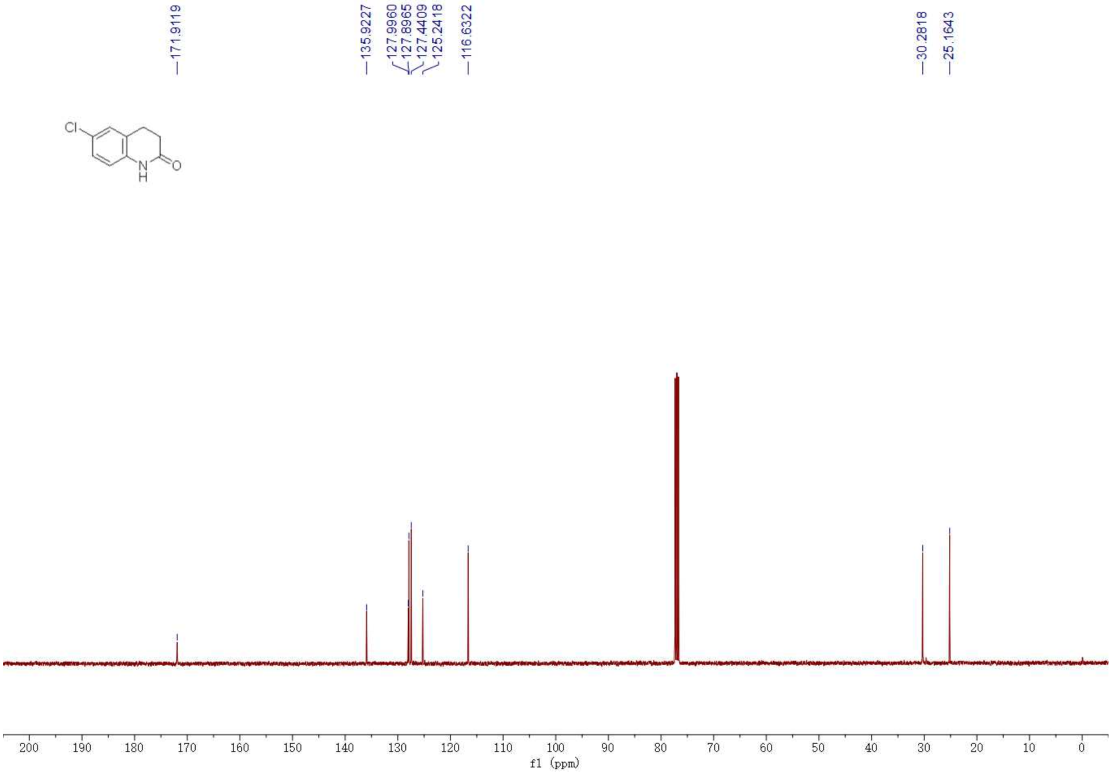

^{1}H-NMR Spectrum ($CDCl_3$) of 2m

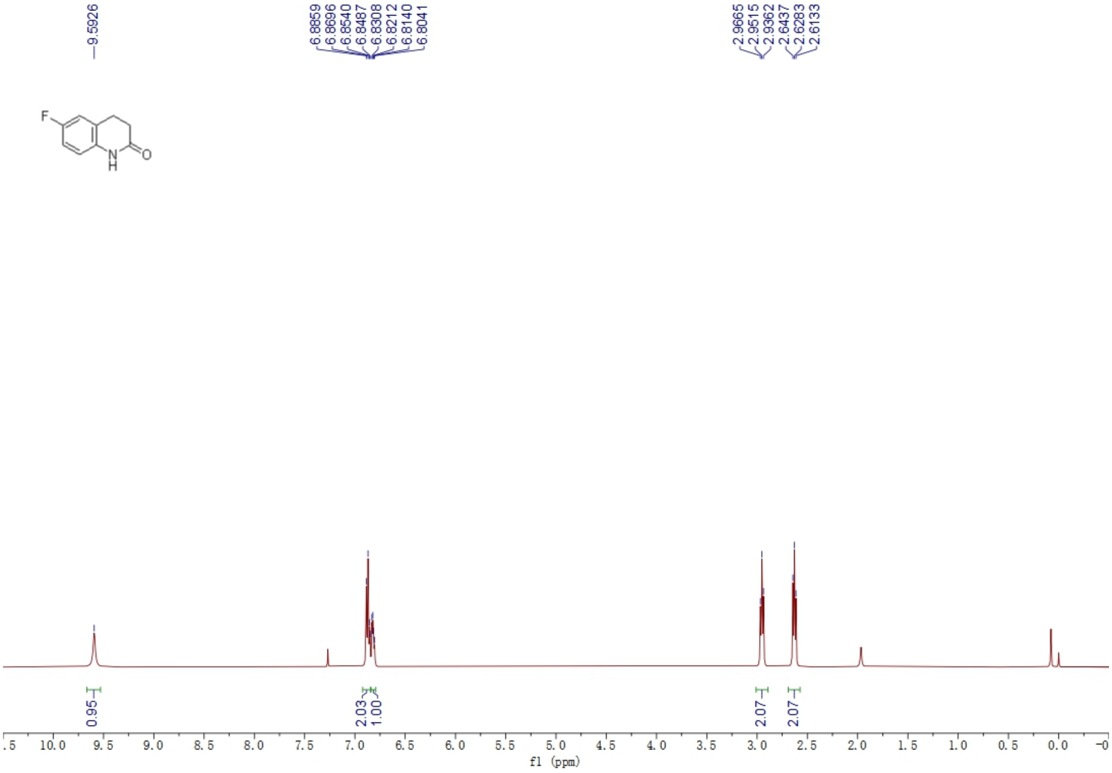

^{13}C-NMR Spectrum ($CDCl_3$) of 2m

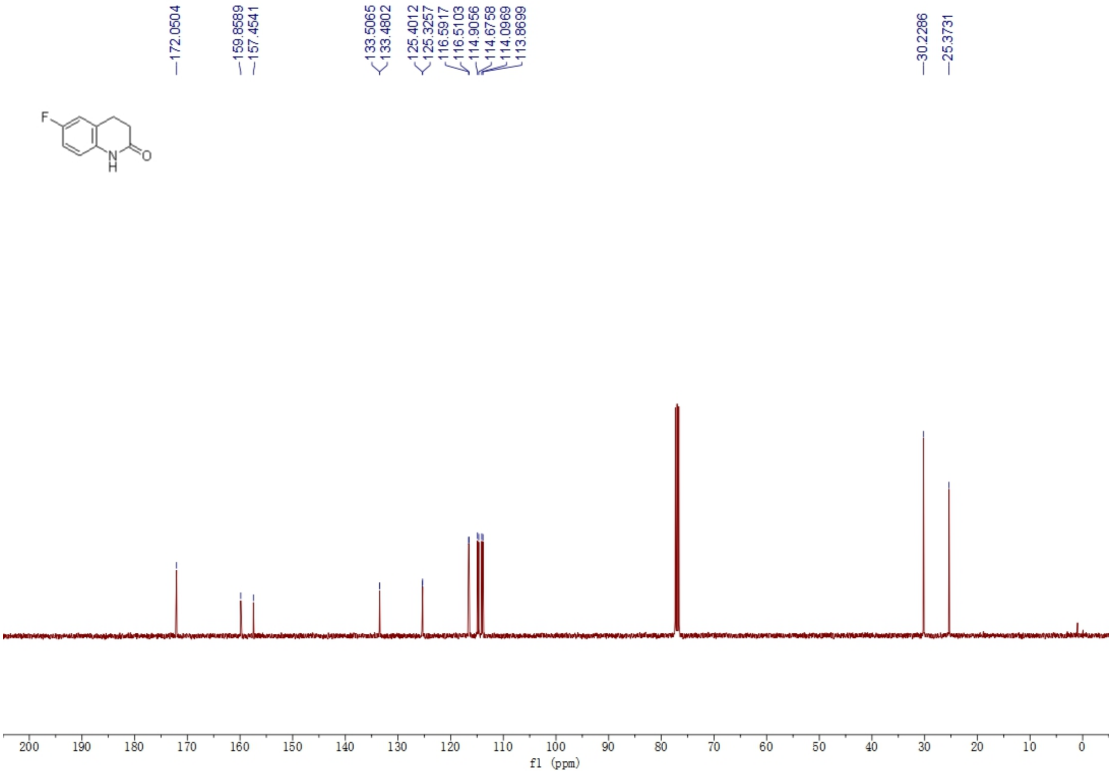

^{19}F-NMR Spectrum ($CDCl_3$) of 2m

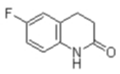

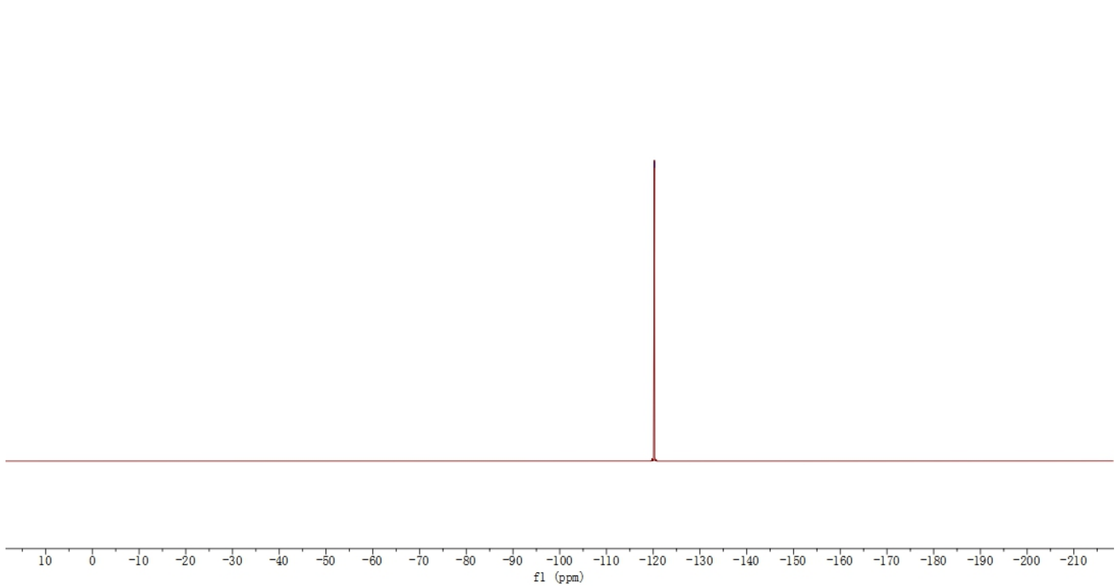

^{1}H-NMR Spectrum ($CDCl_3$) of 2n

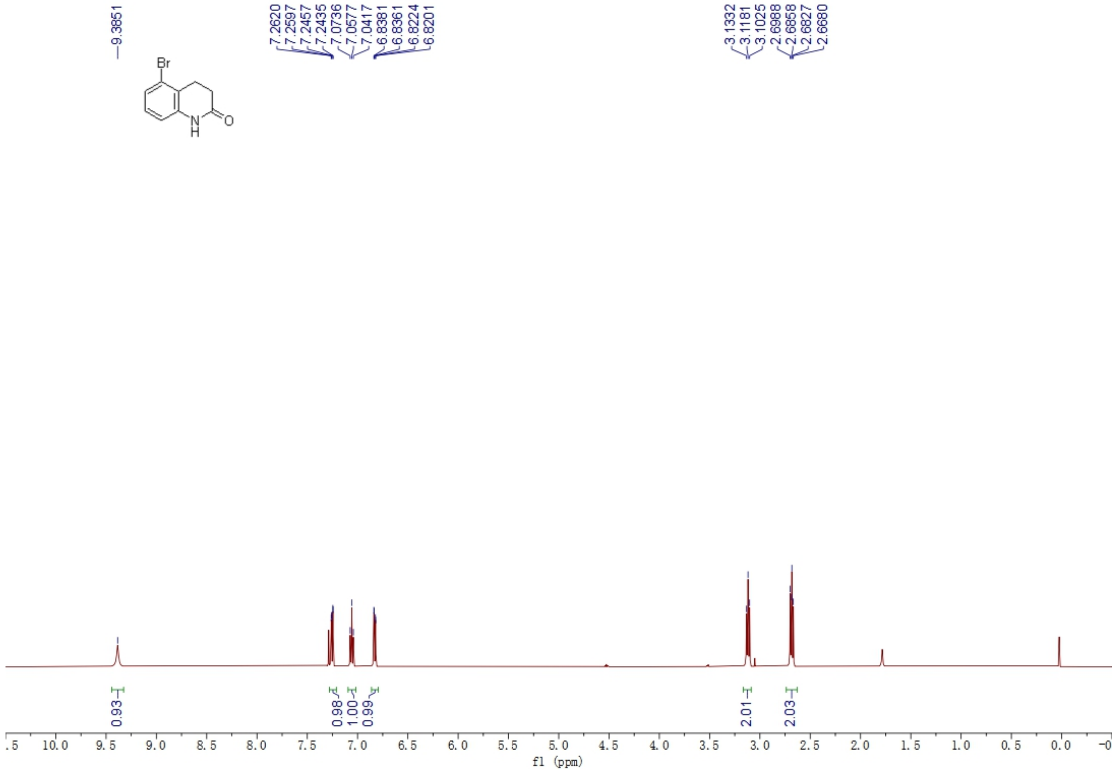

^{13}C-NMR Spectrum ($CDCl_3$) of 2n

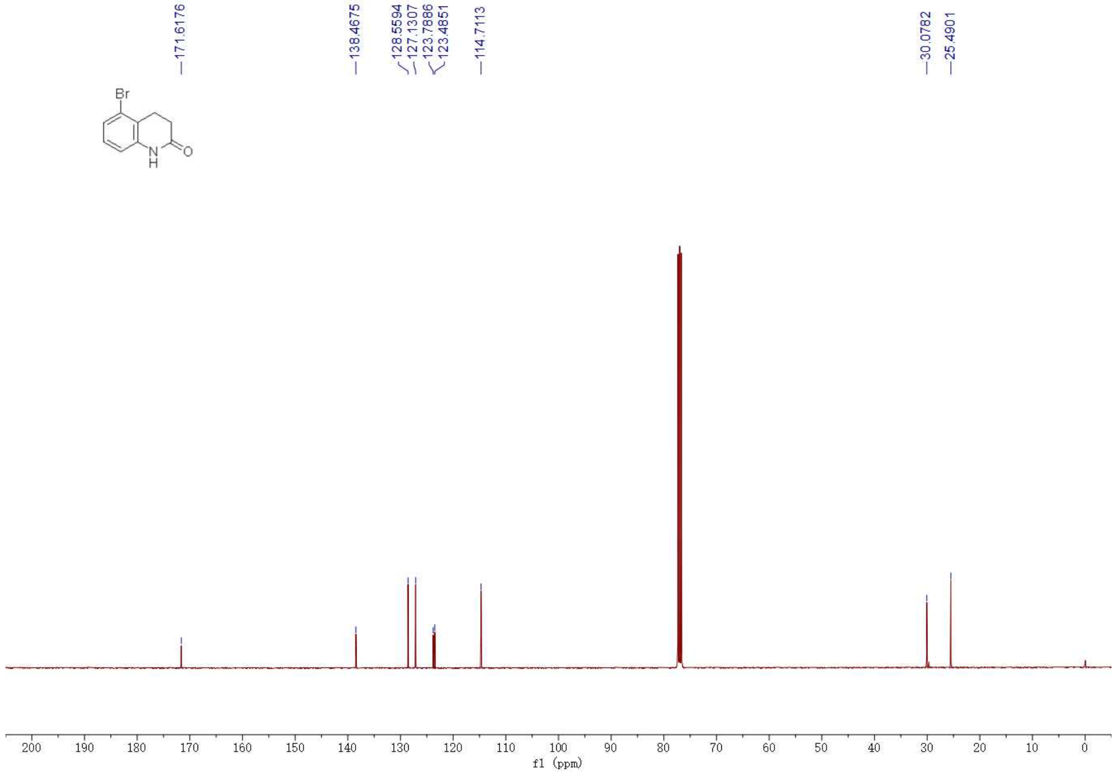

^{1}H-NMR Spectrum ($CDCl_3$) of 2o

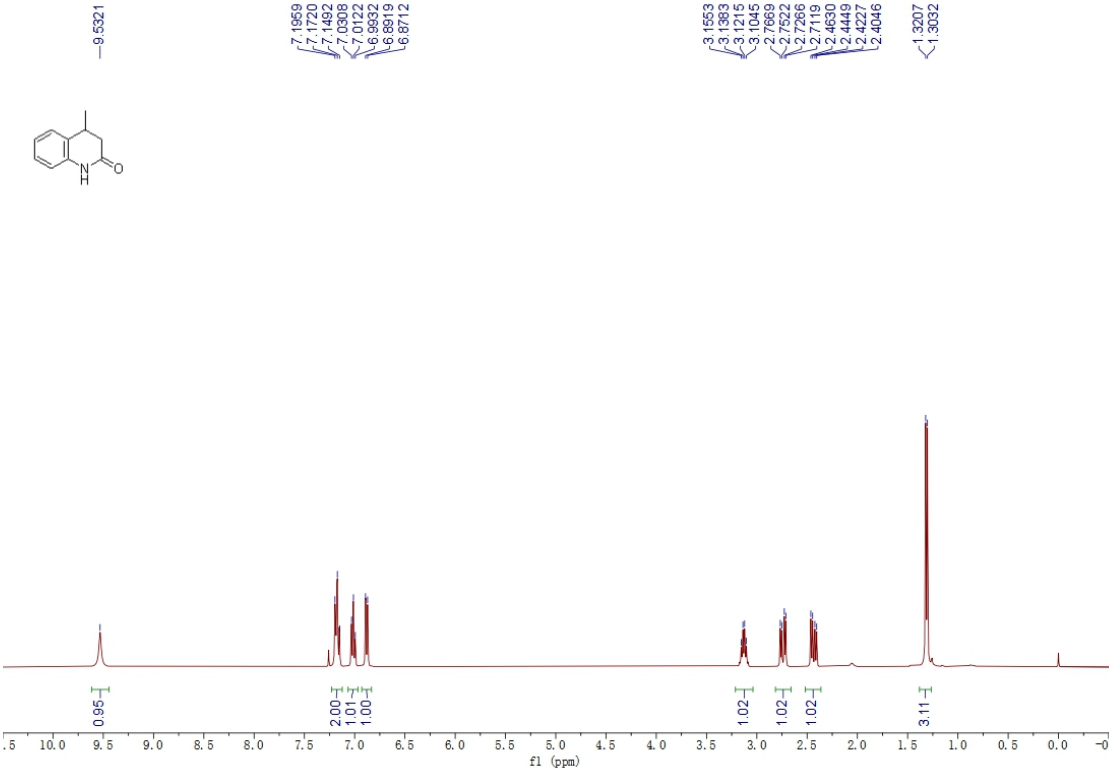

^{13}C-NMR Spectrum ($CDCl_3$) of 2o

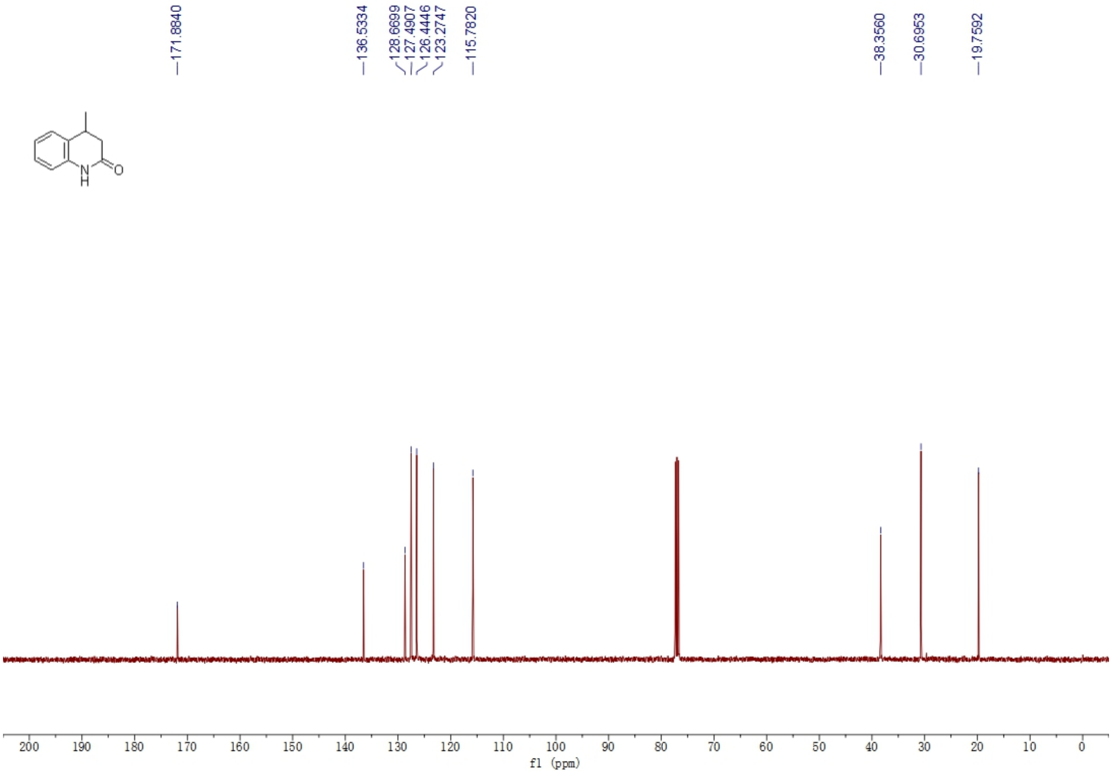

^{1}H-NMR Spectrum ($CDCl_3$) of 2p

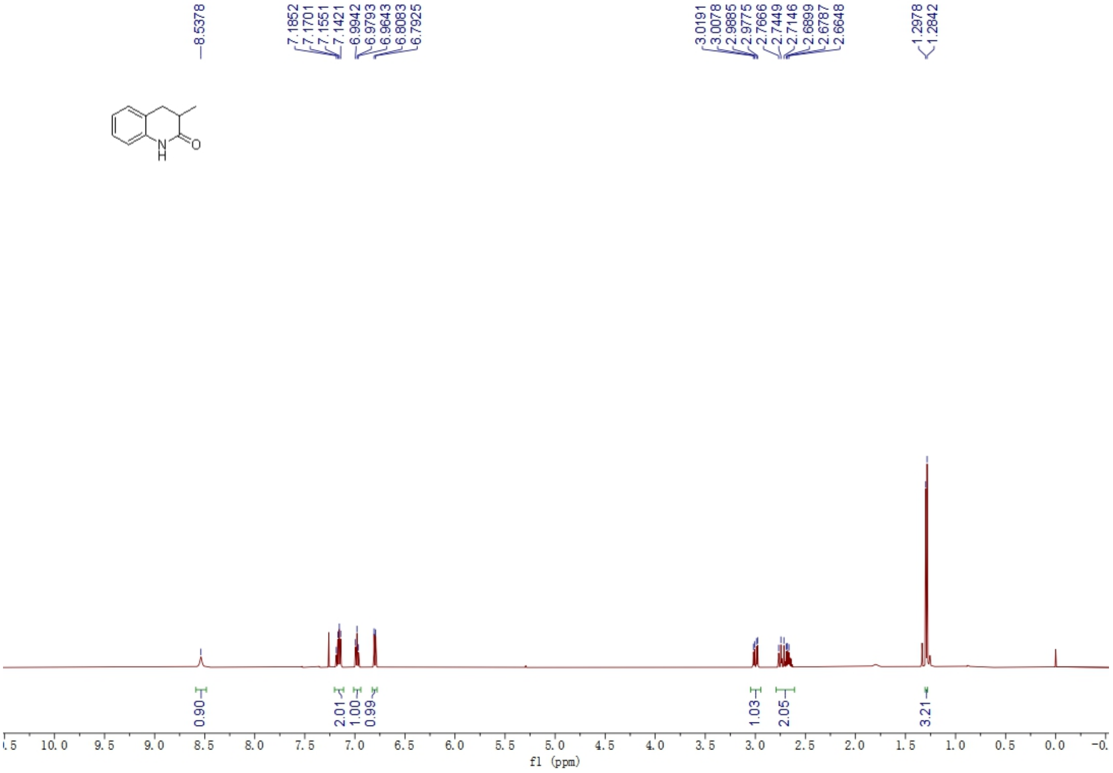

^{13}C-NMR Spectrum ($CDCl_3$) of 2p

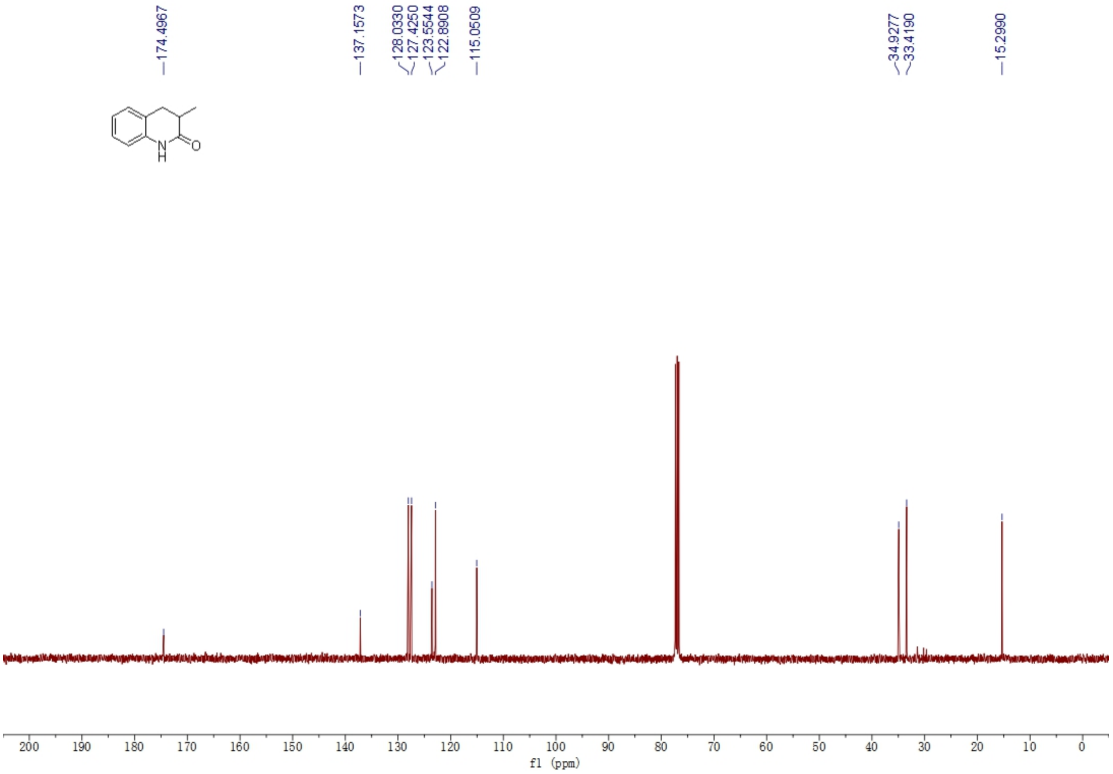

^{1}H-NMR Spectrum ($CDCl_3$) of 2q

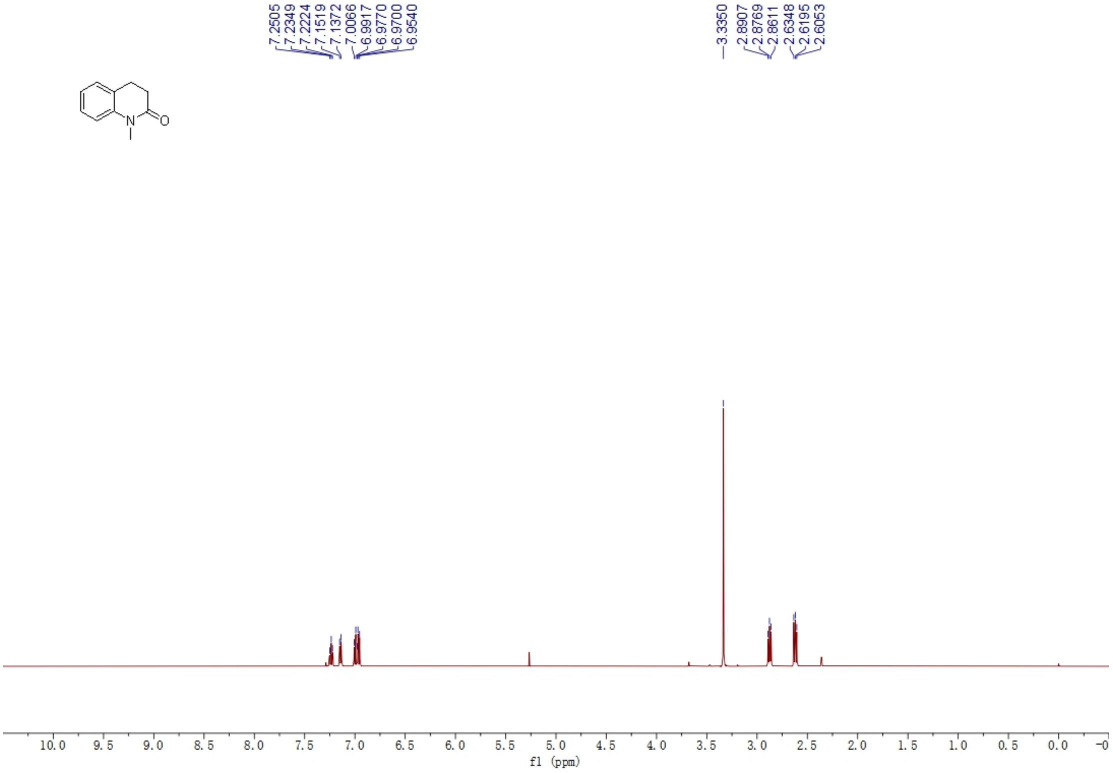

^{13}C-NMR Spectrum ($CDCl_3$) of 2q

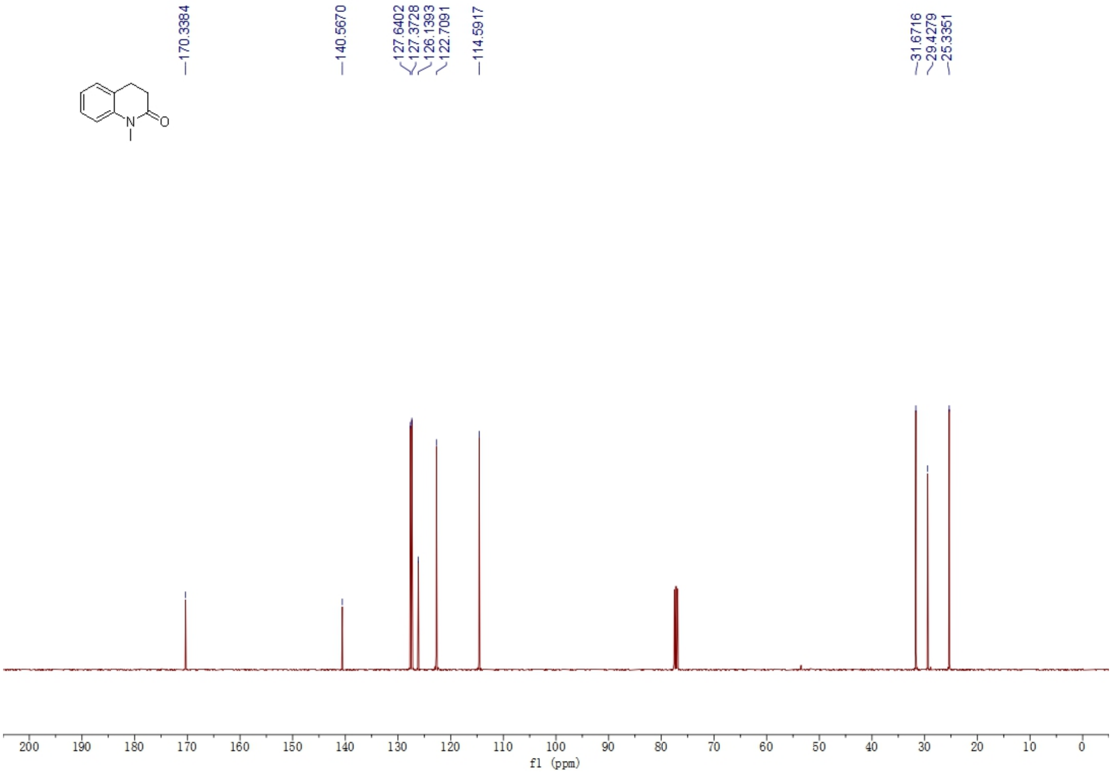

8. DFT calculated cartesian coordinates

$[Ru(bpy)_3]^{2+}$

Nimag=0

Ru	0.00002000	0.00089400	-0.00010200
N	-0.83669700	-1.62466100	-1.04928100
C	-0.50701000	-2.84961400	-0.58195700
C	-1.01226900	-4.00754800	-1.17591100
C	-1.87018900	-3.89707900	-2.26639300
C	-2.20380600	-2.63008700	-2.73748500
C	-1.66384500	-1.52000800	-2.09633400
H	-0.74368500	-4.99069800	-0.79377200
H	-2.27308800	-4.79261100	-2.74082000
H	-2.87167200	-2.49551800	-3.58771700
H	-1.89503500	-0.50636800	-2.43049700
N	1.82445000	0.08965700	-1.05220600
N	-0.99201800	1.53727600	-1.04902800
N	0.78424600	-1.65066100	1.04904800
N	-1.82059300	0.14787300	1.05203600
N	1.04076700	1.50450900	1.04909500
C	0.41528500	-2.86441000	0.58184800
C	2.72215300	0.98579900	-0.58473200
C	2.14507300	-0.67805700	-2.10063000
C	-2.21768400	1.86289700	-0.58065800
C	-0.48966300	2.20140200	-2.09684900
C	1.61443400	-1.57257900	2.09601700
C	-2.68915400	1.07237100	0.58472800
C	-2.16560400	-0.60929600	2.10039700
C	2.27623300	1.79080300	0.58080200
C	0.55995000	2.18405500	2.09711400
C	0.88303900	-4.03794800	1.17589900
C	3.97699900	1.12645900	-1.18013600
C	3.37570000	-0.59138000	-2.74324100
H	1.38155200	-1.38381800	-2.43459900
C	-2.97004400	2.87764000	-1.17485900
C	-1.18311700	3.22261800	-2.73809300
H	0.50403800	1.89621100	-2.43149700
C	2.11841700	-2.69939900	2.73724900
H	1.87816900	-0.56686800	2.43004600
C	-3.93882700	1.25307700	1.18019800
C	-3.39279700	-0.48331400	2.74306900
H	-1.42506100	-1.33917200	2.43426800
C	3.06071000	2.78076600	1.17528200
C	1.28575600	3.18235000	2.73865700
H	-0.44297200	1.91065300	2.43173100
C	1.74412600	-3.95503800	2.26632600
H	0.58294900	-5.01199300	0.79389100
C	4.30786500	0.32926300	-2.27212500
H	4.69556400	1.84919000	-0.79791100
H	3.59122700	-1.23623300	-3.59456900
C	-2.44743400	3.56535800	-2.26634800
H	-3.95586300	3.13543700	-0.79216300
H	-0.73408800	3.73396600	-3.58893500
H	2.79033900	-2.58631600	3.58741100
C	-4.29501500	0.46676500	2.27209800
H	-4.63390100	1.99849200	0.79811400
H	-3.62884000	-1.12102600	3.59433400
C	2.56039600	3.48456100	2.26698100
H	4.05429000	3.00692600	0.79264900
H	0.85334800	3.70754900	3.58967200
H	2.11801000	-4.86302800	2.74082500
H	5.28438800	0.42755500	-2.74765800
H	-3.02326400	4.36068800	-2.74095400
H	3.16140800	4.26089700	2.74181400
H	-5.26787300	0.59622700	2.74767900

$[Ru(bpy)_3]^{2+*}$

Nimag=0

Ru	-0.00010500	0.01781300	0.00009700
N	-2.36265800	-0.55650800	-1.47342500
C	-3.13663800	-1.01570000	-0.48376400
C	-4.51438500	-0.78151900	-0.43613300
C	-5.10331000	-0.05188500	-1.46776200
C	-4.30001000	0.41530000	-2.50238300
C	-2.93227900	0.13748000	-2.45629600
H	-5.11658400	-1.14675900	0.39617500
H	-6.17568400	0.14757000	-1.45909200
H	-4.71763400	0.98405000	-3.33300500
H	-2.27075800	0.48796700	-3.25334400
N	1.20034100	-1.49089600	-0.95706300
N	1.01718900	1.70058400	-0.84229300
N	-1.20782100	-1.48228400	0.96084500
N	-1.01015700	1.70614200	0.84120500
N	2.36197600	-0.56363400	1.47509900
C	-2.46767600	-1.80976300	0.59090400
C	2.45964900	-1.82100900	-0.58732200

C	0.58189000	-2.18866300	-1.92831900
C	0.57889300	2.92352700	-0.47038700
C	2.04464300	1.59770300	-1.69559100
C	-0.59217100	-2.17887200	1.93475200
C	-0.56646000	2.92665700	0.46755400
C	-2.03810600	1.60922200	1.69460000
C	3.13232600	-1.02552900	0.48403600
C	2.93458200	0.13296900	2.45432200
C	-3.13296100	-2.86806900	1.21283200
C	3.12145300	-2.88278300	-1.20694300
C	1.18535400	-3.25698500	-2.57803200
H	-0.43291600	-1.86155400	-2.17129600
C	1.18215900	4.08270500	-0.95645700
C	2.69309200	2.71120600	-2.21705000
H	2.35334800	0.58861500	-1.97325800
C	-1.19931100	-3.24349700	2.58713400
H	0.42350000	-1.85412500	2.17735200
C	-1.16463700	4.08921800	0.95178500
C	-2.68174600	2.72637300	2.21424300
H	-2.35132200	0.60200700	1.97390000
C	4.51003800	-0.79216900	0.43156500
C	4.30247000	0.41078800	2.49513900
H	2.27566100	0.48531500	3.25271000
C	-2.49533500	-3.59291500	2.21666500
H	-4.13600800	-3.13994900	0.88567600
C	2.48078600	-3.60887100	-2.20795300
H	4.12425600	-3.15616100	-0.88026300
H	0.64549400	-3.79492900	-3.35638600
C	2.25322300	3.97628100	-1.83885500
H	0.82283600	5.06376800	-0.65150300
H	3.52386900	2.57876300	-2.90919300
H	-0.66177000	-3.78065200	3.36763300
C	-2.23631600	3.98891600	1.83417900
H	-0.80094200	5.06820500	0.64533100
H	-3.51319800	2.59857600	2.90645700
C	5.10237400	-0.05974000	1.45932700
H	5.10965500	-1.16018200	-0.40139900
H	4.72292500	0.98187600	3.32272200
H	-3.00607900	-4.42799200	2.69873700
H	2.98876400	-4.44670200	-2.68816000
H	2.73541000	4.87394200	-2.22763900
H	6.17481200	0.13918100	1.44683100
H	-2.71467500	4.88928800	2.22142500

$[Ru(bpy)_3]^+$

Nimag=0

Ru	-0.00008600	0.05809600	0.00002100
N	-0.84887400	-1.53847600	-1.02466000
C	-0.45580400	-2.77781000	-0.54739900
C	-0.96755000	-3.95200000	-1.17675700
C	-1.83878300	-3.85070700	-2.22989100
C	-2.23496000	-2.56853000	-2.69316300
C	-1.71068300	-1.46502700	-2.05214400
H	-0.66045500	-4.93218300	-0.81532300
H	-2.22516900	-4.75201000	-2.70720300
H	-2.92676200	-2.44525100	-3.52382700
H	-1.98087500	-0.45512200	-2.37424400
N	1.75930600	0.11353800	-1.13498000
N	-1.11836300	1.56448000	-0.98026300
N	0.84832700	-1.53870400	1.02474200
N	-1.75944000	0.11394000	1.13483700
N	1.11881100	1.56405500	0.98035600
C	0.45520000	-2.77793400	0.54726000
C	2.72063800	0.95477800	-0.69702900
C	2.00667300	-0.69182200	-2.17427500
C	-2.34935900	1.79036800	-0.47266400
C	-0.70041900	2.27494100	-2.03345400
C	1.71008300	-1.46549000	2.05228500
C	-2.72051400	0.95549100	0.69693900
C	-2.00723800	-0.69166100	2.17387300
C	2.34996600	1.78935600	0.47284700
C	0.70127600	2.27438300	2.03378500
C	0.96685700	-3.95226800	1.17642200
C	3.96890100	1.01189000	-1.32025400
C	3.22799400	-0.68854100	-2.84087800
H	1.19239100	-1.35915100	-2.46500600
C	-3.19632800	2.74950700	-1.03375100
C	-1.48817200	3.24509900	-2.64294800
H	0.30257300	2.04314600	-2.39889800
C	2.23427800	-2.56913900	2.69312500
H	1.98031400	-0.45566400	2.37459800
C	-3.96892600	1.01267400	1.31980200
C	-3.22872900	-0.68829400	2.84016100
H	-1.19317700	-1.35924700	2.46461000
C	3.19751900	2.74772900	1.03442000
C	1.48960200	3.24378800	2.64372100
H	-0.30189300	2.04309500	2.39907400
C	1.83806600	-3.85120900	2.22960000

H	0.65972600	-4.93237100	0.81480600
C	4.22479400	0.18064700	-2.40594500
H	4.73922100	1.69061200	-0.95886000
H	3.38692100	-1.36406700	-3.68047000
C	-2.76181600	3.48379900	-2.13177900
H	-4.18710700	2.92376900	-0.61827300
H	-1.10588800	3.79570500	-3.50159800
H	2.92605600	-2.44604600	3.52383800
C	-4.22526000	0.18117000	2.40521700
H	-4.73906100	1.69156900	0.95835000
H	-3.38797300	-1.36400900	3.67954500
C	2.76345300	3.48184000	2.13271100
H	4.18845500	2.92145000	0.61908100
H	1.10764000	3.79430100	3.50257000
H	2.22439700	-4.75261600	2.70676200
H	5.19563100	0.20866200	-2.90146700
H	-3.41187300	4.23430700	-2.58240600
H	3.41399800	4.23171200	2.58369000
H	-5.19622700	0.20919900	2.90048300

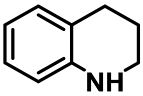

1a

Nimag=0

C	-2.55464700	0.70101800	-0.00738500
C	-1.34089400	1.38294200	-0.05125000
C	-0.11594500	0.71437600	-0.04972400
C	-0.11005000	-0.69665100	0.00947400
C	-1.33596400	-1.38343100	0.05759800
C	-2.54132400	-0.69318700	0.04599500
H	1.38554000	1.76625000	-1.17114400
H	-3.49715500	1.24876000	-0.01252800
H	-1.33391000	2.47554500	-0.09028100
C	1.19169400	1.47354600	-0.12469100
H	-1.32831800	-2.47472700	0.10947800
H	-3.47859200	-1.25112200	0.08250300
C	2.33048400	-0.74222000	-0.26870400
C	2.36109500	0.63394700	0.38464200
H	3.31849000	1.13060300	0.17348000
N	1.08422800	-1.39704000	0.06098100
H	1.01915700	-2.38334500	-0.15231500
H	1.10895700	2.40984800	0.44703400
H	2.28327400	0.50395300	1.47536600
H	2.46553900	-0.62677700	-1.36348600
H	3.16073300	-1.36174700	0.09928700

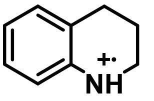

Int1

Nimag=0

C	-2.52042600	0.71926700	0.04052900
C	-1.30807000	1.42031000	-0.04247700
C	-0.09831600	0.75282600	-0.08835500
C	-0.12465600	-0.68996400	-0.04202600
C	-1.36918700	-1.39679400	0.04274300
C	-2.54476800	-0.69519500	0.08156900
H	1.44732200	1.59727900	-1.28017500
H	-3.45940800	1.27307200	0.07634400
H	-1.31628900	2.51108500	-0.07113600
C	1.22692200	1.44962300	-0.20744500
H	-1.36091600	-2.48797400	0.07749500
H	-3.49698600	-1.22161600	0.14685000
C	2.35422000	-0.77819900	-0.14476300
C	2.33725600	0.62506600	0.43938700
H	3.31651000	1.09117700	0.27397000
N	1.02025800	-1.37461800	-0.08147500
H	0.95865400	-2.39222200	-0.08774100
H	1.15994500	2.45113900	0.23722900
H	2.18089700	0.56777300	1.52743700
H	2.67130700	-0.76626300	-1.20214200
H	3.03931200	-1.44276900	0.39723300

O_2

Nimag=0

O	0.00000000	0.00000000	0.59150100
O	0.00000000	0.00000000	-0.59150100

O_2-

Nimag=0

O	0.00000000	0.00000000	0.65733400
O	0.00000000	0.00000000	-0.65733400

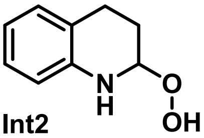

Int2

Nimag=0

C	-3.29739600	0.20427500	0.44874300
C	-2.31269000	1.17091900	0.23622300
C	-1.02608300	0.81446000	-0.15578800
C	-0.70664000	-0.54854200	-0.31618500
C	-1.69116100	-1.52028000	-0.10068500
C	-2.97855300	-1.14072100	0.27199600
H	0.19148900	1.95101800	-1.51204600
H	-4.30266500	0.49976500	0.74971200
H	-2.54826300	2.22981400	0.36808800
C	0.08146000	1.79843300	-0.42355000
H	-1.44020000	-2.57569400	-0.22564700
H	-3.73606400	-1.90908700	0.43505200
C	1.73425800	-0.10562500	-0.47560000
C	1.38471500	1.24423100	0.14715600
H	2.22672000	1.93374100	0.00452500
N	0.56890300	-0.88589400	-0.75248800
H	0.75421200	-1.86849300	-0.91420800
H	-0.15333900	2.77657300	0.01764500
H	1.25664400	1.07661800	1.22897300
H	2.34231600	0.02789900	-1.38792600
O	2.51938900	-0.89558000	0.41941700
O	3.79350000	-0.29083000	0.49414000
H	3.79624900	0.04748600	1.40094100

DBU

Nimag=0

C	-0.84699200	1.46105500	0.26573100
C	-2.10669700	1.13495400	-0.53308000
C	-0.92254200	-1.47823700	-0.39647000
C	-2.94393600	0.00304900	0.06400700
C	-2.07981700	-1.15850800	0.55461800
H	-1.07455400	1.43315000	1.35153500
H	-1.23601000	-1.31431300	-1.44116700
H	-1.80066100	0.89831600	-1.56338100
H	-3.65487700	-0.35606500	-0.69701900
H	-0.56427000	2.50083000	0.04602600
H	-2.72009100	2.04586000	-0.59742900
H	-0.62795500	-2.52962600	-0.31563300
H	-3.55250600	0.38907900	0.89721200
H	-2.70621000	-2.05274100	0.68532800
H	-1.66996000	-0.93285900	1.55265400
C	1.57896400	1.34404900	0.30223600
C	2.77013200	0.61472700	-0.29060300
C	2.71672200	-0.83770200	0.15919100
H	1.52422500	2.37417900	-0.08240100
H	2.71014700	0.66462700	-1.38913000
H	3.47298600	-1.43618500	-0.37186600
N	0.33733600	0.66731600	-0.04430300
H	2.97571600	-0.90927800	1.23120000
H	3.70668700	1.10143200	0.01702900
H	1.69028200	1.41812400	1.40290100
C	0.36911500	-0.72343500	-0.13273800
N	1.42228600	-1.44649500	-0.05329900

DBU^{+}

Nimag=0

C	-0.87528200	1.46870500	0.35716800
C	-2.03846900	1.14960500	-0.57767900
C	-0.90645400	-1.49790800	-0.35001300
C	-2.93934200	0.01673500	-0.08597900
C	-2.14984800	-1.16075700	0.48427800
H	-1.17580100	1.38995700	1.41530300
H	-1.11566100	-1.37710900	-1.42594700
H	-1.63370500	0.93716200	-1.57956600
H	-3.56627600	-0.32328900	-0.92322200
H	-0.53629900	2.49831400	0.19776000
H	-2.62809800	2.07021400	-0.68835400
H	-0.60324900	-2.54055700	-0.20033300
H	-3.62909900	0.39438300	0.68317600
H	-2.78626000	-2.05412800	0.52881000
H	-1.84476800	-0.96378500	1.52432900
C	1.62800500	1.31595600	0.35064100
C	2.71180800	0.61094600	-0.44322300
C	2.75278500	-0.84665400	-0.01112000
H	1.49733200	2.35044400	0.01302000
H	2.48775300	0.68920800	-1.51733100
H	3.26285900	-1.49759000	-0.74500800
N	0.33317500	0.63331000	0.16268300
H	3.30218000	-1.00803100	0.93295400
H	3.67970000	1.09694200	-0.26749800
H	1.86040400	1.34197200	1.42882400
C	0.31995300	-0.68442800	-0.02371700
N	1.43969000	-1.38149600	0.18316700

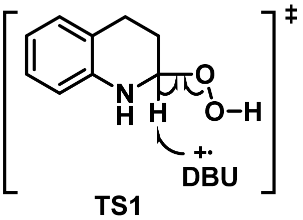

TS1

Nimag=1

C	5.12919700	-1.62426700	0.60949400
C	4.01508300	-1.29355000	1.38916000
C	3.04765900	-0.41538700	0.92250100
C	3.23078100	0.14401200	-0.37820100
C	4.35922500	-0.19502700	-1.16943800
C	5.29877100	-1.07255900	-0.67181000
H	1.02116200	-0.78416700	1.48313900
H	5.87560500	-2.31511200	1.00294200
H	3.90203200	-1.72855200	2.38368000
C	1.81570100	-0.05017600	1.70798600
H	4.47243600	0.25004200	-2.15969800
H	6.17215800	-1.33377000	-1.26913300
C	1.12264800	1.41656500	-0.16438500
C	1.31592100	1.34668500	1.34097100
H	0.34861200	1.55164600	1.81871400
N	2.30774300	1.01199200	-0.85465600
H	2.43620200	1.41580300	-1.78370400
H	2.02502200	-0.12551800	2.78287400
H	2.03024500	2.12400200	1.65157900
H	0.24657000	0.64468000	-0.43288400
O	0.67926300	2.61634800	-0.67942000
O	1.65892800	3.61140000	-0.36479900
H	1.10203100	4.40376700	-0.32663500
C	-4.50260700	-1.11199800	-0.01837800
C	-4.94436400	-0.07016400	1.00378800
C	-2.46961500	1.11962200	0.17018400
C	-4.98753900	1.35339200	0.44993300
C	-3.77327300	1.66339400	-0.42311500
H	-4.96982800	-0.90857000	-0.99928300
H	-2.49842100	1.18795900	1.27063000
H	-4.27857400	-0.13899100	1.87795900
H	-5.03841100	2.06519500	1.28805000
H	-4.87765400	-2.09239100	0.30237200
H	-5.94310700	-0.35371500	1.36449700
H	-1.62368600	1.73228400	-0.16357700
H	-5.90734500	1.49976300	-0.13613100
H	-3.67989700	2.74945500	-0.55967000
H	-3.90901200	1.24658400	-1.43393600
C	-2.67279200	-2.64311500	-0.60333900
C	-1.20622000	-2.91350900	-0.32238900
C	-0.38570100	-1.80274800	-0.95590000
H	-3.30326800	-3.35479800	-0.05357400
H	-1.03836400	-2.92708800	0.76586100
H	0.67524500	-1.88026400	-0.66291000
N	-3.05262500	-1.29280400	-0.18245000
H	-0.40970000	-1.89173200	-2.05587100
H	-0.92202100	-3.89738500	-0.71845900
H	-2.89176600	-2.77796900	-1.67759300
C	-2.11751400	-0.30572400	-0.22047900
N	-0.87996800	-0.49859400	-0.56360500

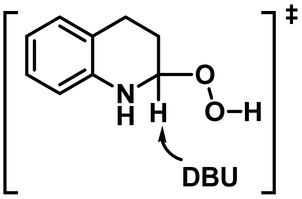

TS2

Nimag=1

C	-4.54070200	-1.31589300	-1.18831600
C	-3.96008500	-0.07130000	-1.42791600
C	-3.01010000	0.48547600	-0.57132700
C	-2.63007600	-0.24237700	0.58554100
C	-3.22008300	-1.49961500	0.82791800
C	-4.16157200	-2.02500100	-0.04693200
H	-1.60464800	1.71110400	-1.63338900
H	-5.28068700	-1.72319300	-1.87734100
H	-4.25413400	0.50013300	-2.31275600
C	-2.38959300	1.83639700	-0.86790400
H	-2.92576400	-2.05492900	1.72160900
H	-4.60427500	-2.99986200	0.16613500
C	-0.90593700	1.44005200	1.08990400
C	-1.76077600	2.48276600	0.37235700
H	-1.13780000	3.34400500	0.09489800
N	-1.70759500	0.28819200	1.44518200
H	-1.44030100	-0.21524600	2.28086500
H	-3.15088300	2.50063700	-1.30472600
H	-2.54727700	2.84198900	1.05835000
H	-0.08886400	1.12263300	0.03396900
O	-0.11096300	1.84634700	2.02811800
O	1.45924700	1.09824400	1.87746300
H	1.66680600	1.27300700	2.80430400

C	3.64441600	-1.41405000	-0.33904300
C	3.08565500	-2.12525300	0.89064600
C	0.60404300	-1.26940600	-0.23147700
C	2.05098600	-3.19308100	0.53888500
C	1.07734800	-2.68811600	-0.52359200
H	3.81182100	-2.13063500	-1.16328600
H	0.40988300	-1.12730700	0.84441700
H	2.65062200	-1.35169800	1.54457300
H	1.49633600	-3.47390000	1.44779700
H	4.62797900	-0.99753400	-0.08511500
H	3.92134400	-2.57987200	1.44225300
H	-0.35264300	-1.06931400	-0.73349700
H	2.55456700	-4.10588800	0.18303400
H	0.20033400	-3.34834100	-0.57587500
H	1.54381100	-2.72315100	-1.52142900
C	3.57319900	0.87805000	-1.32914600
C	3.09105000	2.14475400	-0.64009300
C	1.59975000	2.28309800	-0.89831500
H	4.64398200	0.71143300	-1.16318500
H	3.24686800	2.03765900	0.44220600
H	1.15203200	2.94467500	-0.13992200
N	2.84449100	-0.27275800	-0.79708900
H	1.40619600	2.72686300	-1.88912000
H	3.63900000	3.02291900	-1.00875600
H	3.41356200	0.94616700	-2.41936300
C	1.51240500	-0.12750600	-0.63649200
N	0.89971700	1.01127300	-0.80659100

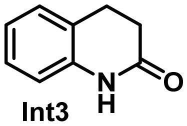

Int3

Nimag=0

C	-2.90884100	0.37919600	0.09621100
C	-1.82835600	1.25432100	-0.01977900
C	-0.52289900	0.77942500	-0.12341500
C	-0.30364600	-0.60883600	-0.09156100
C	-1.38083900	-1.49142900	0.02412400
C	-2.67922200	-0.99519600	0.11138100
H	0.90839400	1.73859500	-1.39000400
H	-3.92394600	0.76939000	0.17300700
H	-1.99872900	2.33318700	-0.03923400
C	0.67832200	1.66981200	-0.31230800
H	-1.19515100	-2.56697200	0.04979500
H	-3.51469500	-1.69087600	0.20061800
C	2.14832100	-0.34941500	0.05115800
C	1.88805200	1.09790800	0.42304200
H	2.81153500	1.65396700	0.22417300
N	1.00836200	-1.08748200	-0.18929500
H	1.16264900	-2.07831300	-0.34422200
H	0.45867700	2.68931400	0.03201300
H	1.71067400	1.11999900	1.51169300
O	3.24708900	-0.84632900	-0.00573600

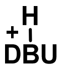

Nimag=0

C	-0.90573700	1.50706900	0.20916200
C	-2.08520000	1.08964200	-0.65877100
C	-0.93753600	-1.50362000	-0.25443700
C	-2.96560400	0.00964900	-0.03361700
C	-2.13744200	-1.10145700	0.60753200
H	-1.20431000	1.60963300	1.26575900
H	-1.20002600	-1.48161000	-1.32497500
H	-1.70340800	0.77842200	-1.64392200
H	-3.62273800	-0.41111500	-0.80899000
H	-0.55693200	2.49330000	-0.11915900
H	-2.68457700	1.99082100	-0.84830500
H	-0.64776600	-2.53958400	-0.03414400
H	-3.62652000	0.45397700	0.72474500
H	-2.76114700	-1.98958000	0.77321500
H	-1.77960500	-0.79710300	1.60389700
C	1.56588100	1.33016100	0.44604000
C	2.71998900	0.70825800	-0.31979300
C	2.79492700	-0.76923800	0.00855000
H	1.44269000	2.38353300	0.17170100
H	2.56628200	0.85243000	-1.39928300
H	3.44602800	-1.30508500	-0.69369000
N	0.29632100	0.64183800	0.14232100
H	3.18504300	-0.93369400	1.02438600
H	3.65901900	1.20558100	-0.04757700
H	1.73993200	1.28743200	1.53362800
C	0.30559500	-0.66602500	-0.05915600
N	1.45240200	-1.33923800	-0.08998600
H	1.37773200	-2.34218200	-0.21669000

HO•

Nimag=0

O	0.00000000	0.00000000	0.10842700
H	0.00000000	0.00000000	-0.86741500

HOO•

Nimag=0

O	0.05508200	0.70274200	0.00000000
O	0.05508200	-0.59392900	0.00000000
H	-0.88130500	-0.87050500	0.00000000

H_2O

Nimag=0

O	0.00000000	0.00000000	0.11919500
H	0.00000000	0.75599000	-0.47678000
H	0.00000000	-0.75599000	-0.47678000

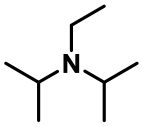

DIPEA

Nimag=0

N	-0.00405500	-0.26426200	-0.15034800
C	-0.36506300	-1.42208000	0.64603700
H	-1.46159900	-1.46014200	0.72117200
H	0.00377200	-1.34270600	1.69169300
C	0.11032000	-2.73353000	0.02946900
H	-0.19671200	-3.59051600	0.64633900
H	-0.31687800	-2.84835900	-0.97659500
H	1.20521800	-2.76807100	-0.06455000
C	1.39573300	0.13839800	-0.04509200
H	1.95841600	-0.79462000	0.11778200
C	-0.99864700	0.80526900	-0.22273700
H	-0.47021500	1.68927500	-0.61321000
C	1.71391500	1.06514000	1.13564600
H	1.36444700	0.63666300	2.08692700
H	2.79873700	1.22722800	1.21714900
H	1.23922600	2.05011100	1.00524300
C	1.90103500	0.72938800	-1.36075500
H	1.69932000	0.03481400	-2.18735900
H	1.40710600	1.68625900	-1.58912900
H	2.98267900	0.92218200	-1.31171000
C	-2.08509100	0.44526700	-1.23650900
H	-2.62791900	-0.45908300	-0.91891200
H	-2.82034700	1.25708400	-1.33949700
H	-1.63494300	0.24221800	-2.21742700
C	-1.62513500	1.20896000	1.12037400
H	-2.26105700	2.09713600	0.99137800
H	-2.26222100	0.40639700	1.52125400
H	-0.86105200	1.44309300	1.87329500

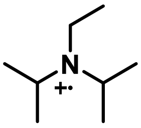

Nimag=0

N	0.00192100	-0.10870400	0.26070700
C	-0.58513100	-1.18684200	1.02863600
H	-1.63682600	-0.94854400	1.22494700
H	-0.04791500	-1.22372500	1.99136800
C	-0.45278000	-2.53414200	0.30535900
H	-0.89781300	-3.30945400	0.94156400
H	-0.98799000	-2.52078000	-0.65260400
H	0.59793100	-2.79950800	0.12986600
C	1.45607200	-0.05920100	0.11548800
H	1.84034400	-0.91755300	0.68430700
C	-0.84242600	0.90729400	-0.36381200
H	-0.16362600	1.55781800	-0.92980200
C	2.00118000	1.23008400	0.74063600
H	1.71227900	1.31825900	1.79647700
H	3.09733200	1.19576200	0.68825500
H	1.66462800	2.12240000	0.19515400
C	1.84989200	-0.21924800	-1.35709900
H	1.44177600	-1.14497800	-1.78434600
H	1.52070800	0.63535200	-1.96376700
H	2.94532400	-0.26914800	-1.41333900
C	-1.83183600	0.25443800	-1.33403600
H	-2.54835200	-0.39446500	-0.81131500
H	-2.40123400	1.05256200	-1.82827400
H	-1.31250200	-0.32545000	-2.10869200
C	-1.53998400	1.74563900	0.71586500
H	-2.08113900	2.55912600	0.21494600
H	-2.27068400	1.15428700	1.28384700
H	-0.81560400	2.19084000	1.41024700

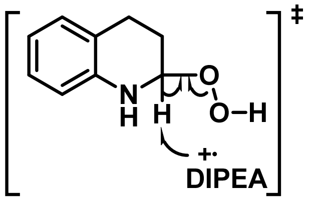

Nimag=1

C	4.63205700	-1.33406500	0.30402800
C	3.72747000	-0.86142100	1.25523500
C	2.59942000	-0.13577500	0.88036800
C	2.39809100	0.11292300	-0.49578400
C	3.30473000	-0.36245900	-1.45927200
C	4.41855300	-1.08137200	-1.05507000
H	0.84858400	-0.42786800	2.08165200
H	5.50966700	-1.89687500	0.62260900
H	3.90290200	-1.05546300	2.31518500
C	1.58728600	0.36971600	1.87897600
H	3.12817400	-0.15510200	-2.51650400
H	5.12726200	-1.44503200	-1.79922400
C	0.31713000	1.34107100	-0.02915200
C	0.87195100	1.61316000	1.35669000
H	0.05718400	1.92406500	2.02264500
N	1.28101500	0.82537400	-0.89715900
H	1.17352300	1.05535300	-1.88340300
H	2.08133400	0.58376000	2.83572100
H	1.57500600	2.45727500	1.26593300
H	-0.66466300	0.48342400	0.06735500
O	-0.38947700	2.36611100	-0.63186800
O	0.52642500	3.43564300	-0.89530600
H	-0.01988100	4.20431500	-0.66803800
N	-1.86212700	-0.28861900	-0.10738000
C	-1.83269600	-0.32968300	-1.58221800
H	-0.78236400	-0.49626900	-1.87439800
H	-2.08457800	0.68910400	-1.91164900
C	-2.70467600	-1.35509500	-2.30242700
H	-2.65964500	-1.14416700	-3.37956700
H	-2.34483800	-2.38044800	-2.15529200
H	-3.75545700	-1.30907300	-1.99456100
C	-2.87959100	0.65005100	0.46169400
H	-2.56201900	1.63306400	0.07833100
C	-1.57981400	-1.55191600	0.62772400
H	-1.29440200	-1.22551300	1.63930500
C	-2.80578000	0.70835400	1.98263300
H	-1.77712600	0.85881700	2.33967500
H	-3.40610800	1.55861200	2.33120200
H	-3.20808100	-0.19650800	2.45788700
C	-4.30866500	0.42043400	-0.03129000
H	-4.39557300	0.56906500	-1.11498100
H	-4.68971700	-0.57726000	0.22171600
H	-4.96177900	1.16124300	0.44991500
C	-2.77690300	-2.49336500	0.78199800
H	-3.16430000	-2.83935600	-0.18368600
H	-2.44926700	-3.37678000	1.34743200
H	-3.59753600	-2.03189500	1.34369500
C	-0.37388200	-2.27302700	0.03128500
H	-0.07543400	-3.08393700	0.70868700
H	-0.59926600	-2.72616600	-0.94355100
H	0.49253100	-1.60688700	-0.08742300

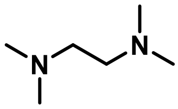

TEMED

Nimag=0

N	-1.86120400	0.00000000	-0.45784500
C	-0.42137300	-0.00000900	-0.64395200
H	-0.16047800	-0.88351200	-1.25071900
H	-0.16046900	0.88346900	-1.25075100
C	0.42137300	0.00000900	0.64395300
H	0.16046900	-0.88347000	1.25075100
H	0.16047800	0.88351200	1.25072000
N	1.86120400	0.00000000	0.45784400
C	2.39239800	-1.20188700	-0.13914500
H	2.17726000	-1.30483700	-1.22458300
H	3.48678100	-1.22207600	-0.02080500
H	1.98310000	-2.08555100	0.37262800
C	2.39241100	1.20187900	-0.13915100
H	3.48679500	1.22205100	-0.02082100
H	2.17726500	1.30483000	-1.22458700
H	1.98313200	2.08554900	0.37262500
C	-2.39241200	-1.20187900	0.13915100
H	-2.17726400	-1.30483100	1.22458600
H	-3.48679600	-1.22204900	0.02082200
H	-1.98313400	-2.08554900	-0.37262600
C	-2.39239800	1.20188800	0.13914500
H	-1.98309800	2.08555100	-0.37262700
H	-3.48678000	1.22207800	0.02080400
H	-2.17726000	1.30483600	1.22458300

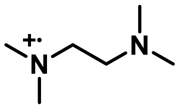

Nimag=0

N	1.83511900	-0.00001300	0.30993400
C	0.50019800	-0.00007700	0.67545100
H	0.22844800	-0.90773800	1.23130300
H	0.22842900	0.90749500	1.23143100
C	-0.50045400	0.00000200	-0.67566100
H	-0.22894300	-0.90761100	-1.23167500
H	-0.22889100	0.90763700	-1.23162100
N	-1.83516400	0.00000300	-0.30992700
C	-2.46233100	-1.23293000	0.11444500
H	-2.37930300	-1.36885400	1.20882800
H	-3.53044200	-1.20844900	-0.14091300
H	-1.99435300	-2.08599400	-0.39138400
C	-2.46231300	1.23297400	0.11431100
H	-3.53054700	1.20819200	-0.14047900
H	-2.37872700	1.36930700	1.20860300
H	-1.99474200	2.08593400	-0.39207800
C	2.46245700	-1.23291500	-0.11438300
H	2.37913900	-1.36905200	-1.20870600
H	3.53061000	-1.20812700	0.14071300
H	1.99478700	-2.08595900	0.39177600
C	2.46248200	1.23294700	-0.11421500
H	1.99399300	2.08602000	0.39112600
H	3.53039000	1.20866000	0.14199900
H	2.38023700	1.36860600	-1.20867000

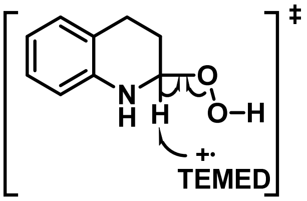

Nimag=1

C	-4.51720400	1.95925800	0.52377800
C	-4.21213400	0.65047600	0.90116600
C	-3.09585700	-0.00546500	0.39040300
C	-2.28023800	0.69258100	-0.53113000
C	-2.58209500	2.01308500	-0.91389400
C	-3.69892000	2.63913900	-0.38572900
H	-2.08455700	-1.36858700	1.69733900
H	-5.39877600	2.45063800	0.93597300
H	-4.85778700	0.12441600	1.60693600
C	-2.71641100	-1.40904500	0.79222000
H	-1.93876000	2.52894400	-1.62946900
H	-3.94002400	3.65928300	-0.68475900
C	-0.78790600	-1.24810100	-0.76449800
C	-1.95796600	-2.11528600	-0.32935000
H	-1.57575200	-3.09292400	-0.01039800
N	-1.16769800	0.06673400	-1.05767100
H	-0.59053000	0.56447500	-1.73372300
H	-3.61495700	-1.97655800	1.06675200
H	-2.60620000	-2.26473700	-1.20834800
H	0.01694300	-1.22234200	0.12868800
O	-0.08461200	-1.70369100	-1.88303300
O	0.73776500	-2.76448700	-1.43808400
H	0.64603100	-3.39127200	-2.17319600
N	1.30355500	-0.93862400	1.05998000
C	2.01807300	-0.09547500	0.10784700
H	1.37077500	0.76831000	-0.11407200
H	2.14880500	-0.66746900	-0.82324700
C	3.39343400	0.40491100	0.57590800
H	3.32544600	0.82945900	1.60148700
H	4.07628000	-0.45579800	0.63229700
C	1.96161000	-2.19622000	1.38914800
H	2.32796500	-2.66282000	0.46758800
C	0.73735700	-0.24121900	2.20446300
H	0.12169000	-0.93693500	2.79052300
H	1.24291700	-2.87154600	1.87306300
H	0.11155100	0.59358800	1.85612100
H	2.80501300	-2.03087400	2.08206900
H	1.52296300	0.16062300	2.86724400
N	3.91478900	1.35345800	-0.37874200
C	3.41444300	2.69627300	-0.16546600
H	3.78500300	3.14202600	0.78161100
H	3.72444500	3.34742600	-0.99387000
H	2.31501400	2.70435000	-0.13346800
C	5.36230300	1.33201400	-0.48090500
H	5.68295900	2.01092100	-1.28252000
H	5.86420500	1.64882400	0.45643900
H	5.70866000	0.32146200	-0.73687700

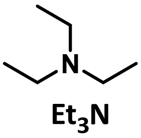

Nimag=0

N	-0.00003100	-0.00000900	0.02913000
C	1.09998300	-0.85841100	0.44460400
H	2.02890800	-0.27209900	0.39492100
H	0.98700200	-1.16083600	1.50979400
C	1.26064000	-2.08762200	-0.43912900
H	1.42266300	-1.77928800	-1.48106800
H	2.11738300	-2.69315400	-0.11114200
H	0.37036500	-2.73197500	-0.41303400
C	-1.29345400	-0.52343100	0.44457100
H	-1.25016800	-1.62105200	0.39486200
H	-1.49886000	-0.27439900	1.50976700
C	-2.43828100	-0.04786700	-0.43915300
H	-2.25236100	-0.34242800	-1.48108500
H	-3.39111300	-0.48686700	-0.11107600
H	-2.55095800	1.04533400	-0.41309400
C	0.19334800	1.38185900	0.44451200
H	-0.77884600	1.89320600	0.39459300
H	0.51153600	1.43531000	1.50976100
C	1.17776500	2.13546600	-0.43909300
H	1.27393800	3.18015700	-0.11105300
H	2.18084700	1.68646200	-0.41285600
H	0.82987000	2.12172700	-1.48108100

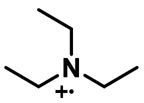

Nimag=0

N	-0.00005100	-0.00005500	0.02075800
C	-0.42276300	1.38977500	0.07486000
H	-1.20594100	1.51133600	-0.69115600
H	-0.95050600	1.50700400	1.04215100
C	0.66618000	2.43214800	-0.08144600
H	1.15745000	2.37034900	-1.06163700
H	0.19531300	3.42057400	-0.01181800
H	1.42471600	2.37253400	0.70909600
C	1.41492900	-0.32878600	0.07613400
H	1.91264000	0.28990600	-0.68833400
H	1.77881500	0.06833700	1.04452100
C	1.77367100	-1.79278700	-0.08151100
H	1.47659600	-2.18593500	-1.06288500
H	2.86496200	-1.87920500	-0.00968500
H	1.34120100	-2.42111700	0.70719500
C	-0.99229000	-1.06112100	0.07411300
H	-0.70659300	-1.79914800	-0.69316300
H	-0.82912000	-1.57807700	1.04050800
C	-2.43966500	-0.63919000	-0.08050300
H	-3.06022600	-1.54115000	-0.01023700
H	-2.76633300	0.04735800	0.71059500
H	-2.63299200	-0.18261200	-1.06034100

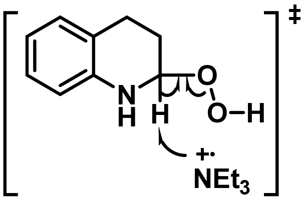

Nimag=1

C	-4.51608700	-1.44438400	-0.02795600
C	-3.64671500	-1.09788800	-1.06284600
C	-2.49411800	-0.35556100	-0.81908600
C	-2.23228700	0.04293000	0.51123400
C	-3.10459600	-0.30446400	1.55931500
C	-4.24259400	-1.04481600	1.28502000
H	-0.81838500	-0.82767000	-2.06183300
H	-5.41371600	-2.02370400	-0.24522800
H	-3.87016700	-1.40695300	-2.08578500
C	-1.51992300	0.01158800	-1.91028700
H	-2.88166900	0.01956000	2.57778900
H	-4.92442900	-1.30997700	2.09311700
C	-0.16705300	1.19913100	-0.17079700
C	-0.74434000	1.28347400	-1.57190400
H	0.07015500	1.45716500	-2.28789500
N	-1.10309700	0.79198000	0.78262600
H	-0.96126800	1.13758300	1.73053200
H	-2.05538000	0.13584600	-2.86068300
H	-1.40970300	2.16195200	-1.59472800
H	0.81289500	0.36803000	-0.17089800
O	0.54343500	2.30058200	0.27277200
O	-0.37350400	3.39239400	0.40461100
H	0.16952700	4.12777900	0.08025900
N	2.04574000	-0.34140500	-0.01002900
C	2.30426800	-0.02864500	1.40581900
H	1.32353200	-0.05486100	1.91349700
H	2.64369700	1.01654200	1.44091100
C	3.26535800	-0.94964200	2.14704800
H	3.38031500	-0.58281100	3.17575700
H	2.87795300	-1.97579200	2.20569200
H	4.25968800	-0.97473600	1.68422200
C	2.90759200	0.33533400	-0.99940000

H	2.61693000	1.39835200	-0.98982100
C	1.65692700	-1.73004700	-0.31113000
H	0.95506100	-1.68833000	-1.16038900
C	4.41083000	0.22794200	-0.77450100
H	4.71075400	0.71478000	0.16338200
H	4.76188600	-0.81132200	-0.76000300
H	4.92692300	0.74833800	-1.59240700
C	2.78482400	-2.69499300	-0.65818400
H	3.52742300	-2.77825400	0.14479300
H	2.35558500	-3.69148500	-0.82813100
H	3.29761500	-2.39642700	-1.58250800
H	2.63103000	-0.06724200	-1.98598500
H	1.08327800	-2.09395900	0.55502400

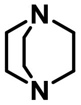

DABCO

Nimag=0

C	-0.77944500	-1.20989500	0.65408300
C	0.77980000	-1.20989400	0.65366400
H	-1.18459500	-1.23862300	1.67679300
H	-1.18504200	-2.08143600	0.11854800
H	1.18552500	-1.23938200	1.67612200
H	1.18508700	-2.08104500	0.11725400
C	-0.77982000	1.17125000	0.72057700
H	-1.18509300	2.07116000	0.23394500
H	-1.18563100	1.14326500	1.74304400
C	0.77939900	1.17122500	0.72106800
H	1.18502700	2.07147900	0.23537100
H	1.18454000	1.14246600	1.74378100
C	0.77965700	0.03903600	-1.37467500
H	1.18467800	0.93922100	-1.86100200
H	1.18555500	-0.83161700	-1.91142400
C	-0.77959100	0.03827600	-1.37473300
H	-1.18547600	0.93770500	-1.86173600
H	-1.18457700	-0.83316900	-1.91088900
N	-1.27224500	-0.00020500	-0.00001500
N	1.27224600	0.00020400	0.00005600

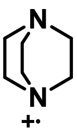

Nimag=0

C	-0.81881300	0.03687100	-1.36895500
C	0.81774200	0.03735100	-1.36957100
H	-1.18323200	-0.84737600	-1.90419700
H	-1.18376400	0.94840300	-1.85586000
H	1.18224400	-0.84655400	-1.90532300
H	1.18181000	0.94921100	-1.85652100
C	-0.81777800	-1.20450600	0.65287100
H	-1.18172900	-1.22624300	1.68642100
H	-1.18236100	-2.08216300	0.10701800
C	0.81871700	-1.20430300	0.65209000
H	1.18364400	-1.22619300	1.68529300
H	1.18302400	-2.08172900	0.10568000
C	0.81831900	1.16739400	0.71653800
H	1.18309800	1.13321300	1.74945300
H	1.18249900	2.07327900	0.21863000
C	-0.81818700	1.16722500	0.71696400
H	-1.18240100	1.13324300	1.75008400
H	-1.18283200	2.07290000	0.21900900
N	-1.21752300	-0.00022700	0.00050900
N	1.21752300	0.00020100	-0.00041000

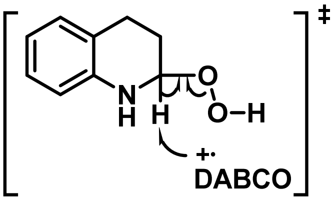

Nimag=1

C	4.52323900	-1.77580100	0.04111600
C	3.70897200	-1.30427200	1.07243100
C	2.63532100	-0.45777700	0.81282500
C	2.39596300	-0.08367900	-0.53044200
C	3.21217300	-0.55839400	-1.57463900
C	4.27237500	-1.40058100	-1.28376100
H	0.90584500	-0.68835500	2.05060100
H	5.36057600	-2.43519800	0.27065800
H	3.91485600	-1.59738500	2.10365900
C	1.71376200	0.04937000	1.89424700
H	3.00891300	-0.25066000	-2.60223500
H	4.91196300	-1.76457900	-2.08788800
C	0.47988400	1.30615700	0.14273900
C	1.10702200	1.40089600	1.52021100
H	0.33884200	1.70814600	2.24257400
N	1.34581000	0.76459900	-0.81339000

H	1.19771500	1.06014600	-1.77733400
H	2.25684200	0.12017700	2.84558300
H	1.88078400	2.18516900	1.48822400
H	-0.54595700	0.55343000	0.24028600
O	-0.15713300	2.43018500	-0.34537900
O	0.83095200	3.44702100	-0.54496000
H	0.35885900	4.22919600	-0.21901400
C	-2.87740600	0.80446800	0.65311800
C	-4.26260500	0.12939400	0.38594900
H	-2.69288600	0.96532500	1.72434400
H	-2.76701900	1.76882300	0.13907400
H	-4.80149800	-0.03866600	1.32784700
H	-4.89132300	0.77048100	-0.24613600
C	-1.87936500	-1.39144000	0.84306100
H	-1.05314800	-2.01527900	0.47271700
H	-1.71915400	-1.20597300	1.91442400
C	-3.28041800	-2.03329200	0.56043500
H	-3.16768700	-3.00173400	0.05515000
H	-3.82097700	-2.20904300	1.49989700
C	-3.39487700	-0.94477200	-1.55342900
H	-3.29149800	-1.91169900	-2.06346300
H	-4.01585900	-0.29834100	-2.18764200
C	-1.99329100	-0.29602500	-1.30882400
H	-1.17106900	-0.93887100	-1.65660800
H	-1.89213500	0.68724800	-1.79105000
N	-1.83061800	-0.09901700	0.14148100
N	-4.06662500	-1.14779700	-0.28111900

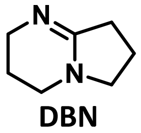

Nimag=0

C	-0.19381100	-0.73900500	0.03580900
C	0.97785000	1.40715900	0.19940900
C	2.12858600	0.61015100	-0.41069200
C	2.12616500	-0.82285000	0.12828600
C	-2.44502100	0.01673600	-0.30587900
H	0.86347300	2.38181200	-0.30160700
H	1.99656200	0.58730600	-1.50373800
H	2.47079300	-0.82417600	1.17850100
H	-3.44661600	0.02962600	0.14123600
H	-2.55748700	0.08833300	-1.39717100
N	0.83450300	-1.48711400	0.06208000
N	-0.23041300	0.64136400	0.02664900
H	1.17370000	1.61179100	1.27037400
H	3.08821000	1.10406900	-0.20250900
H	2.86007900	-1.42697300	-0.42656000
C	-1.62731300	-1.22896700	0.05502000
H	-1.76183700	-2.08051500	-0.62217200
H	-1.85142600	-1.57678100	1.07575200
C	-1.56443500	1.17438000	0.18635300
H	-1.78125900	1.42674100	1.24344000
H	-1.69494600	2.09340200	-0.40649200

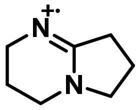

Nimag=0

C	-0.22921200	-0.70832400	0.06512400
C	1.00126400	1.39283800	0.21942100
C	2.10295200	0.61242200	-0.48557600
C	2.15650500	-0.81372300	0.05387600
C	-2.45254200	0.01179700	-0.33458300
H	0.81850800	2.36833600	-0.25015100
H	1.89977600	0.59908700	-1.56672600
H	2.66183600	-0.88584400	1.03635000
H	-3.44525900	-0.00548000	0.12727700
H	-2.58621500	0.11720100	-1.41916800
N	0.87133400	-1.42620500	0.23839400
N	-0.23182400	0.61720100	0.12769600
H	1.22763900	1.57005200	1.28466200
H	3.06854700	1.11202300	-0.33841600
H	2.74666900	-1.48630900	-0.59385800
C	-1.62679800	-1.24701700	-0.02327700
H	-1.70340800	-2.04412600	-0.77337500
H	-1.86826800	-1.69611300	0.95393500
C	-1.59265400	1.16830700	0.19499000
H	-1.81141600	1.43058900	1.24320400
H	-1.64206200	2.08580600	-0.40620900

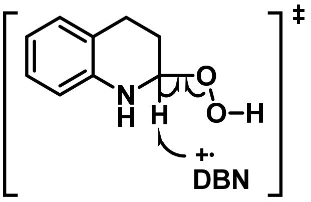

Nimag=1

C	4.79751200	-1.67558700	0.34127100

C	3.76979600	-1.33172700	1.22560100
C	2.75153000	-0.46932700	0.84273700
C	2.79234600	0.05924500	-0.47995000
C	3.83115500	-0.29201600	-1.37726200
C	4.82559100	-1.15379600	-0.96195700
H	0.79863600	-0.83808400	1.62290600
H	5.58653900	-2.35298100	0.66943300
H	3.76473200	-1.74327600	2.23643100
C	1.60547500	-0.09418300	1.74593800
H	3.83671100	0.13033700	-2.38384400
H	5.63230200	-1.42424600	-1.64309700
C	0.71402700	1.32480400	-0.07702900
C	1.05626200	1.28996600	1.40260500
H	0.14057600	1.49759400	1.97212200
N	1.81683200	0.91797900	-0.87553700
H	1.84511800	1.30079400	-1.82140900
H	1.92718800	-0.14199800	2.79428500
H	1.79006800	2.08084100	1.62172700
H	-0.18751500	0.51438100	-0.22812200
O	0.18856300	2.49995600	-0.57058900
O	1.17744000	3.52224900	-0.39628200
H	0.61255500	4.30378100	-0.29828400
C	-4.88291100	-0.02875200	0.16448100
C	-4.39138800	1.42311000	0.09111900
C	-2.89949000	1.31883000	0.43533900
H	-5.68040700	-0.24800400	-0.55953000
H	-2.73057600	1.36791300	1.52360300
H	-4.94536200	2.08855600	0.76249700
H	-5.25253500	-0.29806000	1.16928400
H	-4.51016100	1.79918800	-0.93424500
H	-2.26237900	2.07461300	-0.04132600
C	-3.67718900	-2.21936700	-0.41794800
C	-2.30076500	-2.78574100	-0.08059100
C	-1.20437700	-1.89655800	-0.66838500
H	-4.45501000	-2.69167500	0.20051500
H	-2.18041200	-2.82707300	1.01279600
H	-0.21270200	-2.26319300	-0.36305500
N	-3.68090200	-0.79051100	-0.15446400
H	-1.23450200	-1.94274500	-1.77119700
H	-2.21283700	-3.81102300	-0.46287900
H	-3.93634500	-2.40747900	-1.47308600
C	-2.54737400	-0.07533600	-0.02483900
N	-1.35062900	-0.51421000	-0.23247600

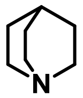

Quinuclidine

Nimag=0

C	0.79889000	-1.23849900	0.60057400
C	-1.28899100	-0.00186100	0.00059900
C	-0.75548300	-1.29684400	0.62931200
H	1.21636600	-2.07868300	0.02564300
H	1.21686300	-1.30807000	1.61604800
H	-1.13128800	-2.16608400	0.06845600
H	-1.13088600	-1.39344400	1.65941900
C	0.79592100	0.10049000	-1.37439300
H	1.21099200	1.01916200	-1.81537100
H	1.21416300	-0.74347800	-1.94318800
C	-0.75856800	0.10214100	-1.43628500
H	-1.13690700	1.02182800	-1.90789300
H	-1.13369200	-0.74230400	-2.03425500
H	-2.38840100	-0.00348900	0.00112800
C	-0.75911000	1.19150800	0.80801500
H	-1.13670900	2.13092500	0.37638900
H	-1.13538000	1.13943900	1.84100700
C	0.79540700	1.14139500	0.77271500
H	1.21128400	2.05669300	0.32551800
H	1.21245200	1.06462100	1.78805700
N	1.27753600	0.00184400	-0.00059800

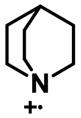

Nimag=0

C	0.82710600	1.15585900	-0.79076200
C	-1.27701200	0.00158800	0.00012800
C	-0.76141300	1.19094700	-0.81422600
H	1.24385900	2.05164400	-0.31565900
H	1.24334600	1.03880500	-1.79818600
H	-1.10248900	2.14369500	-0.38958500
H	-1.10309800	1.13985300	-1.85587600
C	0.82603400	0.10528600	1.39712600
H	1.24062600	-0.75484800	1.93576000
H	1.24369900	1.03548100	1.79982700
C	-0.76251900	0.11116100	1.43781300
H	-1.10582200	-0.73229300	2.05029000
H	-1.10284100	1.03949300	1.91403000

H	-2.37727600	0.00294700	0.00024300
C	-0.76450500	-1.29927900	-0.62333300
H	-1.10705900	-2.17519300	-0.05779200
H	-1.10707300	-1.40730900	-1.66033300
C	0.82408900	-1.26422700	-0.60662200
H	1.23962100	-2.07881900	-0.00195500
H	1.23956400	-1.30152500	-1.62045100
N	1.15489300	-0.00142000	-0.00015200

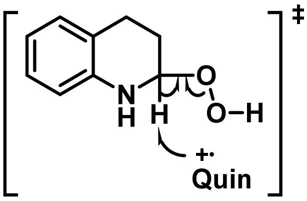

Nimag=1

C	4.68912500	-1.56737400	-0.31244200
C	3.81416700	-1.43774400	0.76890000
C	2.74761800	-0.54705100	0.72881700
C	2.57645700	0.23289300	-0.44529700
C	3.45488500	0.09697400	-1.54336000
C	4.50612400	-0.79737100	-1.46979700
H	0.97580500	-1.18414500	1.73470800
H	5.52044300	-2.27043100	-0.25395000
H	3.96672400	-2.04205700	1.66506200
C	1.75628200	-0.41204500	1.85692200
H	3.29999900	0.70831300	-2.43439000
H	5.19231500	-0.89984700	-2.31048100
C	0.56785100	1.28900200	0.50135200
C	1.10431800	0.96936600	1.88169800
H	0.27770800	1.00830400	2.60260300
N	1.55161600	1.13733800	-0.50278300
H	1.39751500	1.66717800	-1.36035500
H	2.25020700	-0.61900100	2.81519500
H	1.83403100	1.74708100	2.15854200
H	-0.33766000	0.54746800	0.26635700
O	0.02206300	2.56851700	0.49887900
O	-0.54864800	2.75000800	-0.79375700
H	-0.67969200	3.71132400	-0.79582300
C	-2.80320700	0.75711600	0.61613800
C	-4.08967700	-1.13947300	-0.38764500
C	-4.21792900	0.13787300	0.45287400
H	-2.55475000	0.93890000	1.67165200
H	-2.70217100	1.71162100	0.08251100
H	-4.65340000	-0.09983500	1.43382300
H	-4.89324600	0.85299500	-0.03664100
C	-1.86580400	-1.47012000	0.71151000
H	-1.01548100	-2.07439100	0.36222300
H	-1.74786400	-1.31418800	1.79405800
C	-3.22363200	-2.14965700	0.37661300
H	-3.06426400	-3.04902800	-0.23493000
H	-3.72851100	-2.46864500	1.29867700
H	-5.08270700	-1.56620300	-0.58136700
C	-3.39647200	-0.79354000	-1.71227900
H	-3.35515100	-1.67543200	-2.36621100
H	-3.96811000	-0.02034700	-2.24487400
C	-1.96247300	-0.28855200	-1.39284300
H	-1.19597700	-0.99000700	-1.75430700
H	-1.76092300	0.69443900	-1.84073500
N	-1.79561000	-0.15791700	0.06002600
